# Self‐Controlled Automated Strategy for the Synthesis of Gold Nanorods With Fine‐Tuned Longitudinal Absorption

**DOI:** 10.1002/chem.202502967

**Published:** 2026-01-20

**Authors:** Giordano Zanoni, Elisabetta Collini, Fabrizio Mancin

**Affiliations:** ^1^ Department of Chemical Sciences University of Padova Padova Italy

**Keywords:** automation, gold, nanorods, size tuning, synthesis

## Abstract

Reproducibility is the main weak point of most of the currently available synthetic strategies for metal nanostructures, and this issue is hampering both production and research on such materials. While several synthetic strategies can be found in literature, most of them are fundamentally based on strict protocols consisting of lists of subsequential predefined operations to be executed in a time‐defined manner. Such protocols are designed and optimized on the basis of pilot runs and are intrinsically affected by noncontrollable fluctuations in the experimental conditions. In this work an innovative, intrinsically flexible, automatized, and self‐correcting strategy is proposed, which, in combination with real‐time monitoring of the optical properties of the reaction mixture, allows to synthesize precisely tuned gold nanorods. This strategy is based on the fast and precisely controlled oxidation of precursor AuNRs. The fast reaction allows the selective and predictable etching of the nanorods by online‐controlled subsequential additions of small amounts of oxidants, which are also able to remove undesirably shaped byproducts possibly present in the sample. Due to these features, this process can be automated and allows starting from nonpurified nanorod dispersions with variable aspect‐ratio. Furthermore, the reaction can be stopped by oxidizer quenching, providing stable dispersions.

## Graphical abstract

This work proposes an innovative strategy which allows to synthesize precisely tuned gold nanorods starting from nonpurified dispersions with variable aspect‐ratio. The etching process is under direct control and can be stopped at any time by oxidizer quenching, providing stable dispersions.

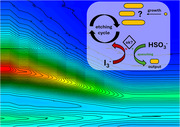

## 1 Introduction

The interest in gold nanostructures, and particularly in gold nanorods (AuNRs), remains very high, continuously prompted by their relevant and unique optical features [1, 2]. The most attractive property of AuNRs is their strong plasmonic absorption, whose energy depends on the size and aspect ratio of the nanostructure [3] and can be tuned to span specific regions of the electromagnetic spectrum. High aspect ratios significantly redshift the longitudinal plasmonic band, allowing it to cover even regions of the NIR spectrum. This is very convenient for optical and biomedical applications, for the development of tracers [4, 5, 6, 7], sensors [8, 9, 10] and photothermal systems [4, 11]. On the other hand, low AuNRs aspect ratios locate the longitudinal band in the visible region, where many organic dyes also absorb. This allows the realization of strongly coupled organic‐inorganic hybrids for the development of photonic applications such as plexcitonic systems [12, 13, 14, 15].

The significant interest generated by AuNRs starkly contrasts with the difficulties in controlling and reproducing their synthetic procedures, which often require specific hands‐on training and day‐to‐day adjustments. The reason for such poor reproducibility is intrinsically connected with the peculiar nature of nanoparticles, which are thermodynamically unstable entities formed under kinetic control and/or in oversaturation conditions, where even slight fluctuations in the conditions may lead to different outcomes. Further uncertainty, in the case of AuNRs, is added by the still scarce understanding of the mechanisms at play in their anisotropic growth [16, 17, 18, 19, 20, 21]. The logical consequence is that the formation of AuNRs is strongly affected by the reaction conditions, including several relevant parameters that are difficult to control and maintain stable, such as the number of nucleation points or the local temperature. Indeed, a few papers appeared over the years aiming to provide synthetic guidelines and suggesting practical advice for the synthesis of such nanostructures [22].

The need to address these reproducibility issues justifies the still firm interest in studying, improving, and standardizing the synthetic strategies for gold nanostructures [22, 23, 24]. With this goal, even a few robotic nano‐synthesizers have been recently developed to remove any operator‐dependent variability and improve reproducibility [25, 26, 27, 28, 29]. All of these procedures, however, are based on the precise execution of predefined protocols, including fixed reaction times, and cannot take into account the reaction fluctuations discussed above. In this scenario, the “ideal” synthetic strategy should be based on processes that can be monitored and controlled in real time by the operator or by an automated system, allowing real‐time decisions and safe checkpoints.

Most of the available AuNRs synthetic procedures are based on two subsequent steps [30]: the first is the formation of seeds (small gold clusters or nanoparticles) by fast reduction of oxidized gold precursors; the second is the anisotropic growth of these seeds, by reduction of additional gold precursor, which leads to the desired rod‐shaped structures. The anisotropic growth is usually promoted by the addition of Ag^+^ ions and by the presence of the CTAB surfactant, likely because of the preference of these species for absorption on specific faces that differentiates the deposition sites of the reduced gold atoms [17, 31].

In principle, the final aspect ratio can be controlled in different ways. The first and simplest approach is to terminate the reaction, for instance by addition of a quencher such as sulfide ions [32], or to wait for complete precursor consumption. Here, the obtained aspect ratio should depend on the initial concentrations of reagents and adjuvants and on the reaction time. However, the application of these strategies to the precise tuning of AuNRs is difficult for three reasons: i) the first stages of nucleation and nanorods growth are extremely fast and hence difficult to control, this is particularly true if low aspect ratios AuNRs are sought, ii) during AuNRs growth different shapes are involved at different time points, and an early termination of the reaction might lead to undesired shapes and structures [18] (see also infra), iii) quenchers can alter the final rod properties, as an example, sulfide ions have a strong affinity for gold surfaces and can affect the optical properties of the AuNRs, leading to a plasmonic band redshift [32], and they can also interfere with a subsequent surface post‐functionalization of the AuNRs.

Being direct control over the AuNRs growth process difficult, a more convenient strategy involves the introduction of a third re‐oxidation step. Since the AuNRs oxidation is favored at the rod terminations [33, 34], chemical etching leads to the homogeneous shortening of the rods. The process is generally slow and can in principle be stopped when needed. In this way, it is possible to tune the aspect ratio a posteriori [22], and this is actually the only available option for the synthesis of very short AuNRs, which cannot be obtained by direct synthesis.

Au(III), in the form of tetrachloroaurate, is the most commonly employed oxidant for AuNRs etching [33, 35]. [AuCl_4_]^−^ is not able to oxidize Au(0), but it is converted in situ into [AuBr_4_]^−^ (by the large amount of CTA bromide, usually 100 mM, present in the reaction mixture), which is conveniently reactive. However, while allowing to prepare nanorods with the desired aspect ratio, this reaction still does not fulfill the requisites for precise online control of the final product. The AuNRs oxidation with Au(III) is indeed slow, making it impractical to wait for the reaction to reach completion or to adjust the amount of oxidant added in real time. Consequently, reported procedures usually add a predetermined amount of oxidant for a fixed amount of time and then quickly isolate the product from the reaction mixture. It is quite self‐evident that fluctuations in the reaction rates are possible and that the sample can continue to evolve during the purification. For this reason, even when small scale pilot‐runs are performed in advance for each batch of precursor AuNRs, the aspect ratio obtained still suffers from some uncertainty.

In this paper, we aim to address the lack of reproducibility in the preparation of AuNRs with a predetermined and precise position of their plasmonic band by reporting a new, intrinsically self‐correcting, fully automated, and programmable strategy for the on‐demand synthesis of shortened AuNRs.

## 2 Results and Discussion

Our underlying idea was that full control of the oxidation‐based AuNRs size tuning can be granted by using a fast oxidant.

Indeed, as mentioned earlier, the necessary consequence of the use of slow and unquenchable oxidants, such as Au(III) salts, is a size reduction process where a predefined amount of oxidant is left reacting for a fixed time with the AuNRs. Both the oxidant amount and the reaction time must be re‐optimized for each AuNRs batch used as starting material, and this requires running several pilot reactions. In the end, even all these precautions do not fully grant reproducible results, as fluctuations of the reaction conditions can still influence the outcome, and the impossibility to quench the reaction prevents real‐time control.

To the best of our knowledge, the use of fast oxidants has not been considered so far. This is probably because it would further increase the sensitivity of the size tuning process to the reaction conditions. However, we reasoned that this issue could be addressed by using stepwise additions of small amounts of oxidant. In this way, the reaction would quickly reach completion after each addition, allowing the operator to safely monitor the reached position of the plasmon absorption and to decide whether to stop or to continue the process with further additions. In addition, since at each decision point the oxidant would be quantitatively consumed, one can expect that the colloids should remain unaltered during the purification process. The fast reaction kinetics of the oxidant should also, in theory, enable rapid quenching of the added reactant, allowing the tuning process to be forcefully stopped at any time. Remarkably, this protocol would converge to the desired outcome regardless of the characteristics of the starting batch, and this in turn would remove the need for pre‐optimization and pilot‐runs. The whole procedure, when combined with real‐time monitoring, could be easily automated, ultimately decoupling AuNRs synthesis from the operator's skills.

### 2.1 The Choice of the Oxidant

We screened potential oxidants against the following requirements: i) ready availability of the reagent; ii) fast reaction kinetics and “switchability” (intended as the possibility to quickly stop and eventually restart the oxidation reaction at will, to allow true real‐time control), iii) absence of transition metals which can form alloys with the gold nanostructures, as well as other species capable of strongly binding to Au surfaces poisoning them, iv) easiness of removal of the oxidation byproducts.

We found that iodine could fulfill all these requirements. It is widely available and inexpensive and can be quickly reduced with common, safe, and easy‐to‐use reductants such as bisulfite. It is known to be able to etch metallic gold [36, 37], and indeed the oxidation of AuNRs by iodine has already been reported for plasmonic sensing of iodine‐generating species [9, 10]. As AuNRs are synthesized in an aqueous environment, we decided to employ iodine in the form of the soluble triiodide ion.

### 2.2 The Rate of AuNR Oxidation by Triiodide

**FIGURE 1:**
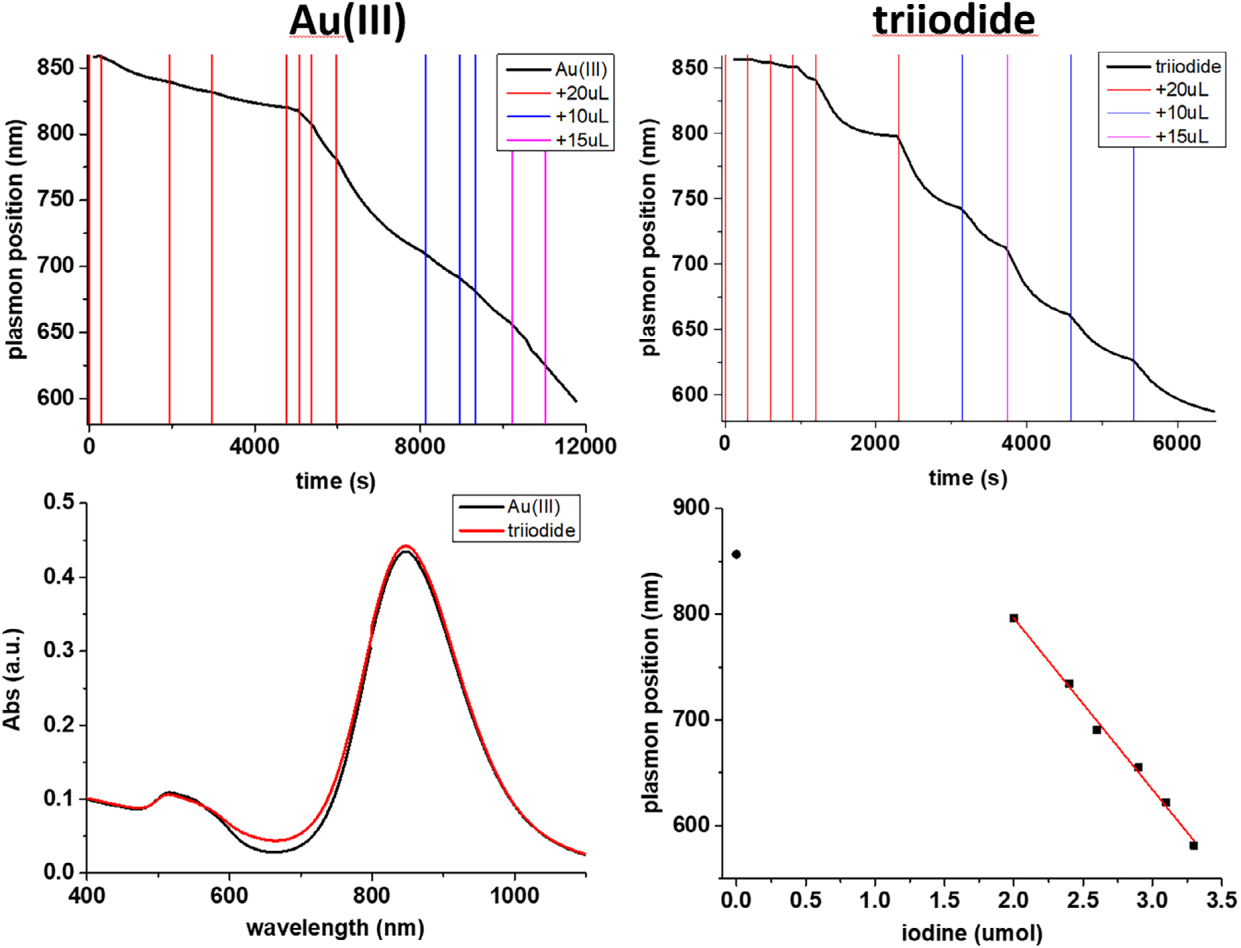
Above: evolution of the longitudinal plasmon position of an AuNRs suspension (10 mL, 500 µM in Au, 100 mM in CTABr) during oxidation by sequential additions (from 10 to 20 µL) of (left) tetrachloroauric acid (20 mM) or (right) triiodide (20 mM I_2_, I_2_/KI 1:5). Oxidant additions are evidenced by vertical lines. Below: UV‐NIR spectra of the starting AuNRs and relationship between the plasmon wavelength and the added amount of triiodide during the oxidation process (10 mL, 500 µM in Au). The “wasted” amount of triiodide consumed in the oxidation of leftover reductants was deduced from the difference between the intercept of the linear fit of the last 6 points (a slope of ‐162 nm/µmol I_3_^−^ was found) and the wavelength at the starting point, it resulted to be 1.63 µmol I_3_^−^ (Figure S1).

First, to ensure that iodine reactivity matches the requirements for a rapid and quantitative reaction, we run comparative oxidations of AuNRs by [AuBr_4_]^−^ and I_3_^−^. Small amounts of each oxidant were sequentially added (at time intervals of about 10 min) to an AuNRs dispersion (in the presence of CTAB 100 mM) under continuous monitoring of the UV‐NIR absorption spectrum (Figure 1). In both cases, we observed a blue shift of the absorption maximum of the longitudinal plasmonic band, diagnostic of the reduction of the aspect ratio of the AuNR. When Au(III) was used, the plasmon position vs. time plot showed a smooth profile, indicating a slow reaction rate, which was always far from reaching completion at the subsequent oxidant addition time. The absorption maximum took about 3 h to shift from 860 nm to 650 nm, and the reaction was still ongoing at that time. On the other hand, in the case of triiodide, the profile was clearly discontinuous and showed a sequence of rapid blue shifts followed by plateau regions, confirming that the reaction was always close to reaching completion when the subsequent oxidant aliquot was added. The greater reactivity of iodine with respect to Au(III) was further confirmed by the fact that, notwithstanding the presence of a series of plateau regions, it took slightly more than 1 h to shift the absorption maximum from 860 to 650 nm.

**FIGURE 2:**
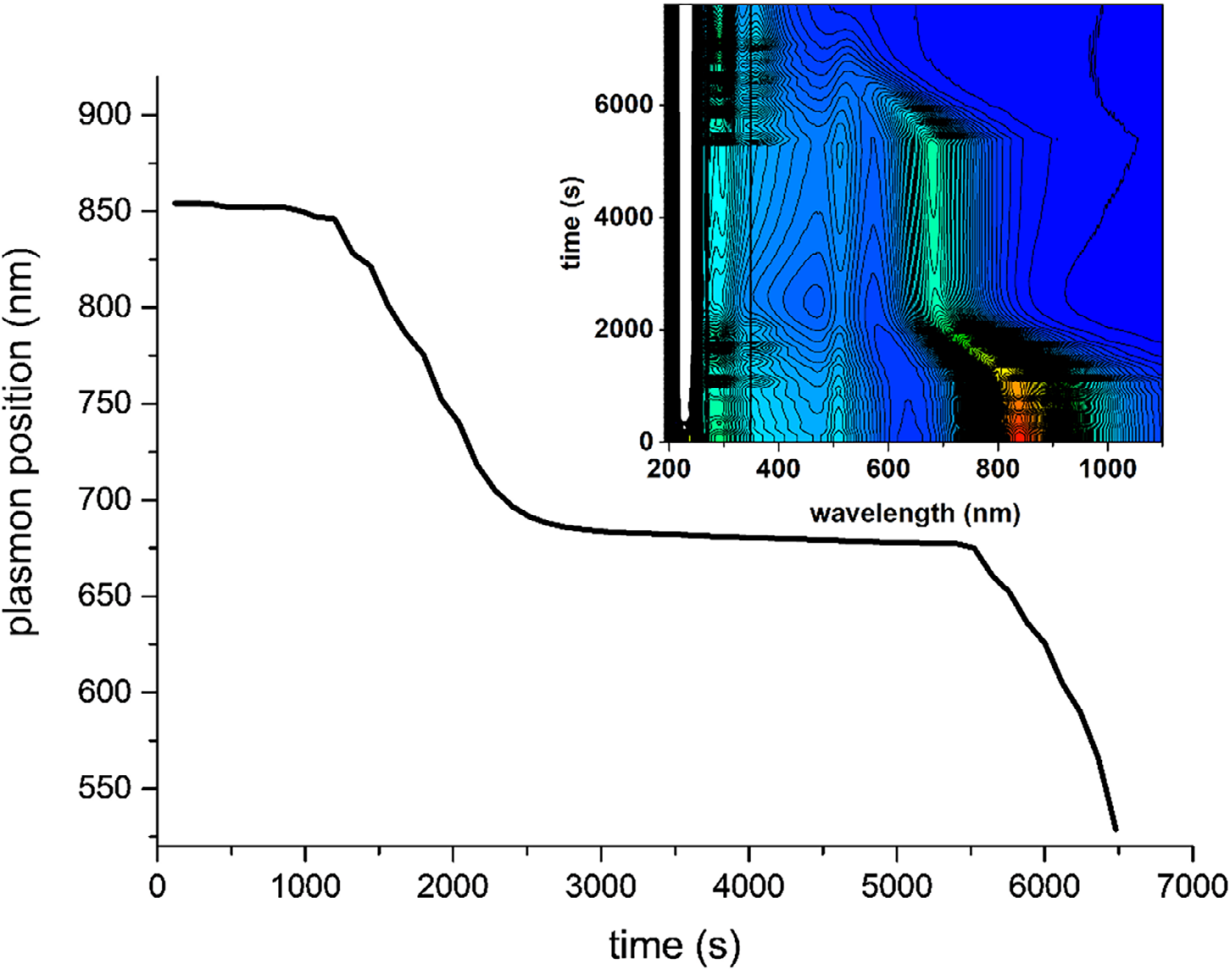
Evolution of the longitudinal plasmon position of an AuNRs suspension (10 mL, 500 µM in Au, 100 mM in CTABr) upon stopped and resumed oxidation with triiodide (20 mM I_2_, I_2_/KI 1:5). Oxidizer additions (from 10 to 20 µL) were stopped at around 2000 s and started again at around 5500 s. Inset: evolution of the UV‐NIR spectra in the 200–1100 nm region.

To investigate the stability of the etched AuNRs after the oxidant was consumed, we repeated the experiment with triiodide (Figure 2), but we stopped the additions when the plasmon band reached 685 nm. The position of the absorption maximum did not change any more after reaching the plateau position, demonstrating that the colloid remained stable, and no shape alteration processes occurred after reagent consumption. After 44 min, triiodide additions were started again, and the same behavior as in the first experiment was observed, that is, the stepwise shifts of the absorption maximum, showing that the oxidation reaction could be stopped and restarted at will without issues.

We then analyzed the extent of shift of the plasmon band as a function of the amount of oxidizer added (Figure 1, below). These two parameters turned out to be linearly related, as expected for a chemical etching process. The intercept of the data's linear fit lies at a wavelength much greater than the starting one, confirming that the first additions of oxidant did not produce any etching. This shows that parasite processes involving the oxidation of different species than metallic gold, such as leftover reductants from the AuNRs growth process, consumed most of the oxidant in the first stages of the reaction.

### 2.3 Quenching of the AuNRs Oxidation

The colloid stability reached after oxidation completion is in principle sufficient to grant the effectiveness of triiodide as an oxidant for the precise tuning of gold nanorods. Still, the possibility of quenching the etching reaction by the addition of a proper reagent would significantly improve the reliability of this synthetic strategy, thereby avoiding any reaction drift after the set endpoint is reached. Sodium metabisulfite was tested as a quencher since it fulfills the same requirements set for the oxidant selection. As iodine, sodium metabisulfite is cheap, safe, transition metal free, readily available, and its oxidized byproducts can be easily removed. Bisulfite ions can reduce triiodide via the following pathway [38]:


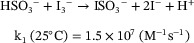




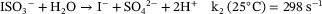




The quenching of both Au(III) and triiodide‐based oxidations was hence investigated by adding to the reaction mixture a large excess of metabisulfite (0.2 mmol, with respect to 4.4 µmol of Au(III) and 3.3 µmol of triiodide added to the sample) in a single portion.

**FIGURE 3:**
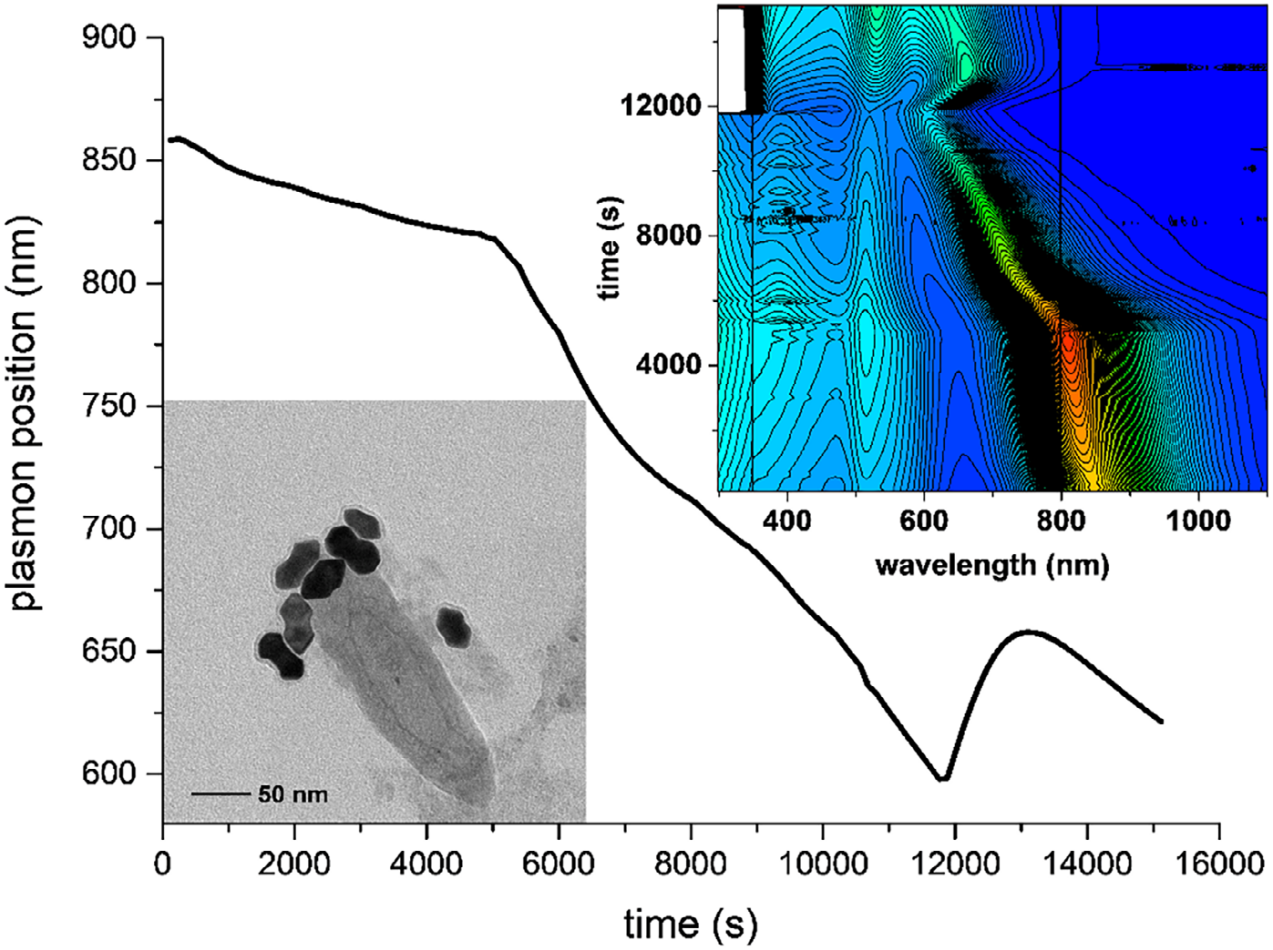
Evolution of the longitudinal plasmon position of an AuNRs suspension (10 mL, 500 µM in Au, 100 mM in CTABr) upon oxidation with HAuCl_4_ and quenching with a large excess of metabisulfite (at 11900 s). The slight increase in absorbance until 5000 s in the 2D plot (inset) is due to scattering caused by CTABr crystallization inside the system. In this case, before starting the oxidation, AuNRs were purified by centrifugation. Additions of tetrachloroauric acid (20 mM HAuCl_4_ and 100 mM in CTABr) are the same as marked in Figure 1. Insets: time evolution of the UV‐Vis spectra in the 200–1100 nm region and TEM image of the crystalline‐terminated dumbbell‐shaped structures obtained when an Au(III)‐based oxidation is quenched with metabisulfite.

In the case of Au(III)‐driven AuNRs etching, the addition of bisulfite resulted in a weird evolution of the absorption spectra (Figure 3), with the maximum of the longitudinal plasmon band initially shifting to longer wavelengths to subsequently invert direction and blueshift again (Figure 3, after quenching at about 11800 s). Such evolution suggests that the large amount of Au(I) and Au(III) species present in the mixture at metabisulfite addition time were quickly reduced to metallic gold and redeposited on the AuNRs. TEM analysis showed that this rapid growth was poorly anisotropic and led to the formation of dumbbell‐shaped nanoparticles with apparently crystalline terminations (Figure 3, inset).

**FIGURE 4:**
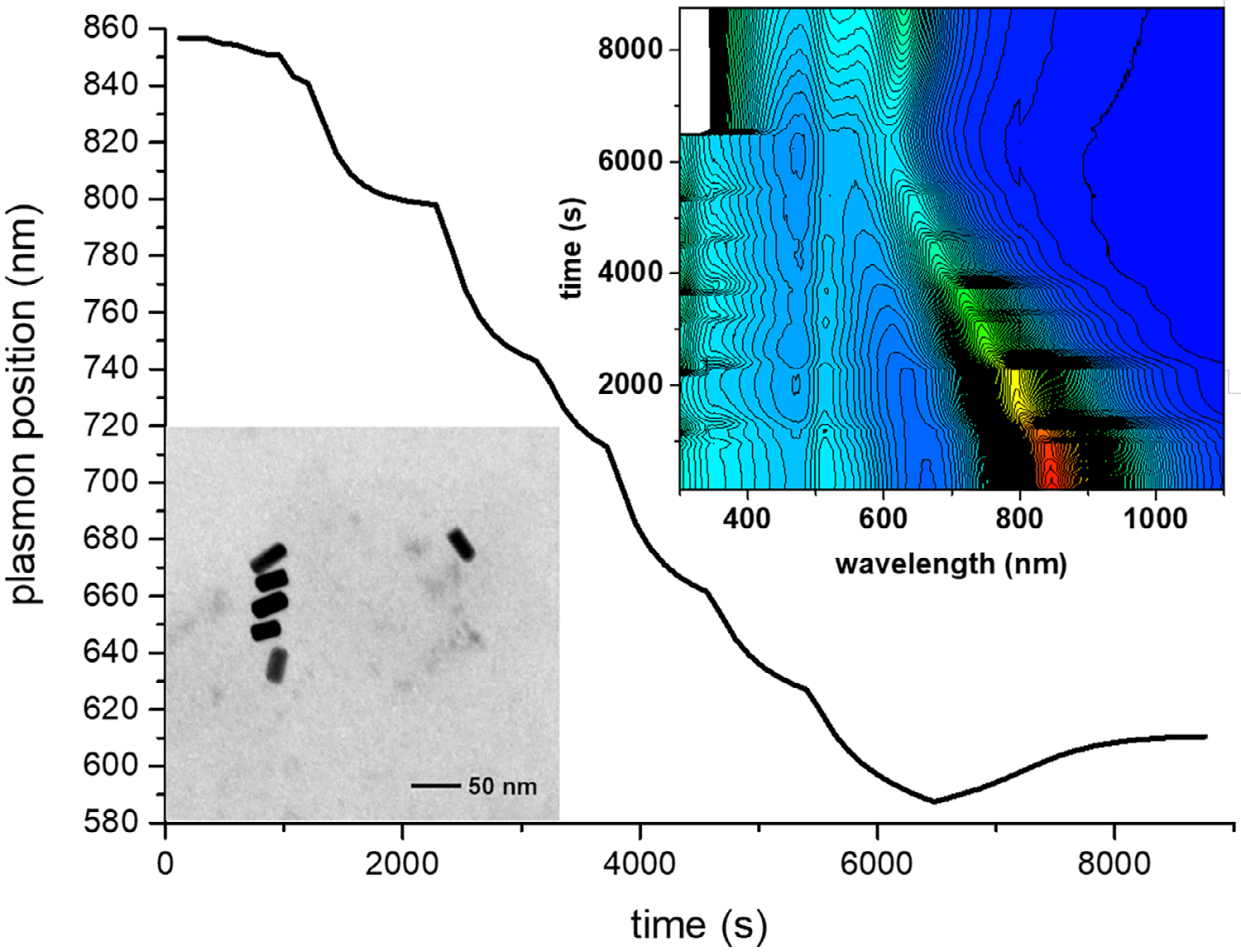
Evolution of the longitudinal plasmon position of an AuNRs suspension (10 mL, 500 µM in Au, 100 mM in CTABr) upon oxidation with triiodide and quenching with a large excess of metabisulfite. Quenching of triiodide was performed at 6200 s (200 µL metabisulfite 1 M). 215 µL of triiodide 20 mM (I_2_:KI 1:5) were added in total, additions are the same as marked in Figure 1. In this case, before starting the oxidation, AuNRs were purified by centrifugation. Insets: time evolution of the UV‐Vis spectra in the 200–1100 nm region and TEM image of the AuNRs.

By the contrary, in the case of triiodide‐driven oxidation, the addition of a large excess of bisulfite induced only a small red shift (Figure 4). This result can be explained by considering that a much smaller amount of gold ions was present in the reaction mixture, and consequently a limited regrowth occurred. Nicely, TEM analysis also showed that gold redeposition was more selective and retained the desired rod shape (Figure 4, inset).

**FIGURE 5:**
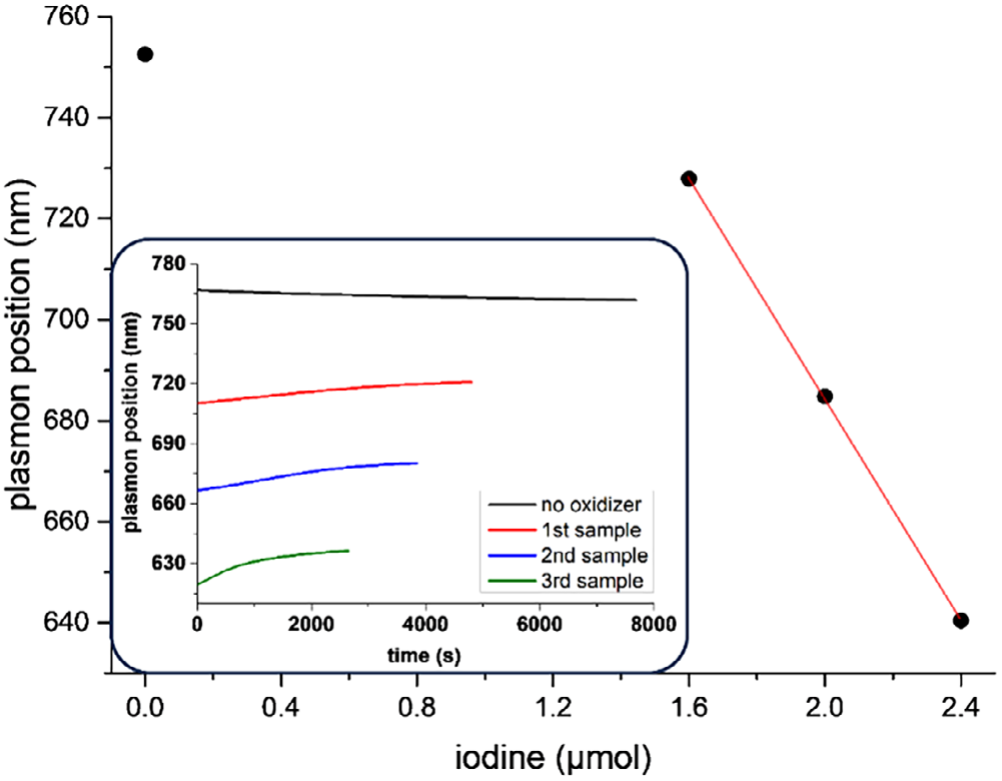
Relationship between the longitudinal plasmon position measured after oxidant quenching (with a large excess of reducing agent) and the total amount of added oxidant. The initial triiodide consumption is likely caused by oxidation processes involving leftover reductants from the AuNRs growth step. A slope of ‐109 nm / µmol I_3_^−^ was found for the linear part (see Figures S2 and S3). Inset: evolution of the longitudinal plasmon position after quenching with a large excess of metabisulfite (which causes Au redeposition and AuNRs regrowth). 200 µL of the reaction mixture (500 µM in Au and 100 mM in CTABr) sampled at different times during the oxidation process were diluted with 650 µL CTAB 100 mM and quenched with 50 µL metabisulfite 1 M.

We then studied the effect of quenching the reaction at different time points to provide more information on the whole process. Samples removed from the tuning reaction (performed by subsequent additions of oxidant at 10 min intervals) were quenched by addition of a large excess of metabisulfite, and their UV‐Vis spectra were measured to monitor the time evolution of the plasmon position (Figure 5). Also in this case, we found that the extent of redshift after quenching was linearly related to the total amount of triiodide added. The slope of the linear relation was smaller than that obtained in the case of the size tuning reaction performed without quenching (Figure S2), since in this case, part of the effects of oxidation are counterbalanced by the regrowth induced by metabisulfite.

It is worth noting, however, that the position of the longitudinal plasmon never returned to the original value after the addition of a large excess of metabisulfite, indicating that the regrowth process did not reconstitute the original rod's shape. Indeed, an increase in the intensity of the plasmon band was also observed during the regrowth. This suggested a pseudo‐isotropic deposition of gold on all the rod faces, with still a residual tip preference indicated by the red shift. The process might be favored by the adsorption of iodide ions onto the nanorod surface (Figures S5–S8) [39]. Additionally, the linear correlation between the longitudinal plasmon redshift and the amount of added oxidant (Figures 5, S2, S3) confirmed that the amount of gold redeposited on the rods was proportional to the amount of oxidant consumed in the oxidation reaction (and therefore to the total amount of gold etched).

**FIGURE 6:**
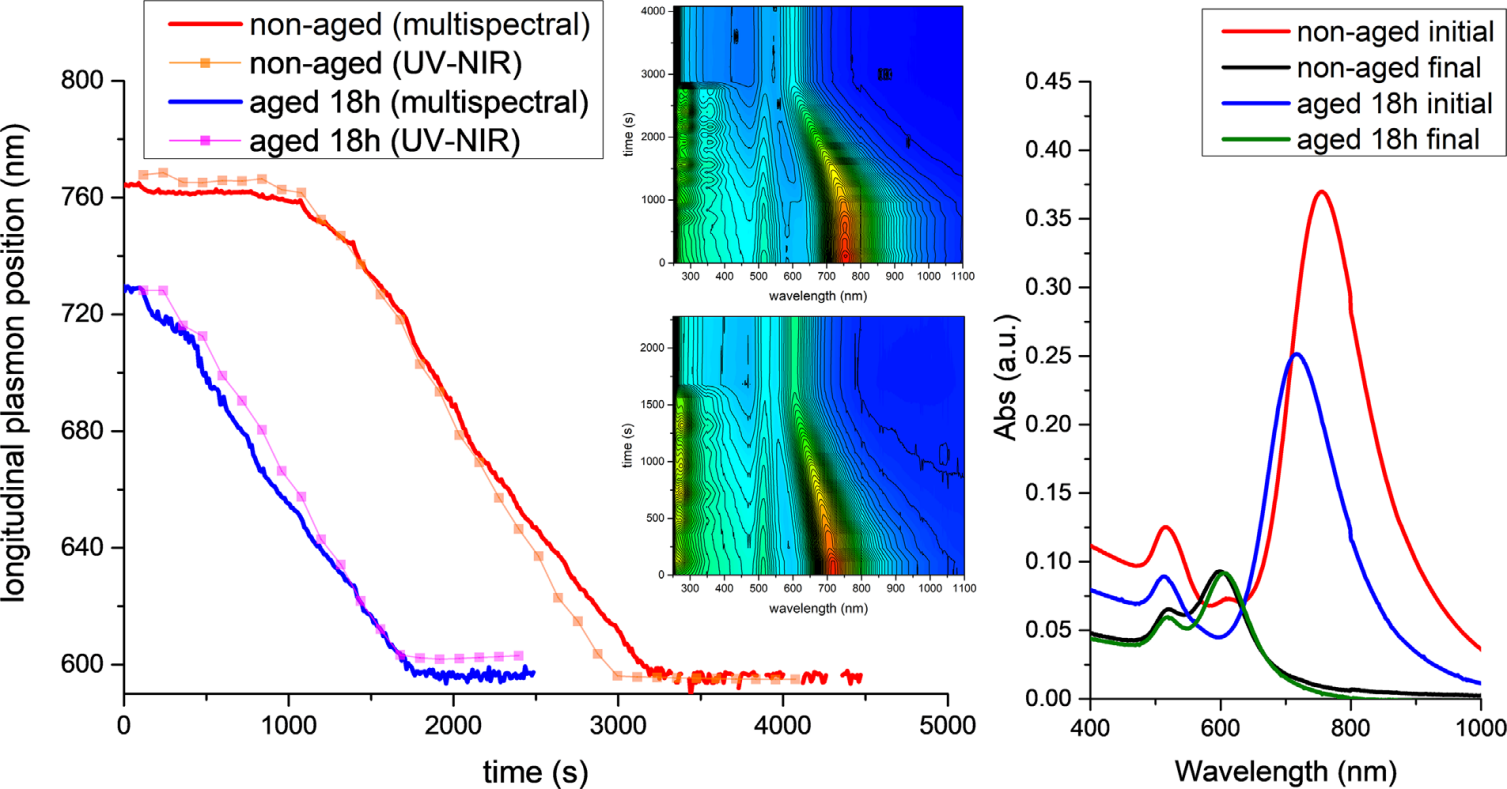
Oxidative tuning with triiodide performed by periodic additions of triiodide monitored by multispectral sensors (red and blue profiles), compared with parallel analysis with a commercial UV‐NIR spectrophotometer (orange and magenta profiles). The oxidation was performed by additions of triiodide (2 mM, 200 µL per addition) every 5 min. Final quenching was achieved by addition of a small excess of metabisulfite (2 mM, 200 µL). The total amount of oxidant and quencher solutions to be added was determined autonomously by the system (see infra). Notably, when quenching with a small excess of metabisulfite, the tuning process can be immediately stopped upon reaching the desired endpoint, without further evolution. In the red and orange profiles, the oxidation was performed immediately after the synthesis of the precursor AuNRs, while in the blue and violet ones, the oxidation was repeated on another AuNRs batch aged for 18 h under air exposure. Full Vis‐NIR spectra are shown on the right (the small imperfection at 800 nm is an instrumental artifact related to a change in the lamp source).

Since the reaction rate of bisulfite with triiodide is faster than that with Au(I) species, a simple adjustment of the amount of added bisulfite should allow to effectively stop the oxidation process with a negligible concomitant gold reduction and AuNRs regrowth. Accordingly, when we repeated the triiodide quenching with a reduced amount of bisulfite (0.8 µmol), the position of the longitudinal plasmon band remained constant (Figure 6).

To further assess the scope of this procedure and the mechanisms involved, we performed the size tuning process on two different samples of AuNRs synthesized independently and submitted to different aging. In the first case, we performed the oxidation and quenching protocol (Figure 6, orange) immediately after the AuNRs synthesis, while in the second (Figure 6, magenta), we stored the AuNRs for 18 h exposed to air under constant stirring before performing the oxidation. The two experiments required different amounts of triiodide and revealed relevant differences in the oxidation profiles. First, the starting wavelength of the longitudinal plasmon was different, suggesting that the two samples, albeit being prepared at a short distance with the same protocol, featured different aspect ratio and required different extent of etching. Second, while the oxidation performed on the “as synthesized” AuNRs showed the usual induction period, due to leftover reductants present in the sample, the oxidation performed on the “aged” sample did not show any induction, and the whole process required a significantly shorter time and reduced amount of oxidant to reach completion. This different behavior suggested that during the aging, the leftover reducing species remaining in the AuNRs sample were consumed, possibly by reacting with oxygen.

Remarkably, in both cases the final position of the longitudinal plasmon band was similar (595 and 603 nm, respectively), confirming that this protocol allows to obtain AuNRs with the desired aspect ratio notwithstanding the possible differences in the precursors used. Furthermore, no pilot‐runs or extensive AuNRs purification were necessary before the tuning.

### 2.4 Oxidation Selectivity

**FIGURE 7:**
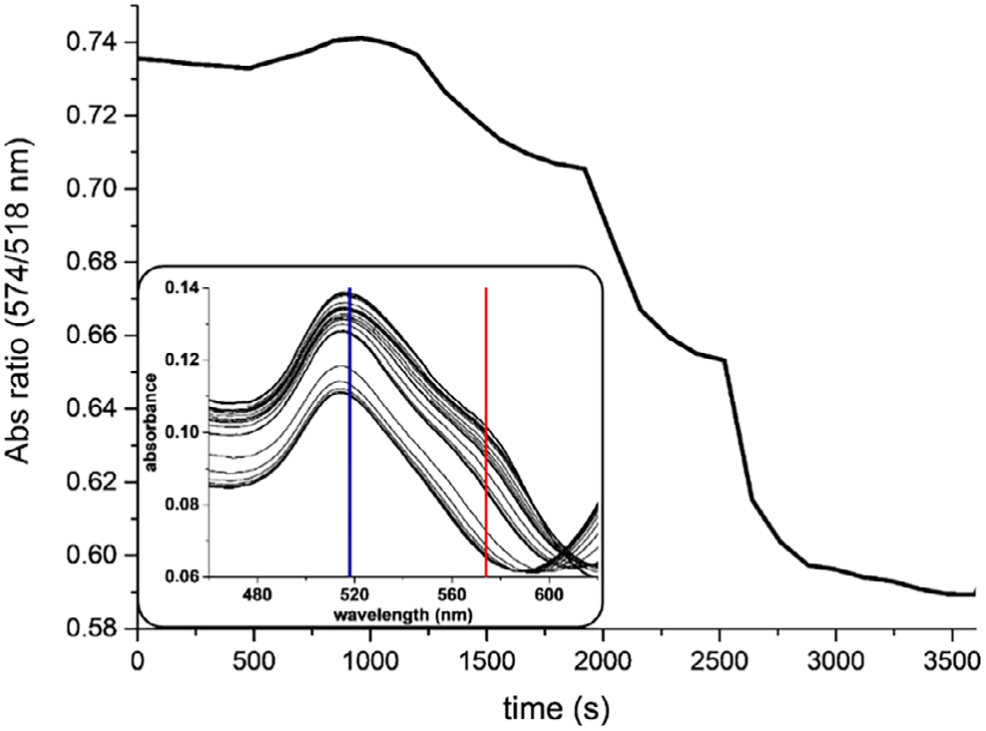
Ratio between the absorbance at 574 nm (nanogold byproducts) and that at 518 nm (AuNRs transversal plasmonic band) during the oxidation process with triiodide. Some UV‐NIR spectra of the reaction mixture are reported in the inset, with the two chosen wavelengths marked in red (byproducts) and in blue (AuNRs). Only a few spectra covering the first points are reported for clarity.

A careful inspection of the UV‐Vis spectrum of the as‐synthesized AuNRs batch showed the presence of an absorption shoulder around 574 nm, which quickly disappears after the initial oxidant additions (Figure 7). This shoulder is usually attributed to small gold nanostructures, such as gold nanocubes, which form as byproducts of the AuNRs synthesis and must be removed by centrifugation during the purification step [22, 40].

The disappearance of this shoulder during the etching process revealed another very relevant feature of this reaction: the selective dissolution of undesired small gold structures, which in principle allows performing the tuning process directly on a crude AuNRs dispersion, immediately after the growth step. This effect is quite relevant since not only could it remove the need for time‐consuming and labor‐intensive purifications [41], but it could also allow for turning the whole nanorod synthesis and size tuning into a one‐pot process (with the exception of the seeds formation).

### 2.5 The Complete Synthetic Process

**FIGURE 8:**
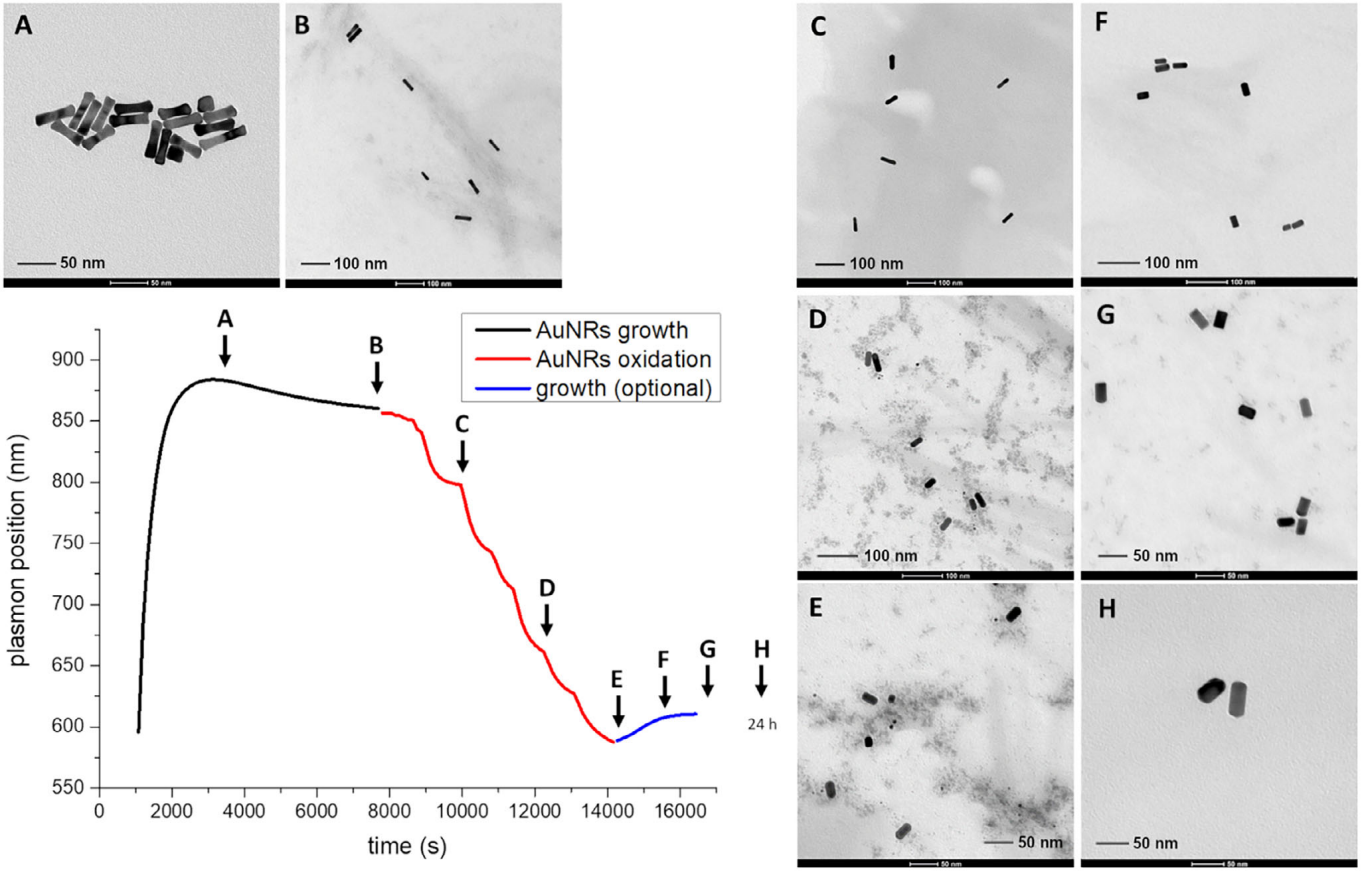
Complete AuNRs synthetic and tuning processes monitored by UV‐NIR spectroscopy by evaluating the AuNRs longitudinal plasmon position. The oxidative tuning with triiodide starts at point B and is quenched at point E (in this case with a large excess of metabisulfite). By employing a large excess of reducing agent, a second regrowth step (from point E to H) can be obtained. If a small excess is employed instead, the oxidation can be arrested (at point E) without further shape evolution. Sampling points (A‐H) for TEM analysis are marked with arrows. This whole process was performed manually, by only using a peristaltic pump to recirculate the reaction mixture inside a flow cell in the UV‐NIR spectrophotometer. Insets: TEM images of the AuNRs sampled at different times during the growth, oxidation, and (optional) regrowth stages (sampling points are marked with arrows). Debris present in some images is due to a quick purification step (via centrifugation) which evidently didn't completely remove the impurities. Note that scales are different through the images.

To test this hypothesis, we ran the whole synthetic procedure as a single process, starting from the seed injection in the AuNRs growth reaction mixture, following the variation of the longitudinal plasmonic band position (Figure 8 and Figures S9–S10). In addition, the forming nanostructures were sampled from the reaction vessel at different time intervals and subjected to TEM analysis to monitor their shape evolution.

Following the seeds’ injection in the growth solution, an initial fast redshift of the plasmon absorption maximum was observed (black portion of Figure 8). TEM analysis revealed that this was caused by the formation of dumbbell‐shaped structures (Figure 8A). Subsequently, the longitudinal plasmon underwent a small blue shift due to a surface reconstruction transforming the dumbbells into rods (Figure 8B) to eventually reach a stable plasmon position and shape. Subsequently, stepwise additions of triiodide were done directly in the reaction mixture. The longitudinal plasmon blue‐shifted (red portion of Figure 8), reaching the set wavelength. TEM images of samples taken during the oxidation phase (when the plasmon position reached 808, 675, and 600 nm respectively) clearly revealed a decrease of the AuNRs aspect ratio, as well as the quite homogeneous size dispersion reached (Figures 8C, D, E).

**FIGURE 9:**
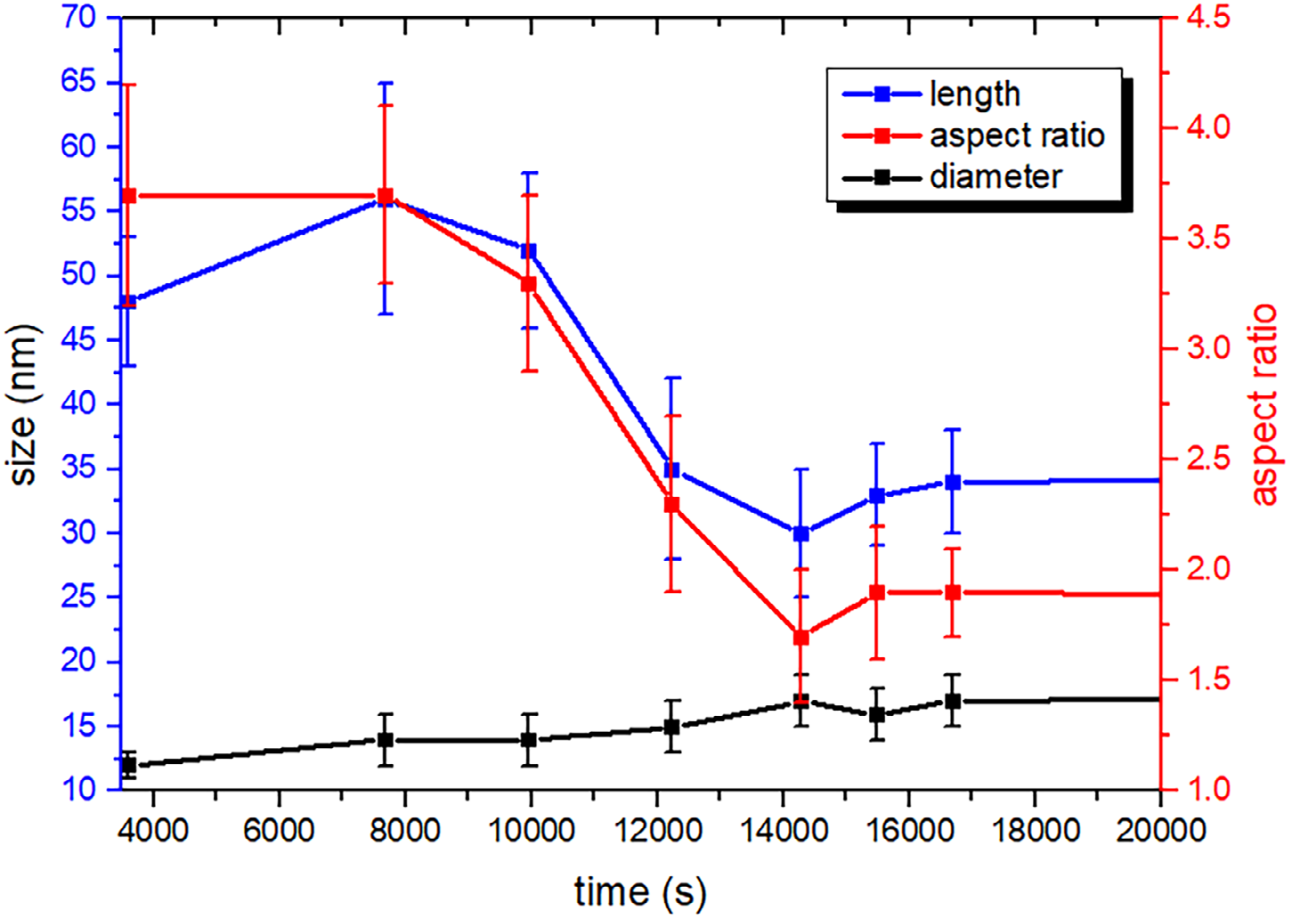
Relevant dimensions from TEM images: length, diameter, and aspect ratio of the nanorods (details on the size analysis can be found in the Supporting Information, Figures S16‐S39).

Having reached the set plasmon position (600 nm), the reaction was quenched with a large excess of metabisulfite (blue portion of Figure 8). As expected, the longitudinal plasmon band underwent a small red shift. TEM analysis of samples taken from the reaction mixture 20 min, 40 min, and 24 h after the addition of metabisulfite (Figures 8F, G, H) revealed the anisotropic growth of the rods, with the consequent increase of the aspect ratio. Figure 9 reports the evolution of the length, diameter, and aspect ratio of the AuNRs during the entire process. During the reconstruction phase following the initial fast growth, both AuNRs diameter and length slightly increased, quite in line with what can be expected for the transformation of dumbbells into rods. In the oxidation phase, the decrease of the relevant length corresponded to a further small increase in diameter. This revealed that the oxidation reaction did not cause only the anisotropic tips etching, but also an overall albeit modest reconstruction of the rods.

### 2.6 Automation of the Process

Having in our hands a one‐pot procedure for the size tuning of AuNRs allowing for on‐time monitoring and control, the logical follow‐up was the automation of the process. We hence designed a small modular robotic platform based on common commercially available hardware and 3D‐printed parts (Figure S11) featuring all the functionalities needed to autonomously perform AuNRs tuning. The system is composed of two syringe pumps (one for the oxidant and one for the quencher solutions), a peristaltic pump for the recirculation of the reaction mixture, and a Vis‐NIR multispectral photometric module to monitor the reaction. The photometric module is composed of two inexpensive multispectral sensors (ams AS7262 for the visible channels and AS7263 for the NIR ones) combined with 3D‐printed flow cells mounted on a moving part actuated by servomotors (this acts overall as a dual‐beam spectrophotometer for lamp emission correction). A 12 V incandescent lamp was chosen as the light source for the NIR module and a warm white 5 mm LED for the visible one. The position of the plasmonic band was calculated every 10 s by gaussian fitting of the multispectral sensors’ data. The estimated plasmon position was in good agreement with that measured with a commercial UV‐NIR spectrophotometer, with deviations of only 3–5 nm due to fitting errors (Figure S13). All the sensors and actuators were controlled by Arduino Nano microcontrollers, which were connected to a PC via serial port. A Visual Basic application was developed to elaborate user input, collect data, and manage the actuators through the microcontrollers.

This robotic device was able to monitor the AuNRs dispersion in real time and autonomously perform the tuning process, reaching the set endpoint. This was achieved by programming the device to wait after each oxidant addition for the completion of the etching reaction (by monitoring the slope of the longitudinal plasmon position vs. time profiles) before preceding to a subsequent addition. The amount of oxidant added was determined based on the degree of plasmon peak shift from the prior addition. The quencher was then added automatically, calibrated to prevent unwanted redshifting, once the plasmon band reached the target value. The employed operative algorithm is schematized in Figure S12.

To demonstrate the high precision and the consistence ensured by this novel automated tuning strategy, we repeated the size tuning of the two AuNRs batches described earlier, obtained by different synthesis and differently aged (Figure 6, red and blue; see also Figures S14, S15). In both cases, 600 nm was set as the requested final plasmon position.

The automated procedure could perform the size reduction without any preliminary experiment or calibration. Notably, in both cases, the tuning stopped exactly at the requested plasmon position (600 nm) according to the values obtained by the multispectral sensors. Reaction times and the amount of oxidant required were different but closely resembled those of the manual procedure. Eventually, after the quencher addition, a stable dispersion was obtained.

## 3 Conclusions

In conclusion, we demonstrated that the protocol here proposed allows the reliable aspect‐ratio fine‐tuning of gold nanorods. In contrast to the strategies so far proposed, this one enables the fast and predictable production of AuNRs with the desired plasmon band position irrespectively of the starting rods features. This result was achieved by using a fast oxidant which, in combination with real‐time monitoring, allows to quickly determine the effects of each single addition, to recalculate the quantities for the following one, and to quench the excess oxidant possibly added when the desired final point is reached.

In addition to being highly consistent, which is the main relevant value of this protocol, it avoids labor‐intensive daily “pilot‐runs” and pre‐purification steps. It also produces AuNR batches that remain stable even in the case of delayed final purification, allowing efficient operations programming and significant time savings.

Eventually, we demonstrated the possibility to fully automatize the process, enabling the continuous or on‐demand preparation of AuNRs having the exact required photochemical features.

## Conflicts of Interest

The authors declare no conflicts of interest.

## Supporting information



Experimental details, analysis of the regrowth process, details on process automation, TEM analyses of the AuNRs collected in the different experiments.
**Supporting File 1**: chem70699‐sup‐0001‐SuppMat.docx.

## Data Availability

The data that support the findings of this study are available from the corresponding author upon reasonable request.
