# Supplementary material for: Self‐Controlled Automated Strategy for the Synthesis of Gold Nanorods With Fine‐Tuned Longitudinal Absorption
Source: Chemistry. 2026 Jan 20;32(13):e02967. doi: 10.1002/chem.202502967 (PMC13047415; doi:10.1002/chem.202502967)
Supplement: Supplementary file 1 — Experimental details, analysis of the regrowth process, details on process automation, TEM analyses of the AuNRs collected in the different experiments. Supporting File 1: chem70699‐sup‐0001‐SuppMat.docx. [file CHEM-32-e02967-s001.docx]

**Supporting Information**

Self-controlled automated strategy for the synthesis of gold nanorods with fine-tuned longitudinal absorption

*Giordano Zanoni*, Elisabetta Collini, Fabrizio Mancin*

Department of Chemical Sciences, University of Padova, via Marzolo 1, 35131 Padova Italy.

**Contents:**

1. General……………………………………………………………………………………………………………………………… 2
2. Instruments and equipment……………………………………………………………………………………………… 2
3. AuNRs sampling………………………………………………………………………………………………………………… 2
4. Dependance between the plasmon blue-shift and the amount of oxidant added……………… 3
5. Pseudoanisotropic regrowth after quenching…………………………………………………………………… 5
6. Complete description of the synthetic process…………………………………………………………………… 9
7. Process automation…………………………………………………………………………………………………………… 11
8. AuNRs size analysis…………………………………………………………………………………………………………… 14
9. TEM images……………………………………………………………………………………………………………………… 22
10. **General**

All the chemicals used were reagent grade and used as obtained from the providers (Merck, TCI, VWR). Ultrapure water was produced with a Millipore Milli-Q Direct apparatus. All electronic components and mechanical hardware used in the construction of the devices were acquired from commercially available, general-purpose sources accessible to the public. Printed circuit boards and 3D-printed components were fabricated in-house by the author.

1. **Instruments and equipment.**

AuNRs purification was performed with an Eppendorf Minispin centrifuge and a Hettich UNIVERSAL 320. UV-NIR spectra were recorded with an Agilent Cary-5000 UV-NIR spectrophotometer using a quartz flow cell with a 1 mm pathlength. TEM images were recorded with a Jeol 300 PX electron microscope. AuNRs suspensions were pipetted on a sample grid, excess of liquid was removed with absorbent paper and the grids were dried by exposing them to open air.

1. **AuNRs sampling**

A 50 µL aliquot of the reaction mixture (500 µM in Au and 100 mM in CTABr) was diluted with 50 µL of a 10 mM CTABr solution, and the AuNRs were quickly isolated by centrifugation (15700 rpm for 3 minutes). The nanostructures were then purified by two cycles of redispersion in 50 µL of CTABr 10 mM and pelleting. The isolated AuNRs were eventually redispersed in 50 µL CTABr 10 mM and analyzed.

1. **Dependance between the plasmon blue-shift and the amount of oxidant added**


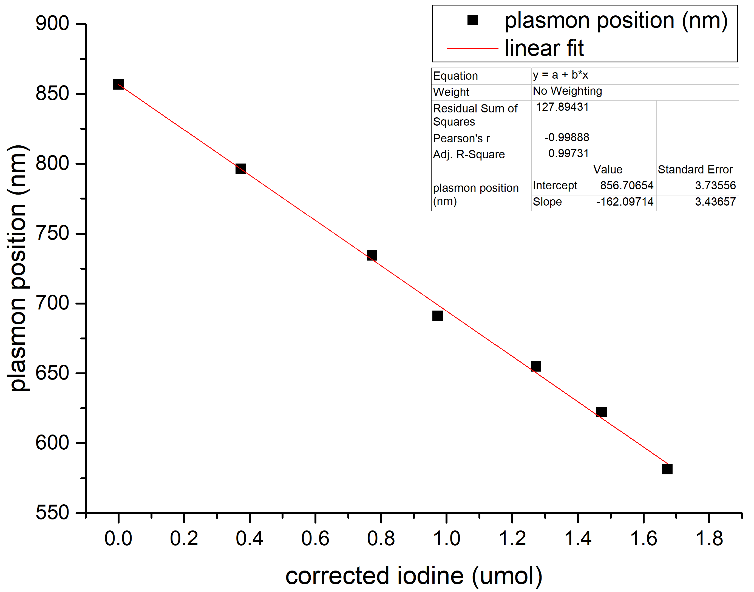


*Figure S1. Linear relationship between the longitudinal plasmon position and the “corrected amount” of triiodide added, i.e. the total amount of oxidant added less that reacted with the leftover reductants in the initial induction phase (when present).*


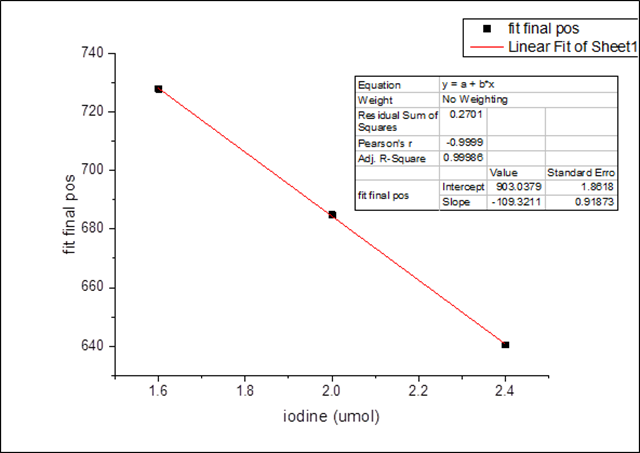


Figure S2. Linear relationship between plasmon position reached and added amount of oxidant after quenching with a large excess of metabisulfite (a slope of -109 nm/ µmol I_3_^-^ was found). The endpoint of each regrowth process (Figure 5) was estimated by fitting the time-dependent plasmon position profiles with a first order kinetic equation.

Figure S3. Linear relationship between plasmon position and total amount of oxidant added after quenching with a large excess of metabisulfite. The “wasted” amount of triiodide that is consumed in the oxidation of leftover reductants from the AuNRs growth step was calculated by linear fitting of the last 3 points (A slope of -109 nm/ µmol I_3_^-^ was found), see Figure S9. The projected point corresponding to the initial plasmon position (where no triiodide was added) indicated the amount of oxidant consumed in these side processes, this value was therefore subtracted from the amount of total added triiodide obtaining a “corrected triiodide” amount that indicates the actual amount of oxidant which reacted with the AuNRs.

1. **Pseudoanisotropic regrowth after quenching.**

**
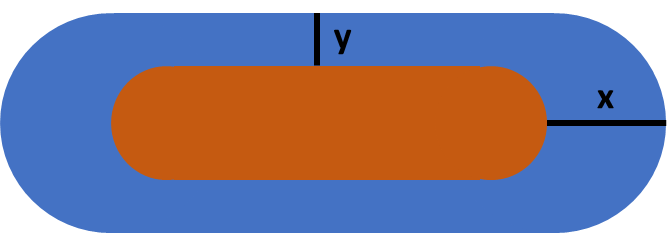
**

*Figure S4. Model of AuNR with spherical terminations used for the evaluation of the oxidizer-induced shortening and reductant-induced regrowth processes. Orange AuNR is grown to the blue one with an increase in length of 2x and an increase in diameter of 2y.*

To investigate the AuNRs regrowth process after the addition of a large excess of metabisulfite the following variables are defined:

V0 [= pi * r.^2*(l-2/3*r)] = initial volume of the AuNR

x=increase in length

y=radial increase

k=x/y

V1 = AuNR volume after growth

deltaAR = aspect ratio difference after growth

The following relationships can be established for different growth models:

**Pseudoanisotropic growth (AuNR with spherical ends) x = k*y:**

V1_pseudo = pi.*(-2./3.*x./k.^3+2.*x./k.^2.*(1+k.*x./k-r)+2.*x./k.*(-r.^2+l.*r+2.*r.*k.*x./k));

y = x/k;

deltaV_pseudo = V1_pseudo - V0;

deltaAR_pseudo = (l+2.*k.*x./k)./(2.*(r+x./k))-l./(2.*r);

**Isotropic growth (AuNR with spherical end) x = y:**

V1_iso = pi*(4/3*x.^3 + x.^2*(l+8/3*r)+x*2*r*l+r.^2*l-2/3*r.^3);

deltaV_iso = V1_iso - V0;

deltaAR_iso = (l +2.*x)./(2.*(r+x))-l./(2.*r);

**Anisotropic growth (AuNR with spherical end) x = 0:**

V1_aniso = pi*r^2*(l+x-2/3*r);

deltaV_aniso = V1_aniso - V0;

deltaAR_aniso = x/( 2*r);

The variation in the longitudinal plasmon position is assumed to be linearly related to the aspect-ratio variation of the AuNRs. The relative variation of the plasmon position for the oxidizer-induced shortening and reductant-induced regrowth is calculated (assuming both in the growth sense) using the average dimensions obtained from TEM analysis:

dPlasmon_oxid = (plasmon(B)-plasmon(E))/plasmon(E) = 0.4798

dPlasmon_quench = (plasmon(H)-plasmon(E))/plasmon(E) = 0.07129

Then using the above formula for anisotropic growth it was calculated the expected aspect-ratio variation from sample E (oxidized) to sample B (precursor) assuming the radius remaining constant at 8.5 nm and the length to grow to 26 nm (x = 13 nm).


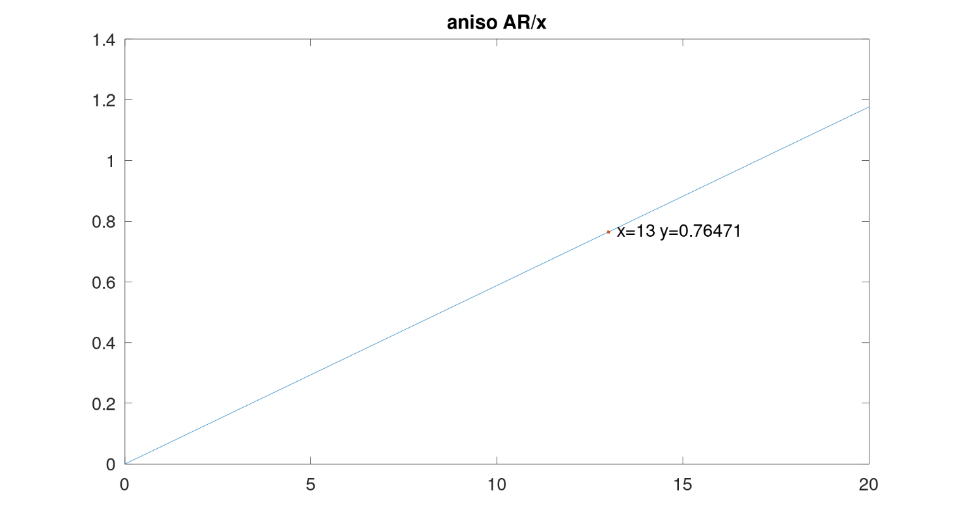


*Figure S5. Calculation of the predicted aspect-ratio variation for the oxidation process (seen backward as anisotropic growth). Diameter is kept constant at 17 nm and a growth of 26 nm (x = 13 nm) is used, as measured form TEM analysis. pAR_oxid = 0.7647*

Predicted aspect-ratio variation: pAR_oxid = 0.7647

The aspect-ratio variation during the reductant-induced regrowth (dAR_regrowth) was calculated from pAR_oxid, the relative variation of the plasmon position during regrowth (dPlasmon_quench) and that during oxidation (dPlasmon_oxid):

dAR_regrowth = pAR_oxid *dPlasmon_quench/dPlasmon_oxid = 0.7647*0.07129/0.4798 = 0.1136

Then introducing this value in the pseudoisotropic growth formula above, the ratio (predicted_k) between the variation in AuNR length and the variation in AuNR diameter for the regrowth step was calculated:


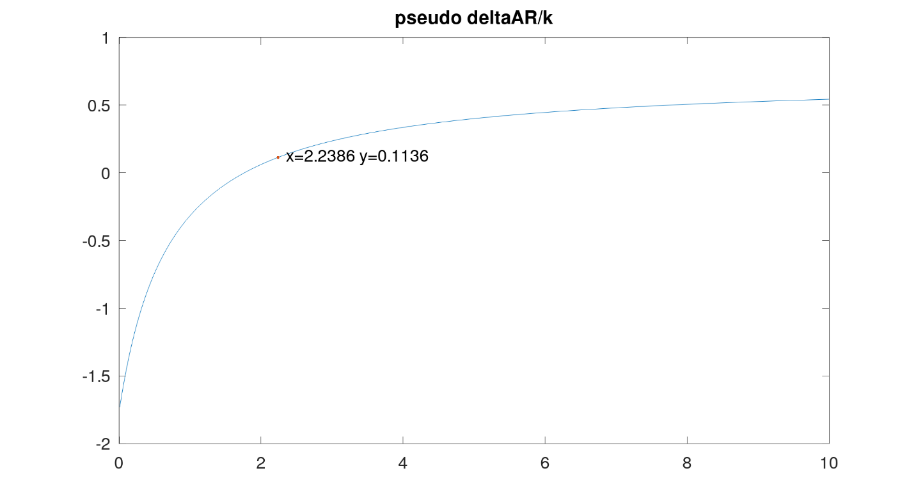


*Figure S6. Calculation of the predicted aspect ratio between growth in length and growth in diameter with a pseudoanisotropic growth model. predicted_k (x/y) = 2.239*

predicted_k = 2.239

A value of k can be measured From TEM analysis (measured_k):

measured_k = (length(H)-length(E))/(diameter(H)-diameter(E)) = 3

By calculating the average AuNR volume of sample B, E and H from TEM data (figure S7), the volume variation of the oxidation and regrowth processes can be compared (figure S8). These values are similar enough to suggest a complete redeposition of all the gold etched during the oxidative tuning process.

This is also supported by the linear correlation between the amount of oxidizer added and the longitudinal plasmon position measured after quenching with a large excess of metabisulfite.

Another evidence is provided by the absolute value of the slope (-109.3 nm/µmol I_3_^-^) which is lower than that obtained when regrowth is not performed and the oxidation is just quenched (-162 nm/µmol I_3_^-^), see figures S9 and S10.


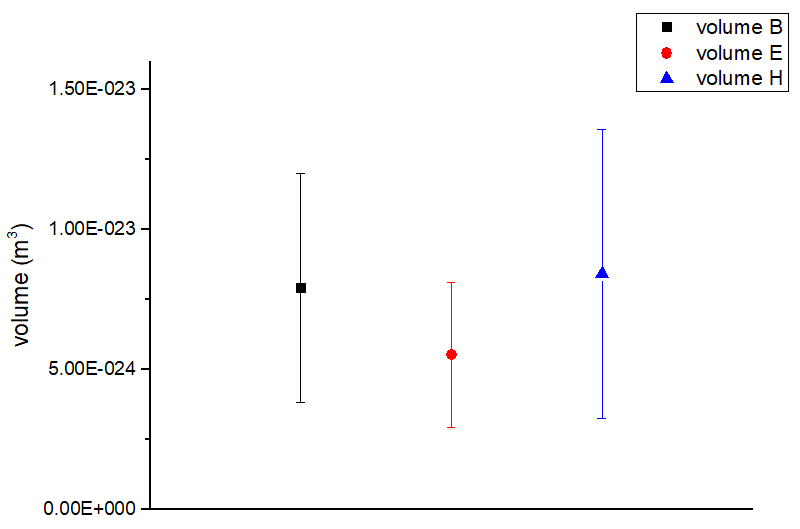


*Figure S7. Average AuNR volume in sample B (precursor), E (after oxidation) and H (after regrowth).*


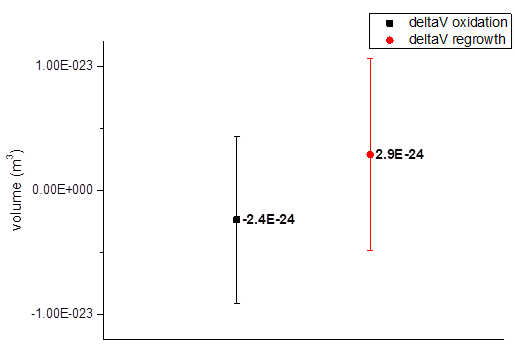


*Figure S8. AuNR volume variation during the oxidation and regrowth processes.*

1. **Complete description of the synthetic process.**


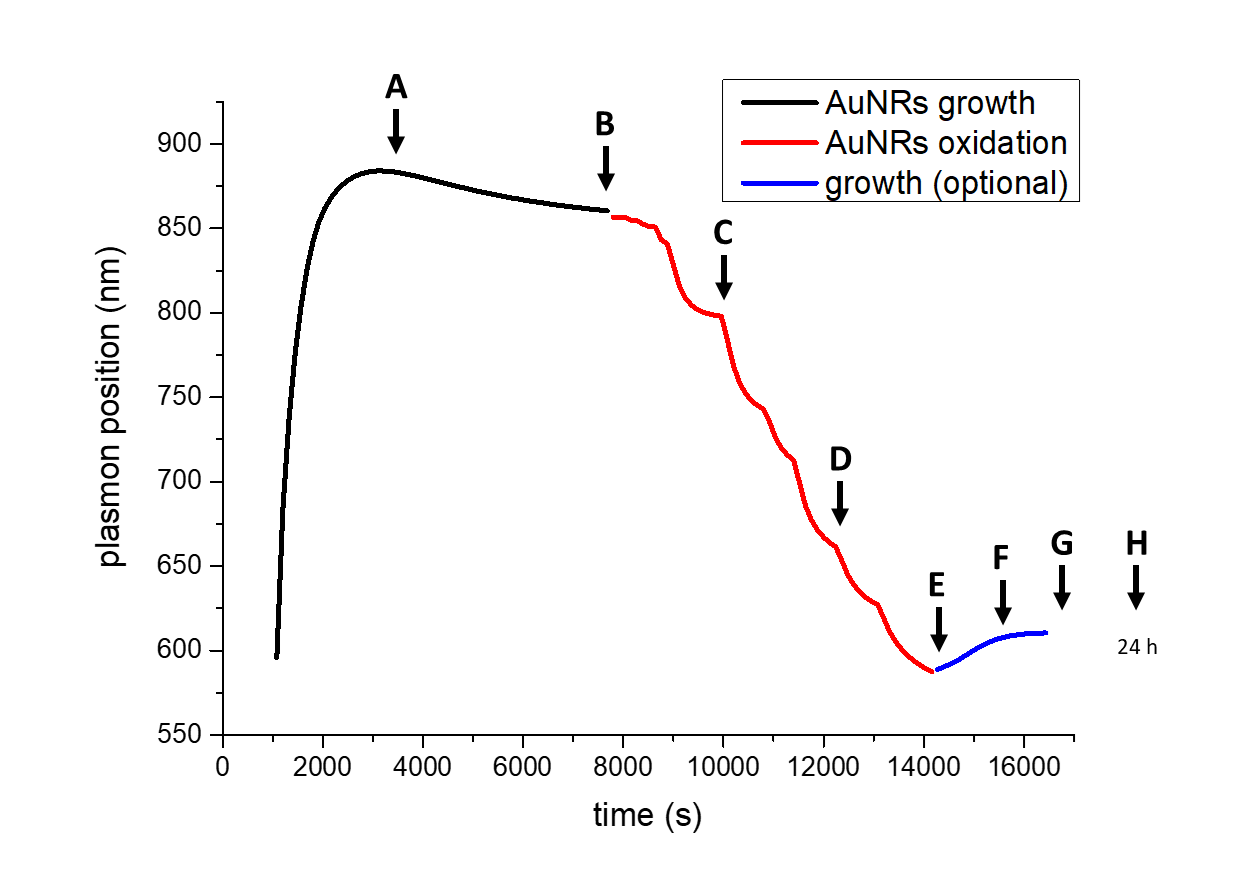


*Figure S9. Evolution of the longitudinal plasmon band position during the whole AuNRs synthesis and tuning processes. Sampling times (samples A-H) for TEM analysis are marked with black arrows. This whole process was manually performed, only a peristaltic pump recirculating the mixture inside a flowcell in a commercial UV-NIR spectrophotometer was employed.*

**Step 1 – gold seeds synthesis.** In a 20 mL scintillation vial, 4.9 µL of a 254 mM HAuCl_4_ solution were dissolved in 4.7 mL of a 100 mM CTABr solution at 30°C under vigorous stirring, 300 µL of a 10 mM NaBH_4_ solution were then quickly added. The so formed brown suspension of seeds (non-plasmonic small gold clusters) was left under stirring for 5 min.

Step 2 – AuNRs growth. (black curve in Figure S1) 19.7 µL of a 254 mM HAuCl4 solution and 190 µL of a 1 M HCl solution were added to 10 mL of a 100 mM CTAB solution under vigorous stirring at 30°C. Dissolution of the gold precursor takes some time. Then 120 µL of a 10 mM AgNO3 solution and 100 µL of a 100 mM ascorbic acid solution were added followed by 24 µL of the seeds dispersion prepared in step 1. The reaction mixture was then kept under stirring at 30°C for at least 2.5 hours (the mixture can be also conveniently left under stirring overnight).

Step 3 – AuNRs oxidation. (red curve in Figure S1) A I2:KI 1:5 solution (2 mM in I2) was added to the AuNRs mixture obtained in Step 2 in 200 µL portions at 5-minutes intervals until the desired plasmon position was reached. Conveniently, the amount of oxidant added, and the time delay between additions, could be scaled based on the effects that the previous addition had on the longitudinal plasmon position. This allowed us to reach precisely the desired final point. Once the final point was reached, the reaction was quenched by the addition of sodium metabisulfite (250 µL of a 2 mM solution, or an amount scaled to be in slight excess with respect to the last triiodide addition performed). The mixture was then centrifuged at 12000 rpm for 8 min to isolate the gold nanorods, which were then purified with three cycles of redispersion in 10 mL CTABr 100 mM and centrifugation. The final AuNRs can be conveniently stored dispersed in CTABr at least 10 mM. Note that complete CTAB removal must be avoided to prevent nanorods aggregation.

Step 4 – optional growth. (blue curve in Figure S1) This step is not part of the proposed AuNRs tuning process, but it can be performed if there is interest in having gold redeposition on the obtained AuNRs, for example to get higher extinction coefficients or bigger nanostructures. A large excess of metabisulfite is added to the obtained gold nanorods (for example 250 µL of a 20 mM solution) to induce gold redeposition. Once equilibrium is reached and no further evolution is observed, the AuNRs can be isolated and purified as described in step 3.


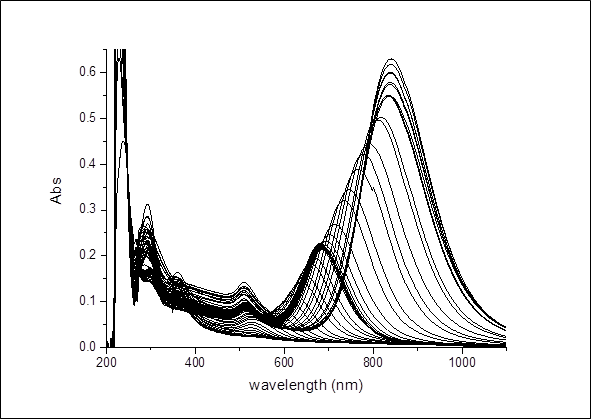


*Figure S10. The complete gold nanorod oxidation process with triiodide followed by UV-NIR spectrophoscopy, the whole UV-NIR spectra are shown. The small imperfection visible at 800 nm is caused by a small instrumental delay in the spectrum acquisition.*

1. **Process automation**


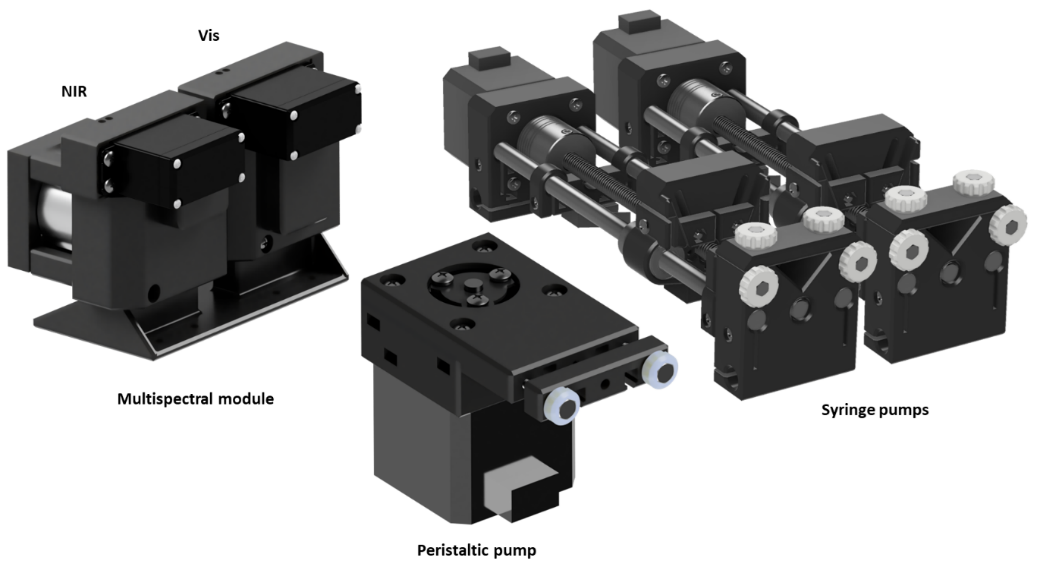


Figure S11. Homebuilt modules composing the robotic AuNRs tuner. Syringe pump modules are open source and have been made available for public access on the author's GitHub repository (G.Zanoni, Modular-Syringe-Pump-v.1, 2025, <https://github.com/giordanozanoni/Modular-Syringe-Pump-v.1>), other modules will likely be released after further modifications and refinement.


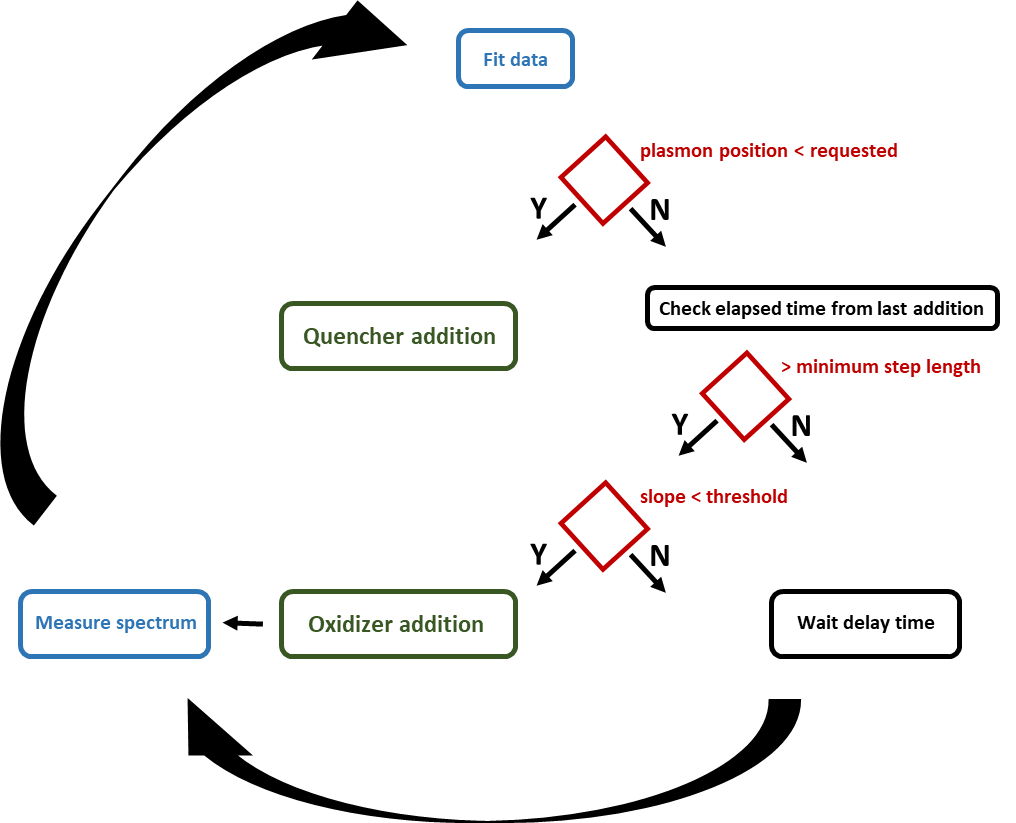


Figure S12. Scheme of the (unoptimized) operative algorithm employed in the automatic AuNRs tuning. Minimum step length was set to 5 min. Slope treshold was set to 3 nm/min. Delay time was 10 s.


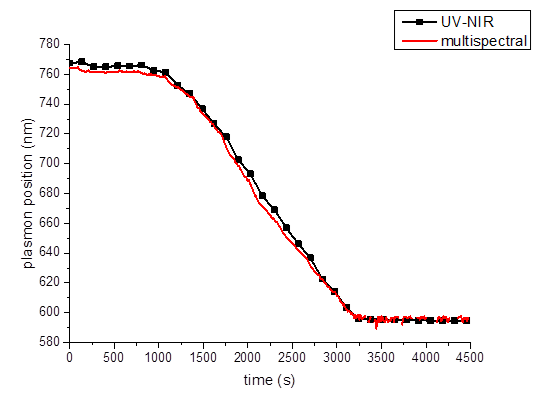


*Figure S13. Comparison between the longitudinal plasmon position obtained with the commercial UV-NIR spectrophotometer and that obtained by fitting the data from the multispectral sensors used by the robotic platform. Small offsets can be present due to fitting errors but, overall, the sensors turned out to be more than suitable for demonstrative purposes.*


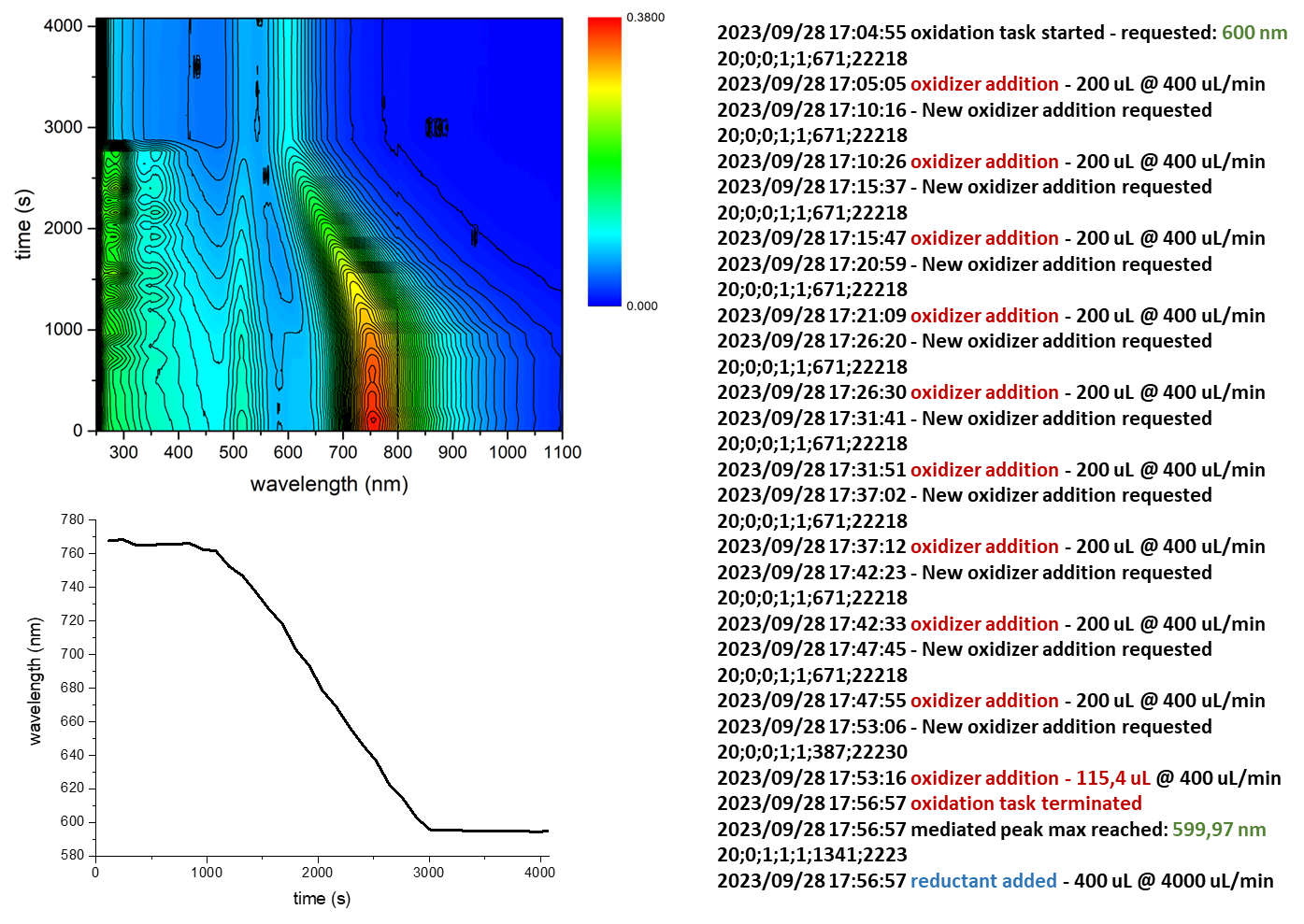


*Figure S14. Example of fully automated AuNRs tuning performed by the robotic system. Top left: 2D plot of UV-NIR spectra recorded with a commercial instrument during the tuning process; bottom left: longitudinal plasmon position vs time plot; right: log provided by the robot. The initial longitudinal plasmon position is at 767 nm, the requested final position is 600 nm.*


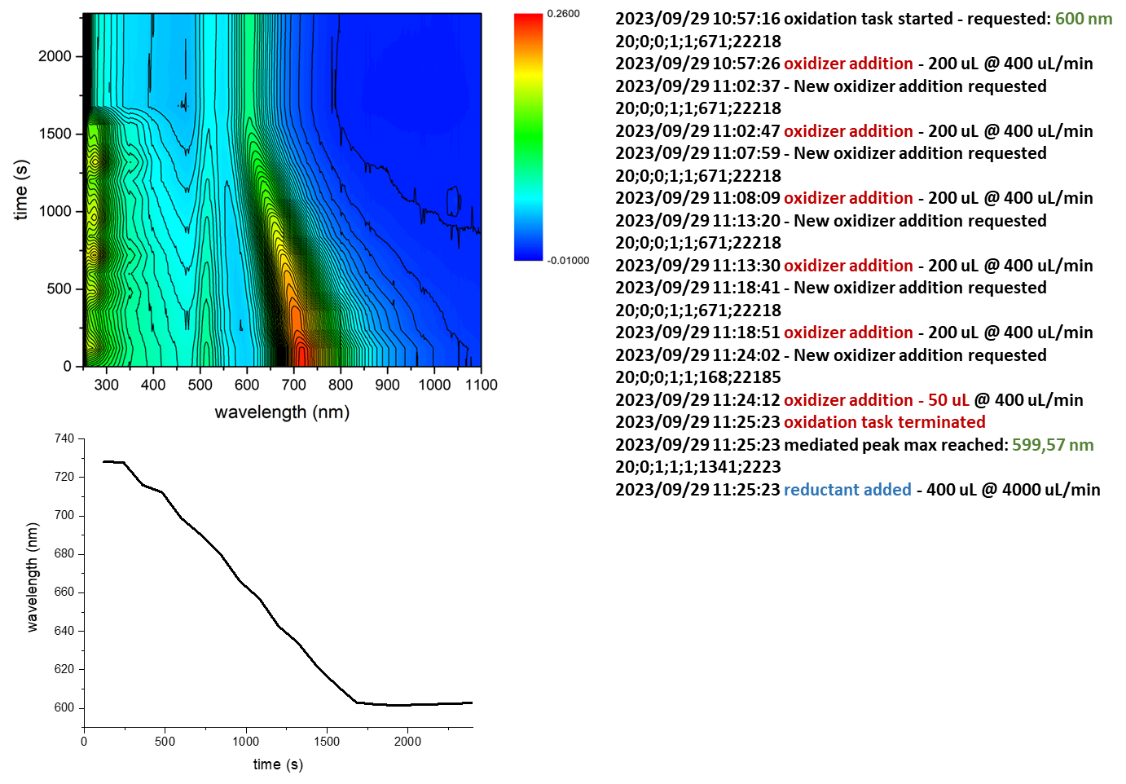


*Figure S15. A second example of fully automated AuNRs tuning performed by the robotic system. Top left: 2D plot of UV-NIR spectroscopy during the tuning process; bottom left: longitudinal plasmon position vs time plot; on the right is the log provided by the robot. The precursor AuNRs were left 18 h at room temperature exposed to open air before performing this tuning. The initial longitudinal plasmon position is 728 nm and the requested final position is again 600 nm.*

1. **AuNRs size analysis (ImageJ software image analysis, gaussian fittings performed with Origin Pro 9.1):**


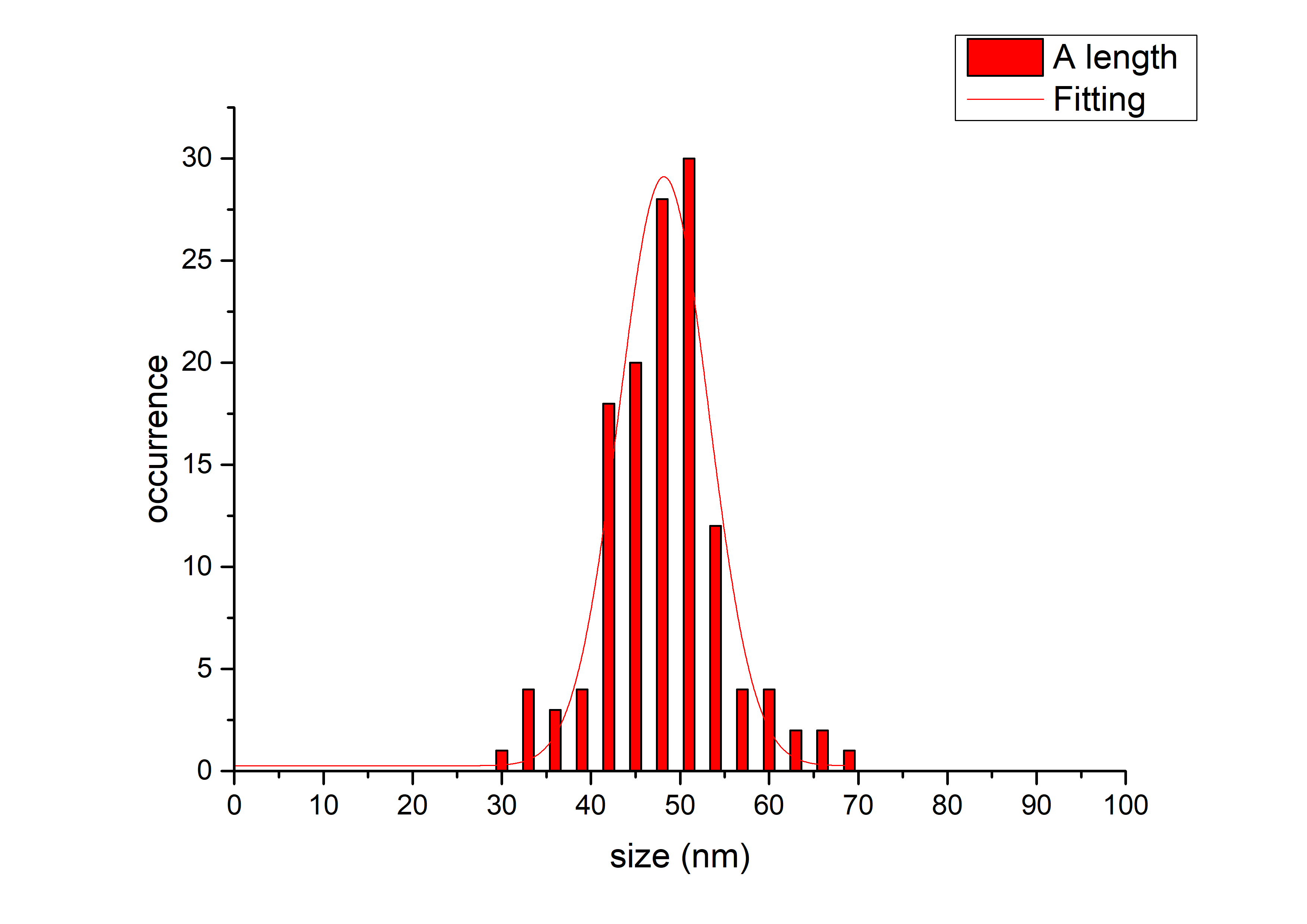


Figure S16. Sample A, length = 48 ± 5 nm


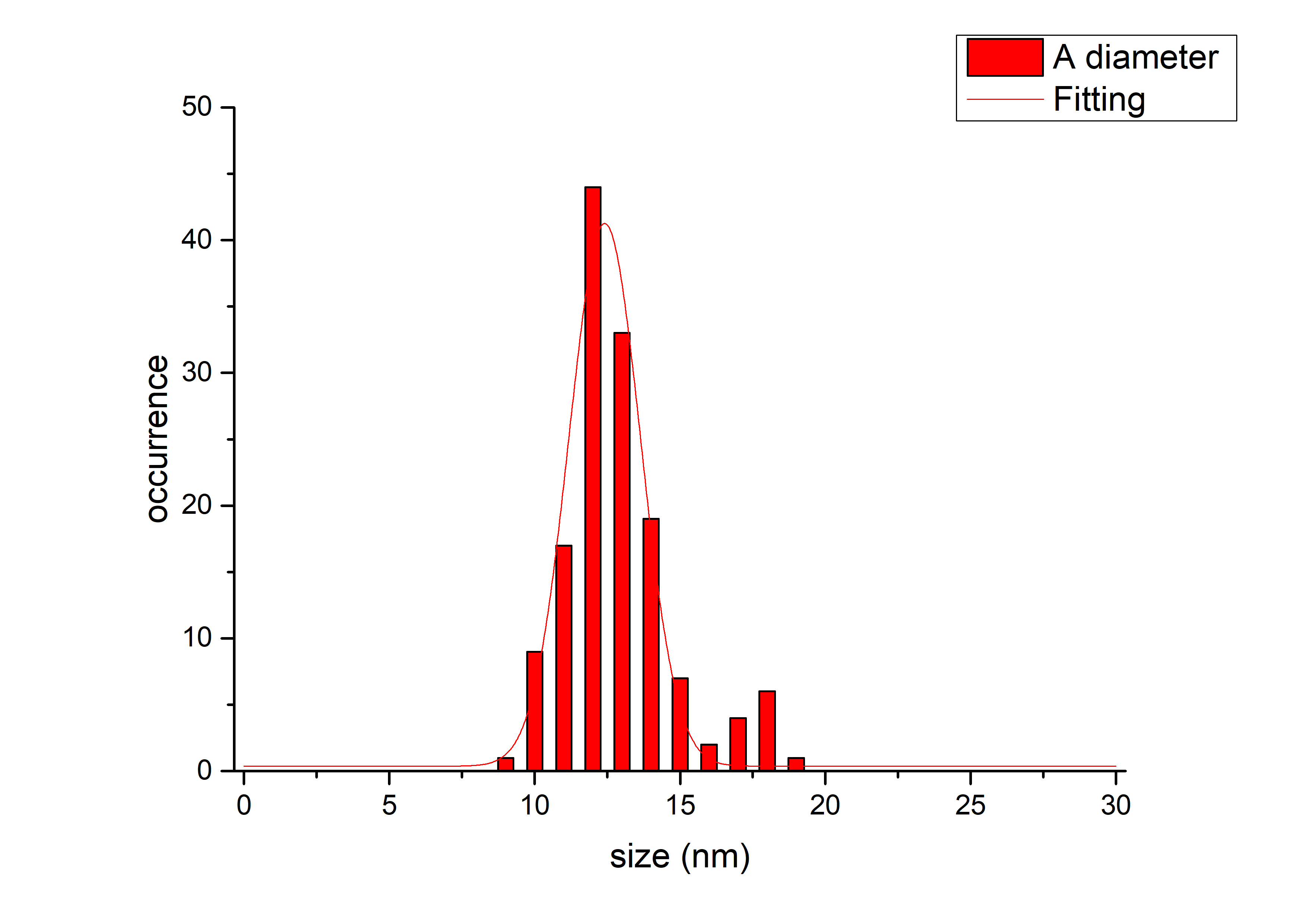


Figure S17. sample B, diameter (middle of the dumbell) = 12 ± 1 nm


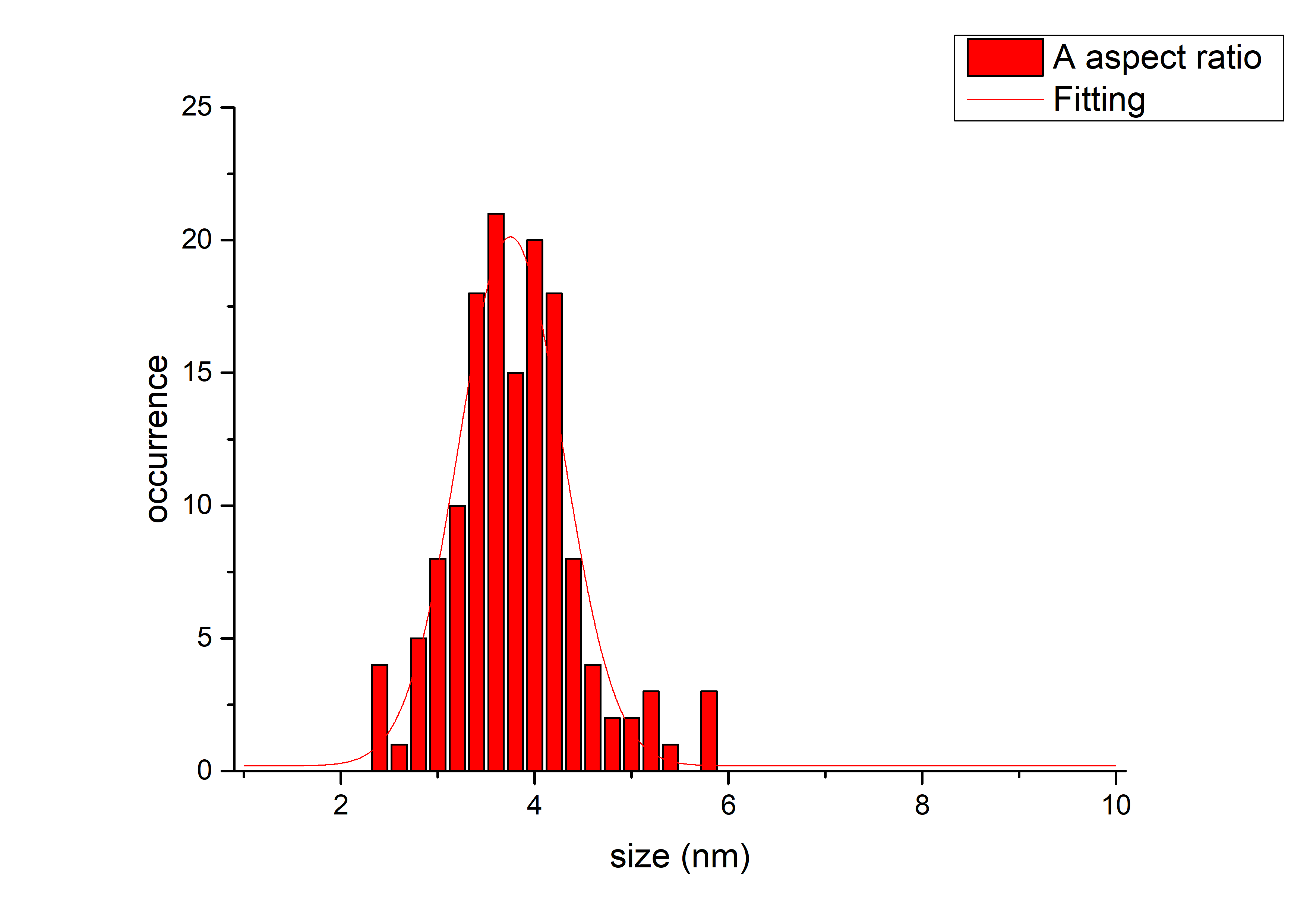


Figure S18. Sample A, aspect ratio = 3.7 ± 0.5 nm


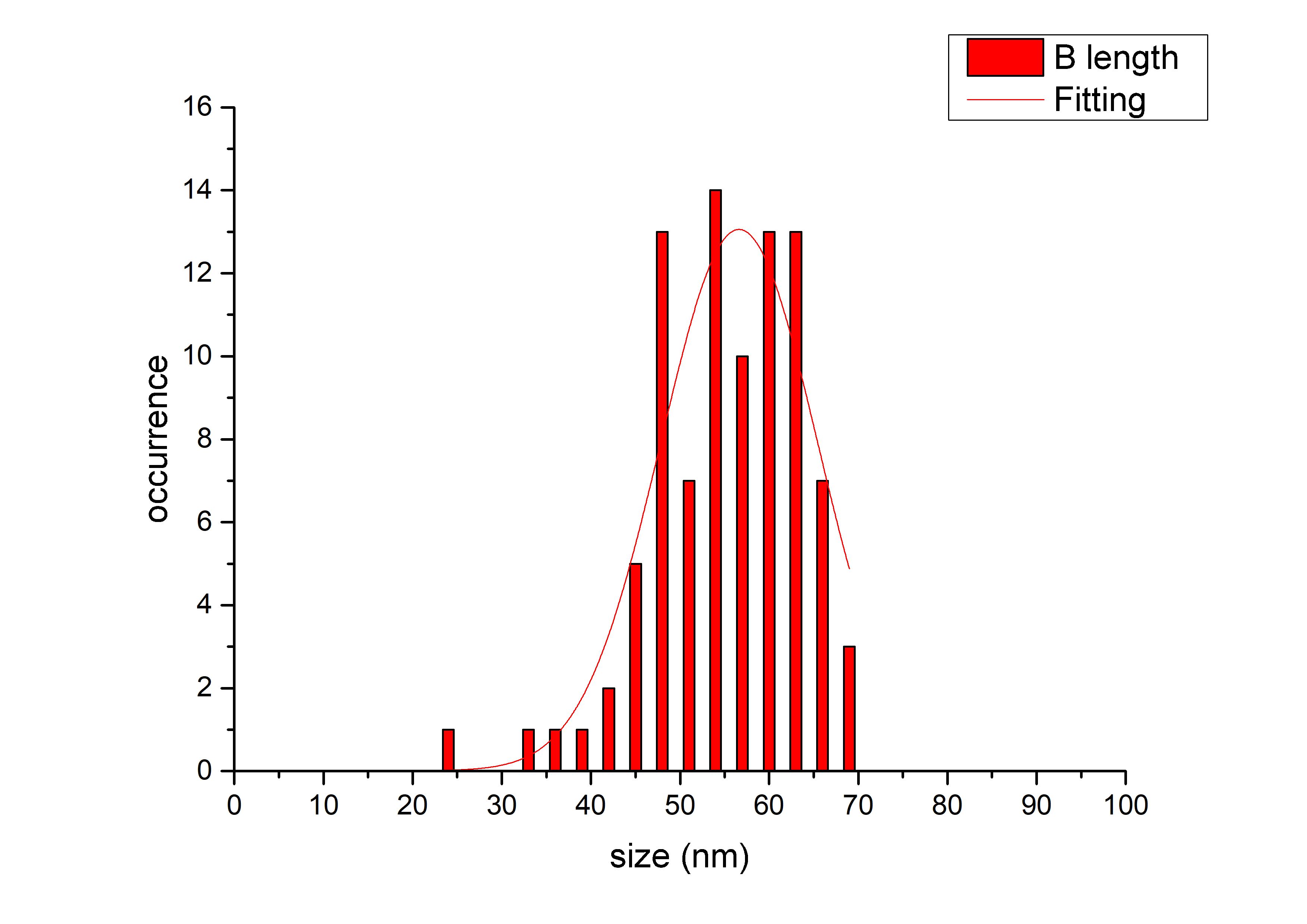


Figure S19. Sample B, length = 59 ± 9 nm


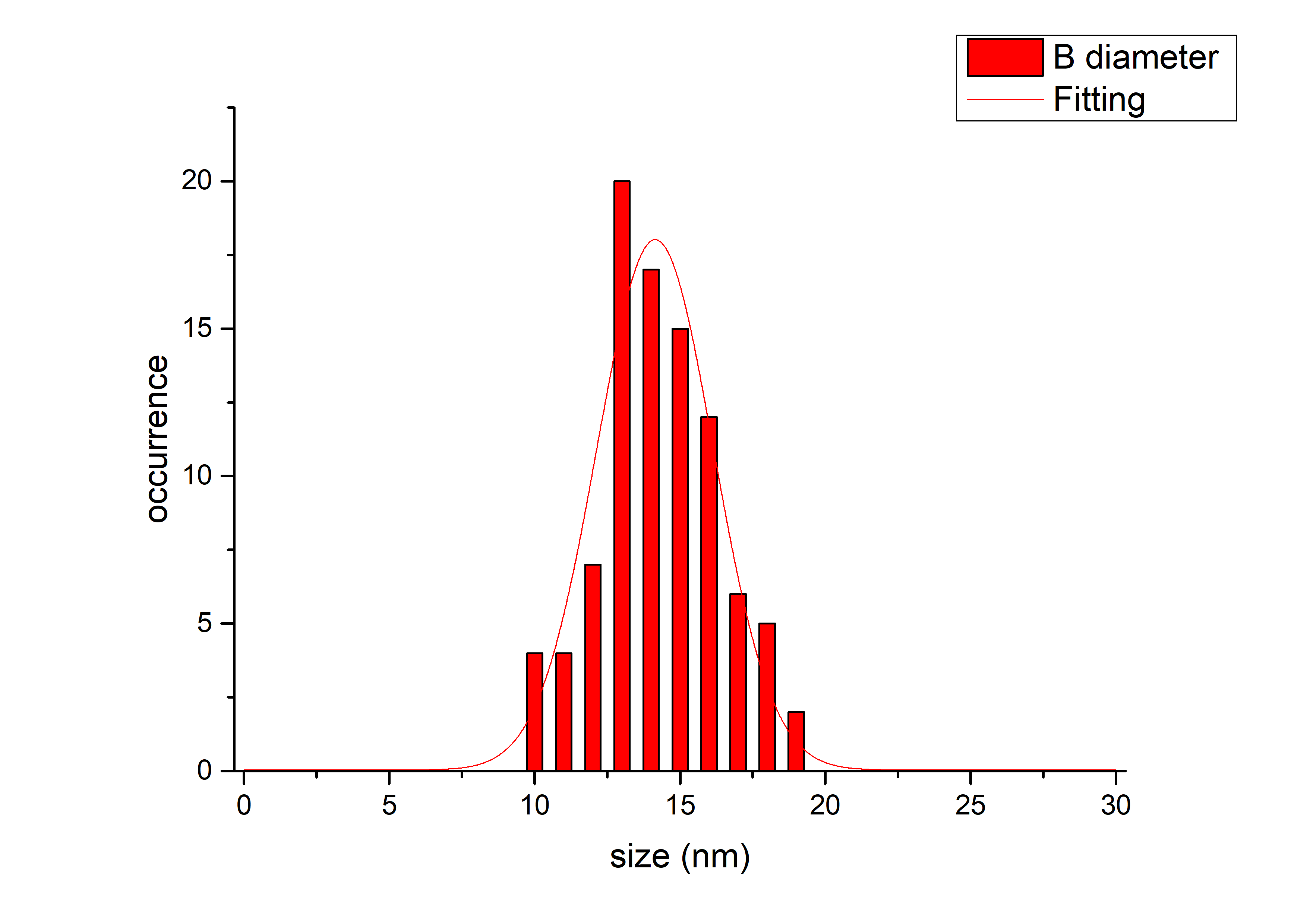


Figure S20. Sample B, diameter = 14 ± 2 nm


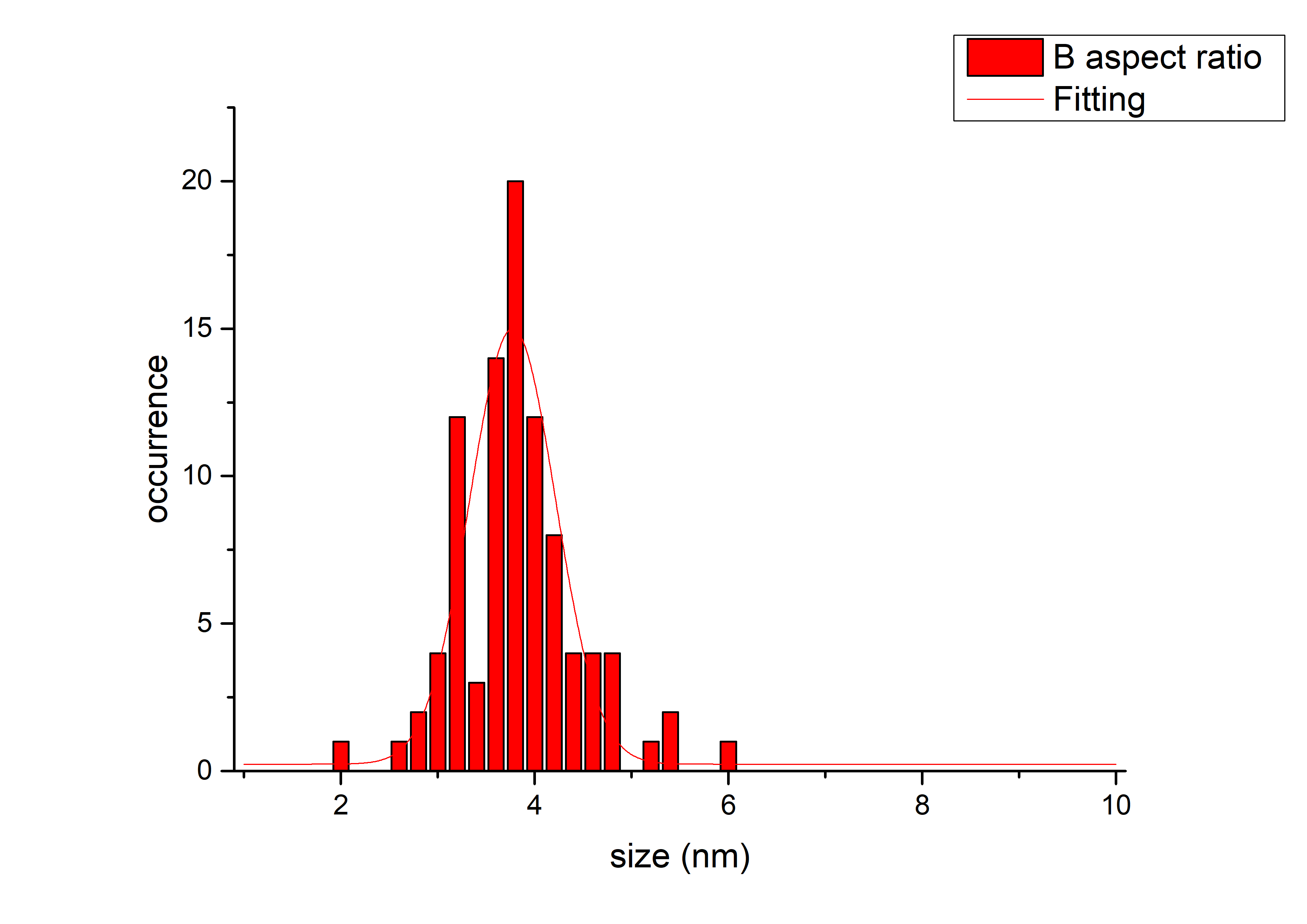


Figure S21. Sample B, aspect ratio = 3.7 ± 0.4 nm


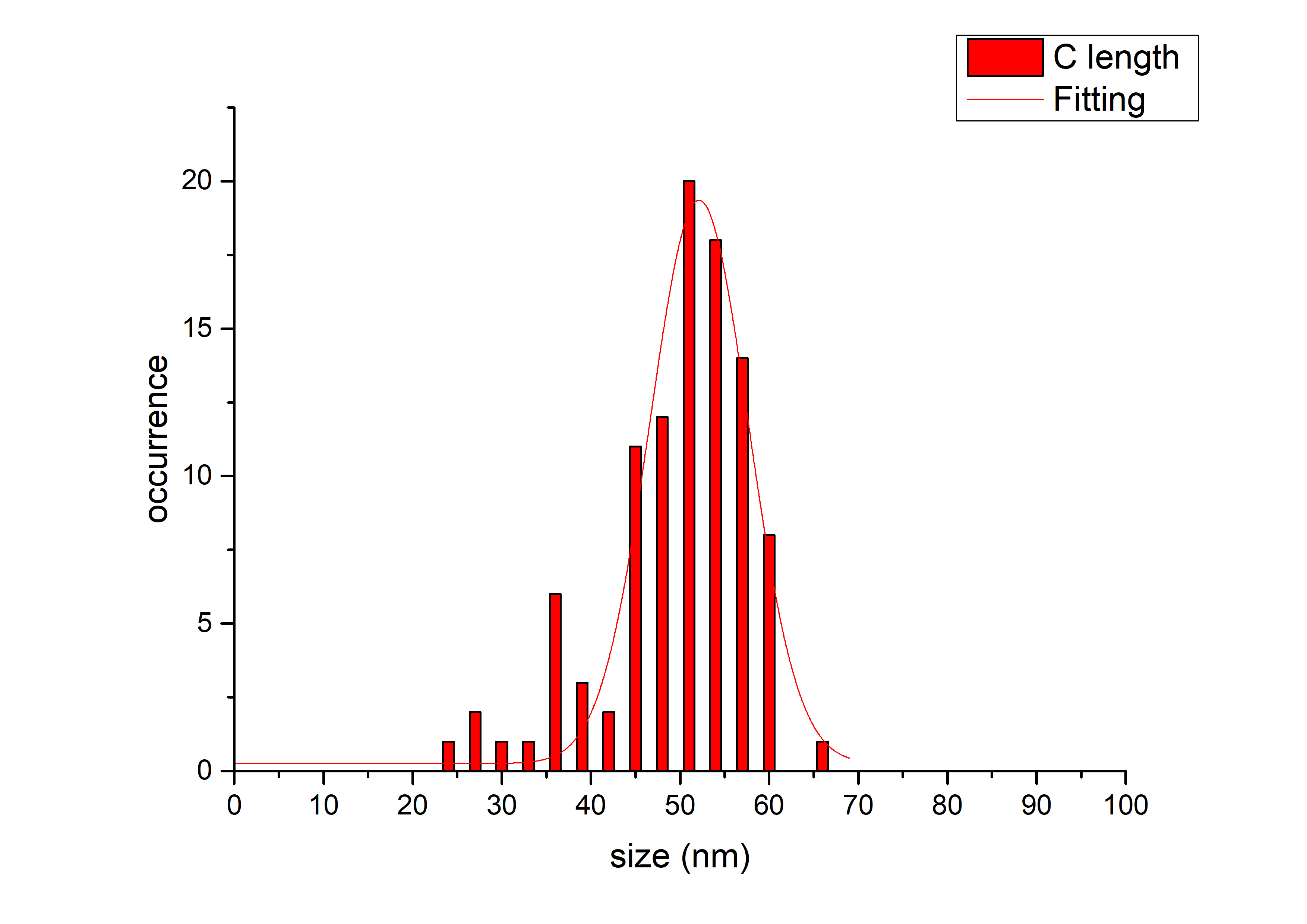


Figure S22. Sample C, length = 52 ± 6 nm


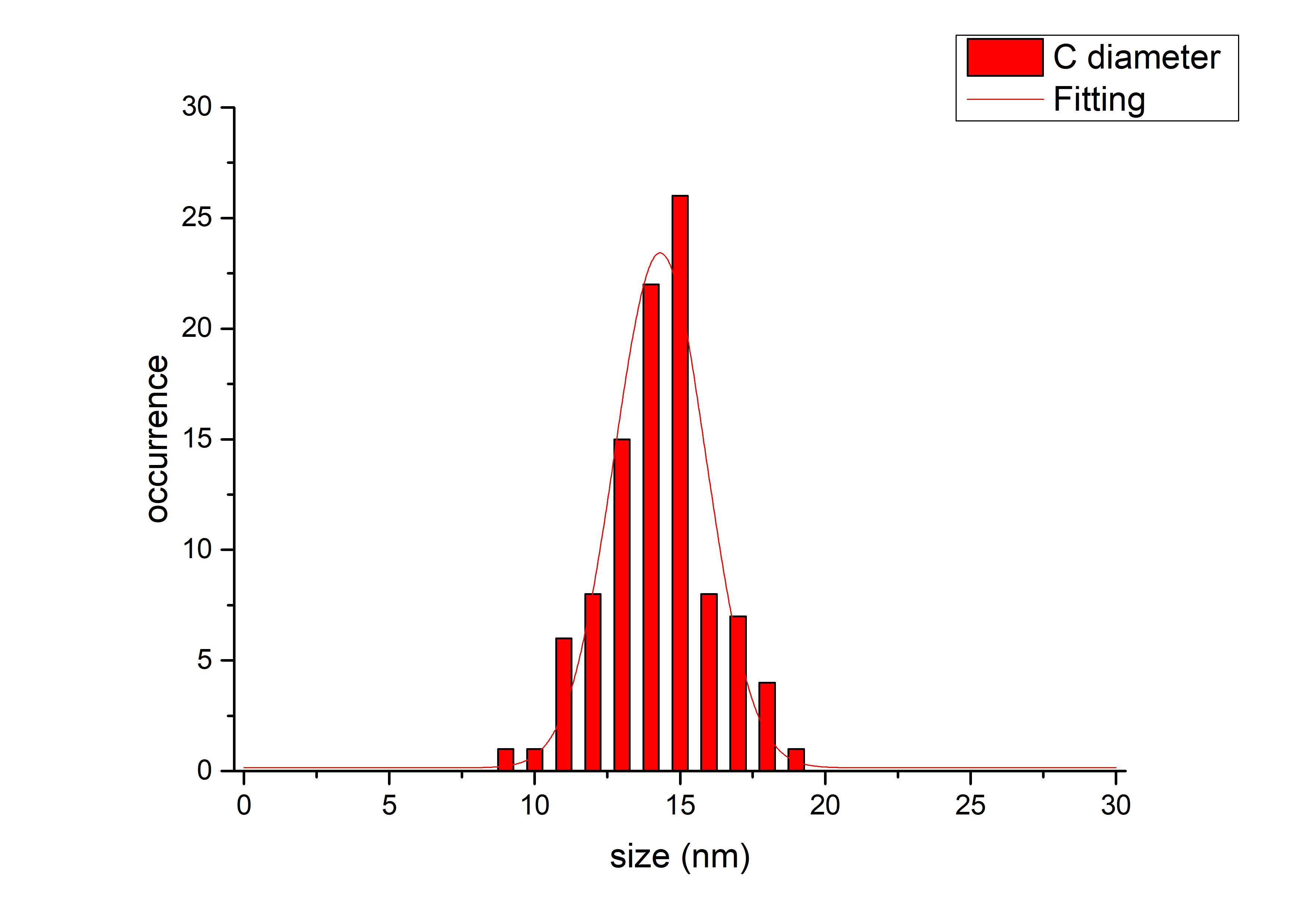


Figure S23. Sample C, diameter = 14 ± 2 nm


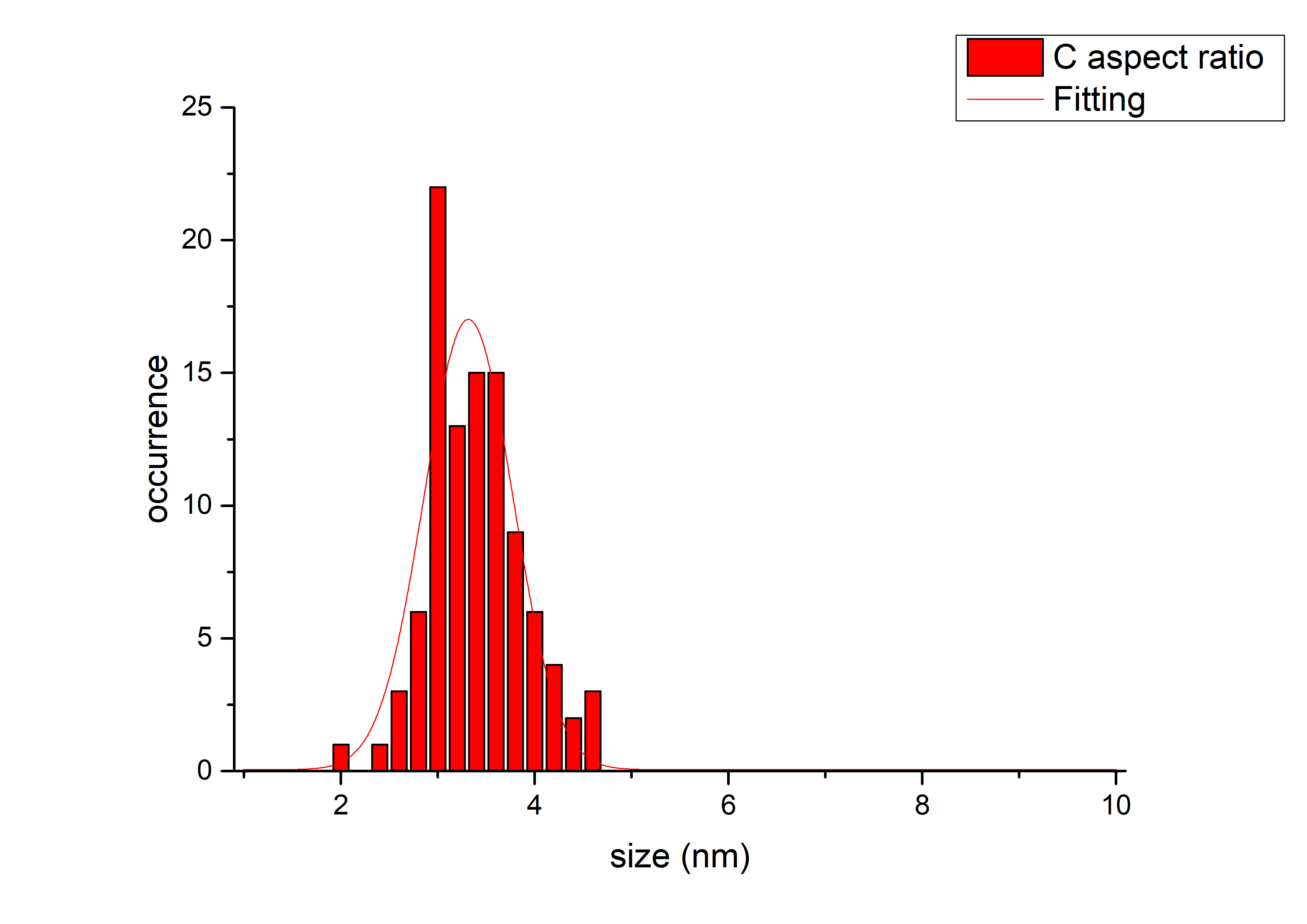


Figure S24. Sample C, aspect ratio = 3.3 ± 0.4 nm


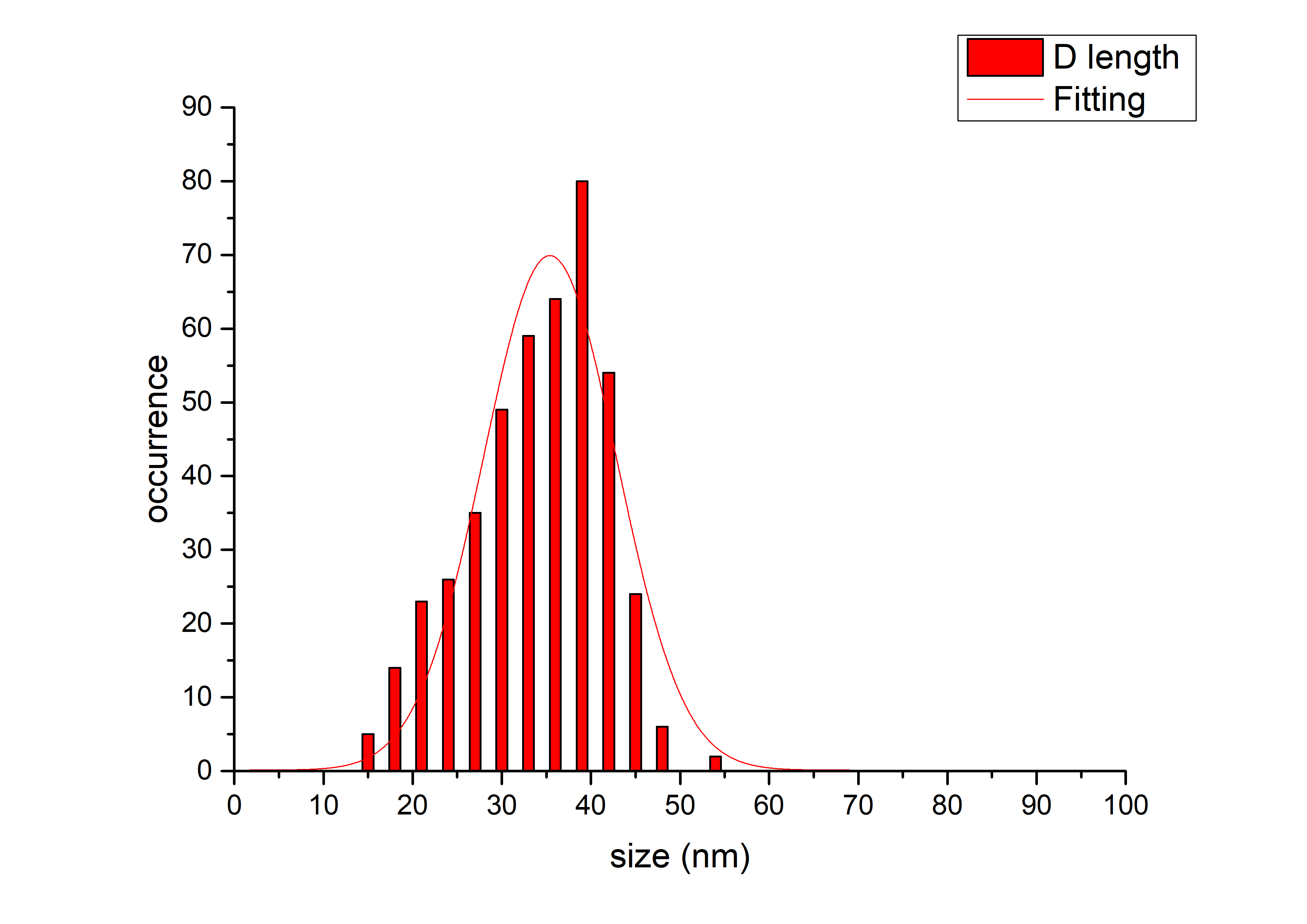


Figure S25. Sample D, length = 35 ± 7 nm


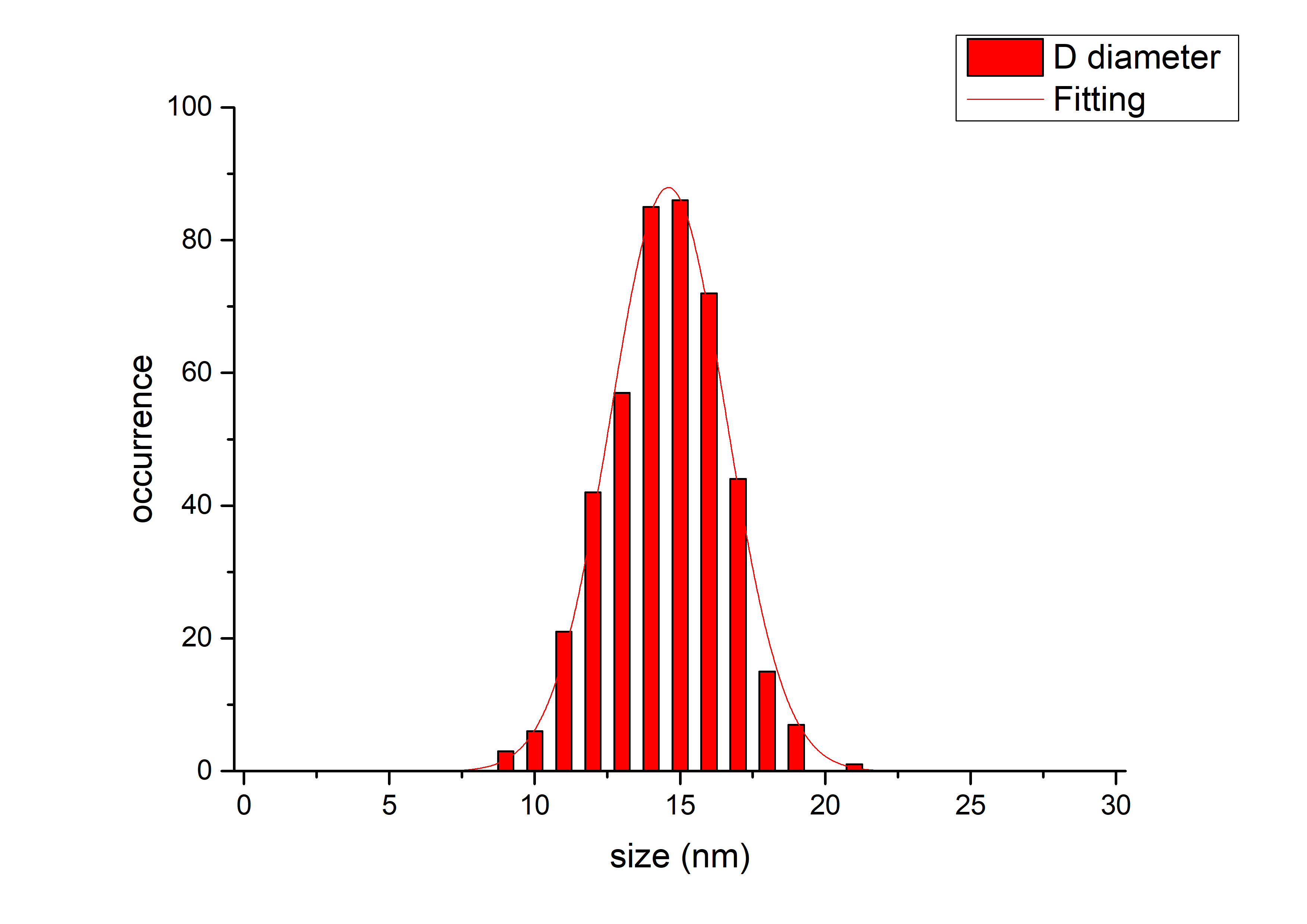


Figure S26. Sample D, diameter = 15 ± 2 nm


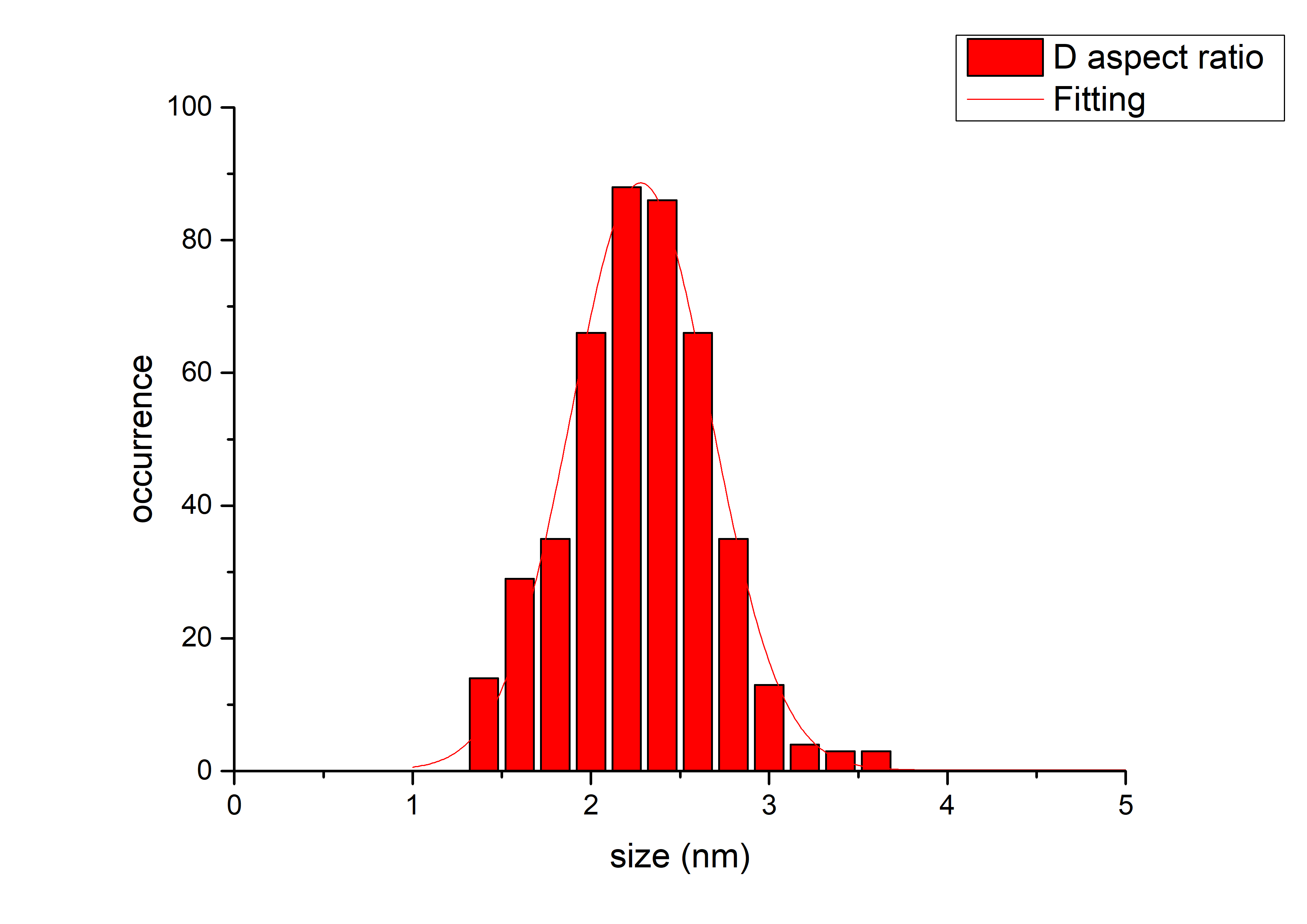


Figure S27. Sample D, aspect ratio = 2.3 ± 0.4 nm


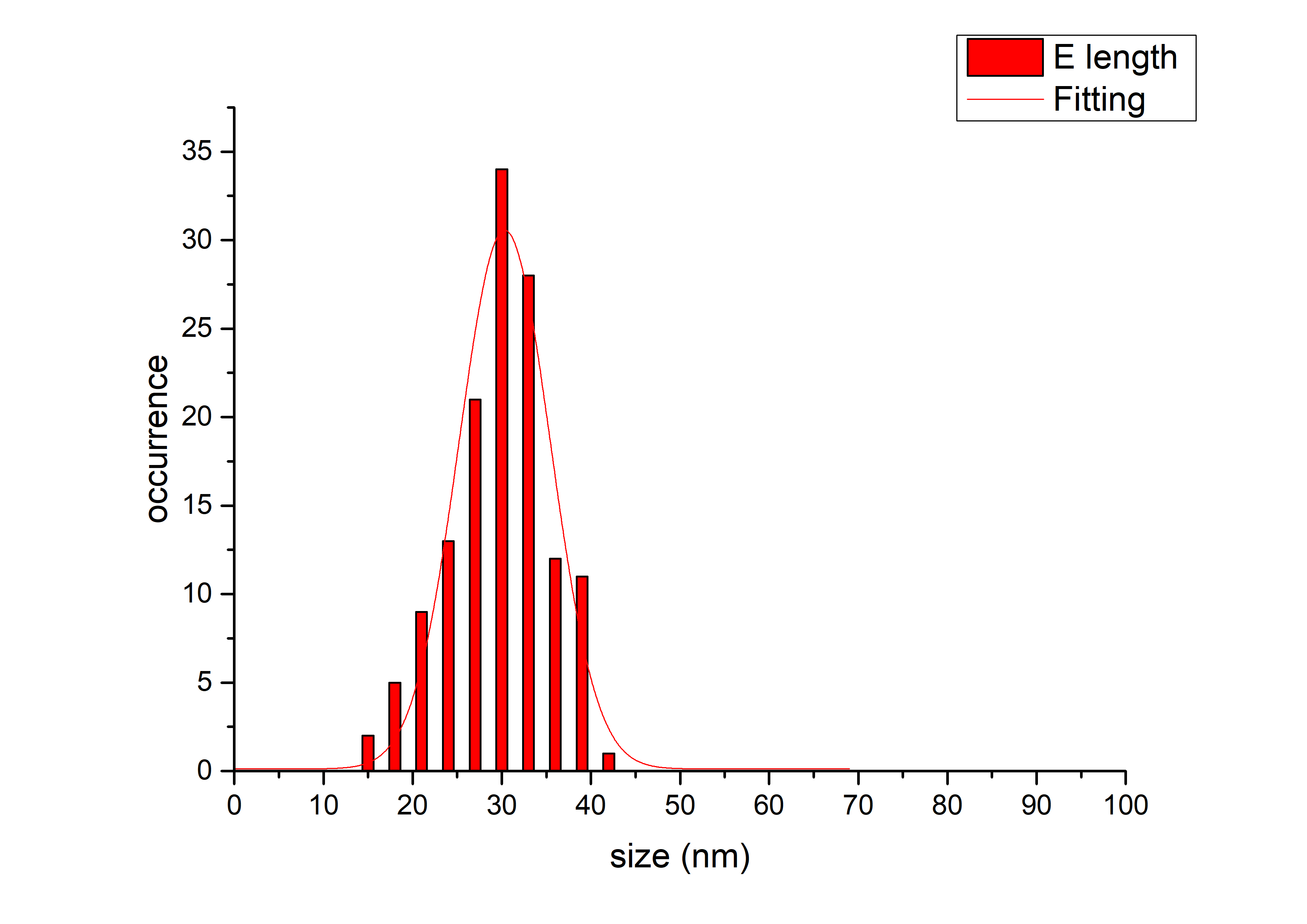


Figure S28. Sample E, length = 30 ± 5 nm


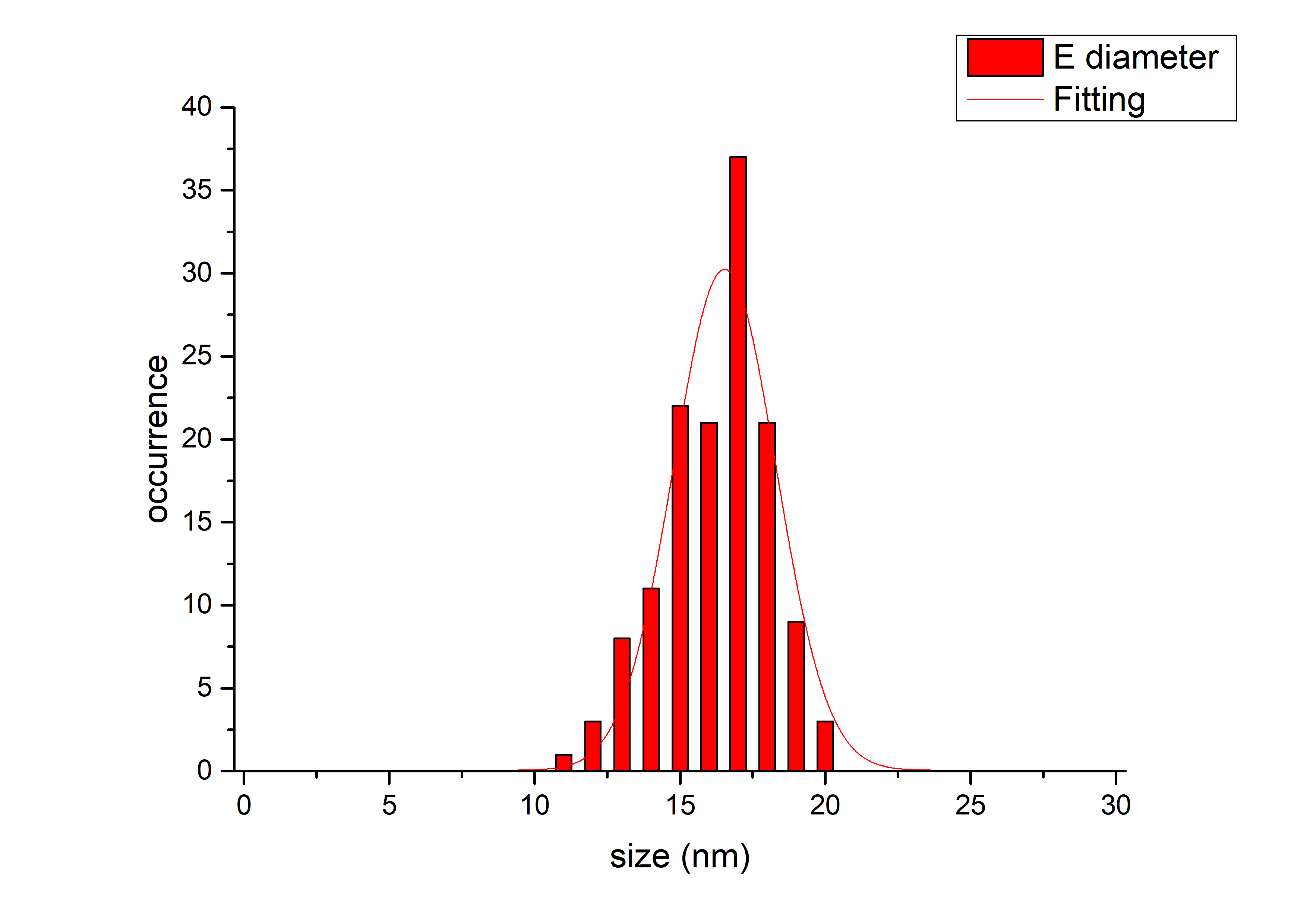


Figure S29. Sample E, diameter = 17 ± 2 nm


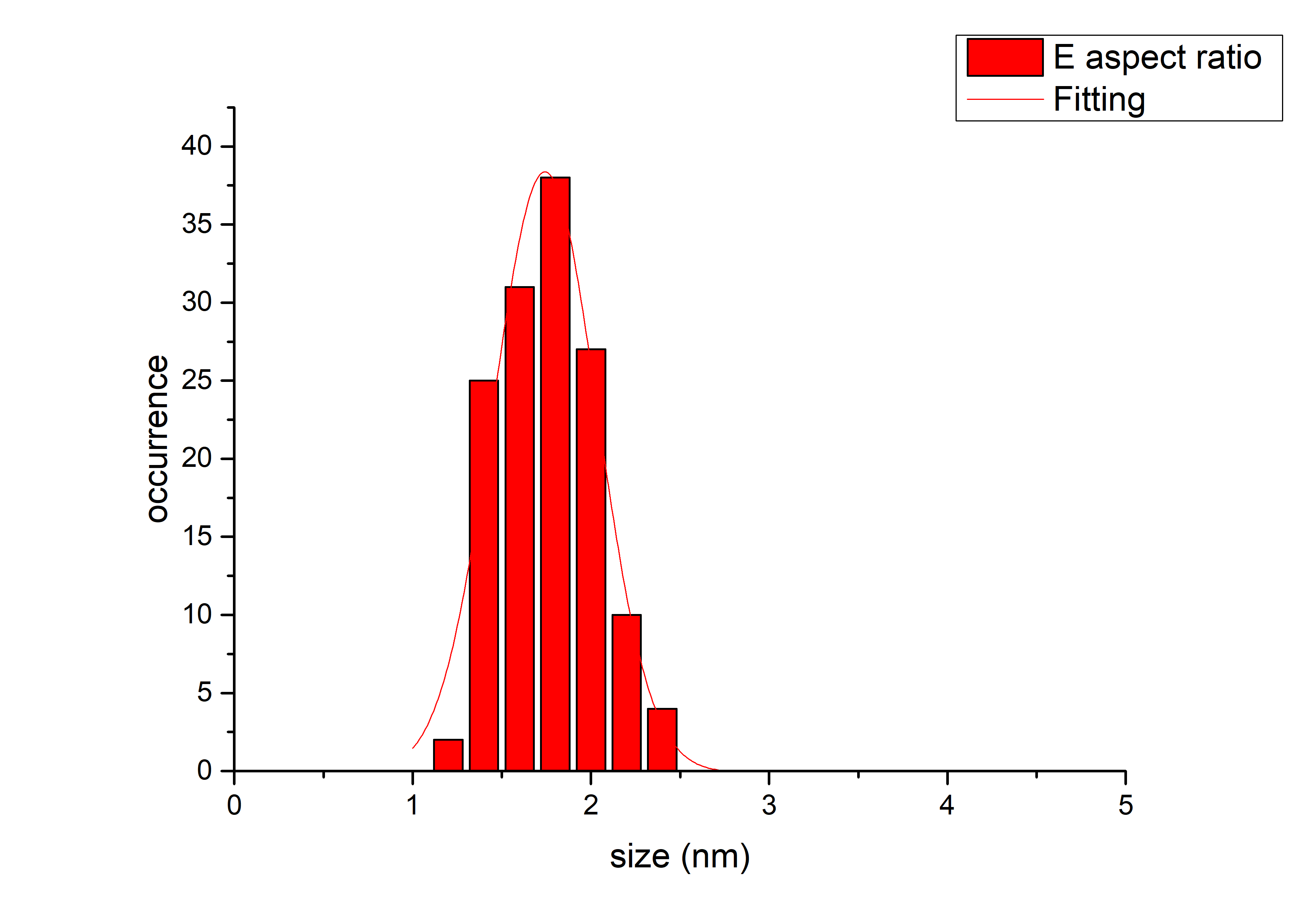


Figure S30. Sample E, aspect ratio = 1.7 ± 0.3 nm


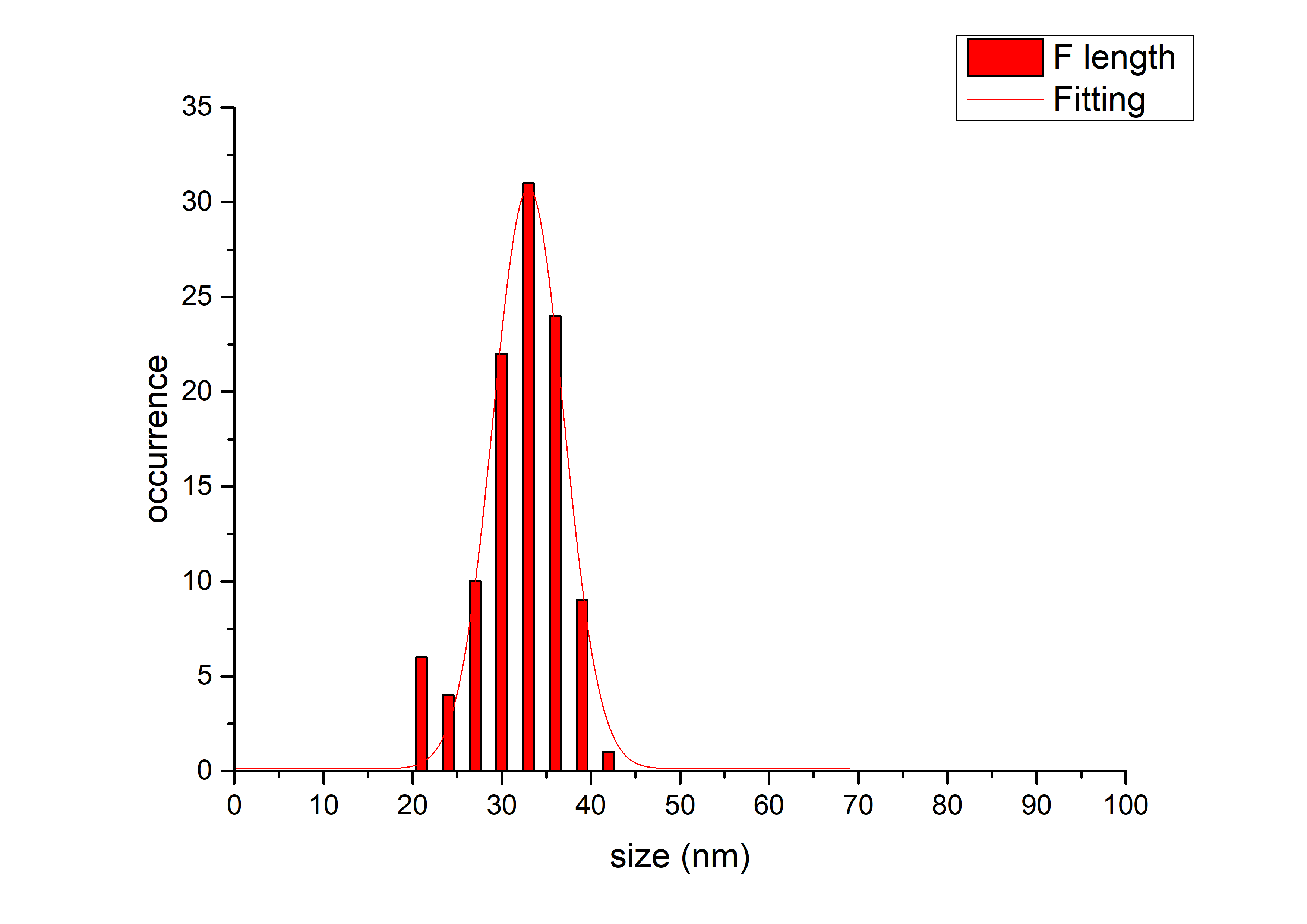


Figure S31. Sample F, length = 33 ± 4 nm


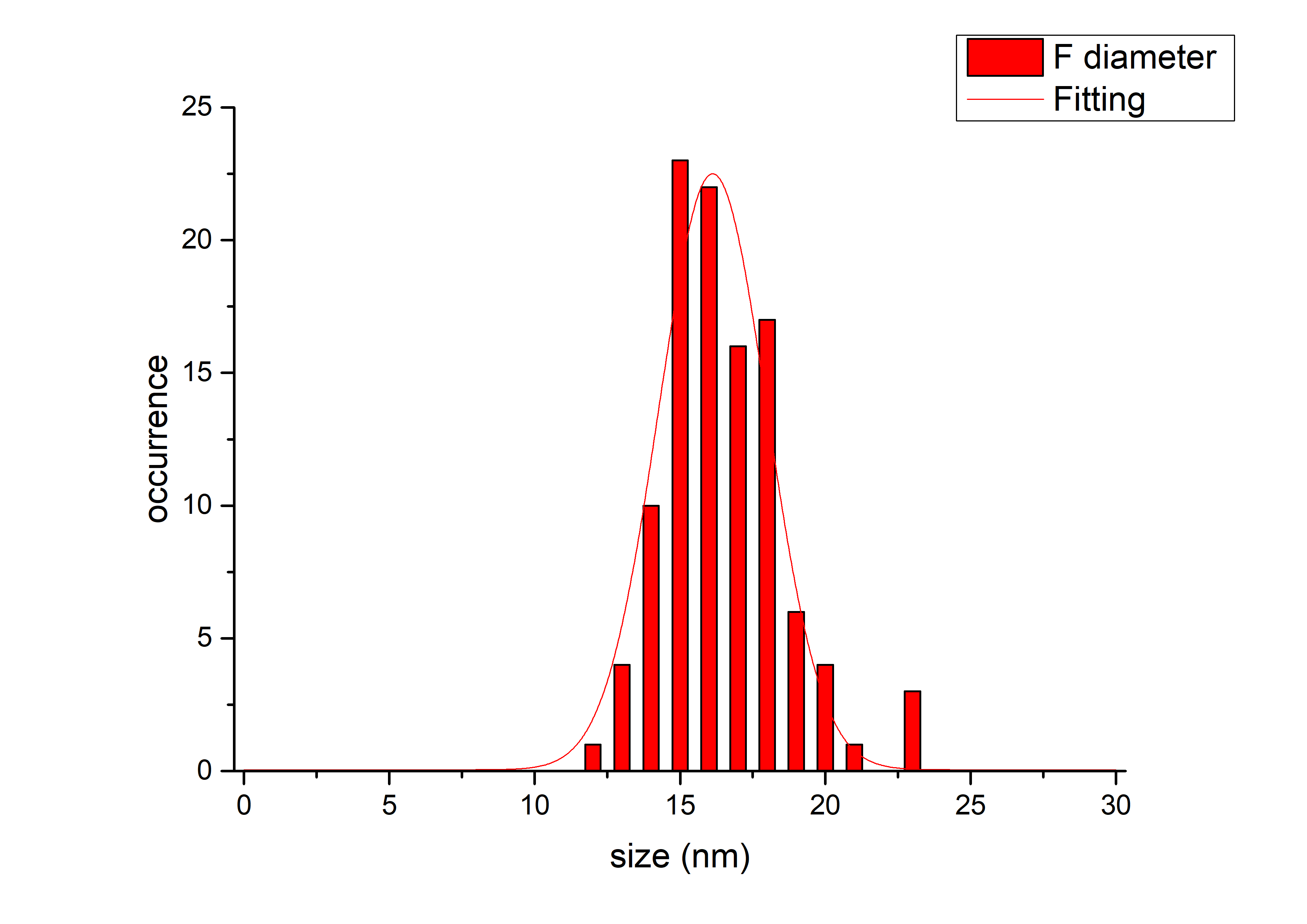


Figure S32. Sample F, diameter = 16 ± 2 nm


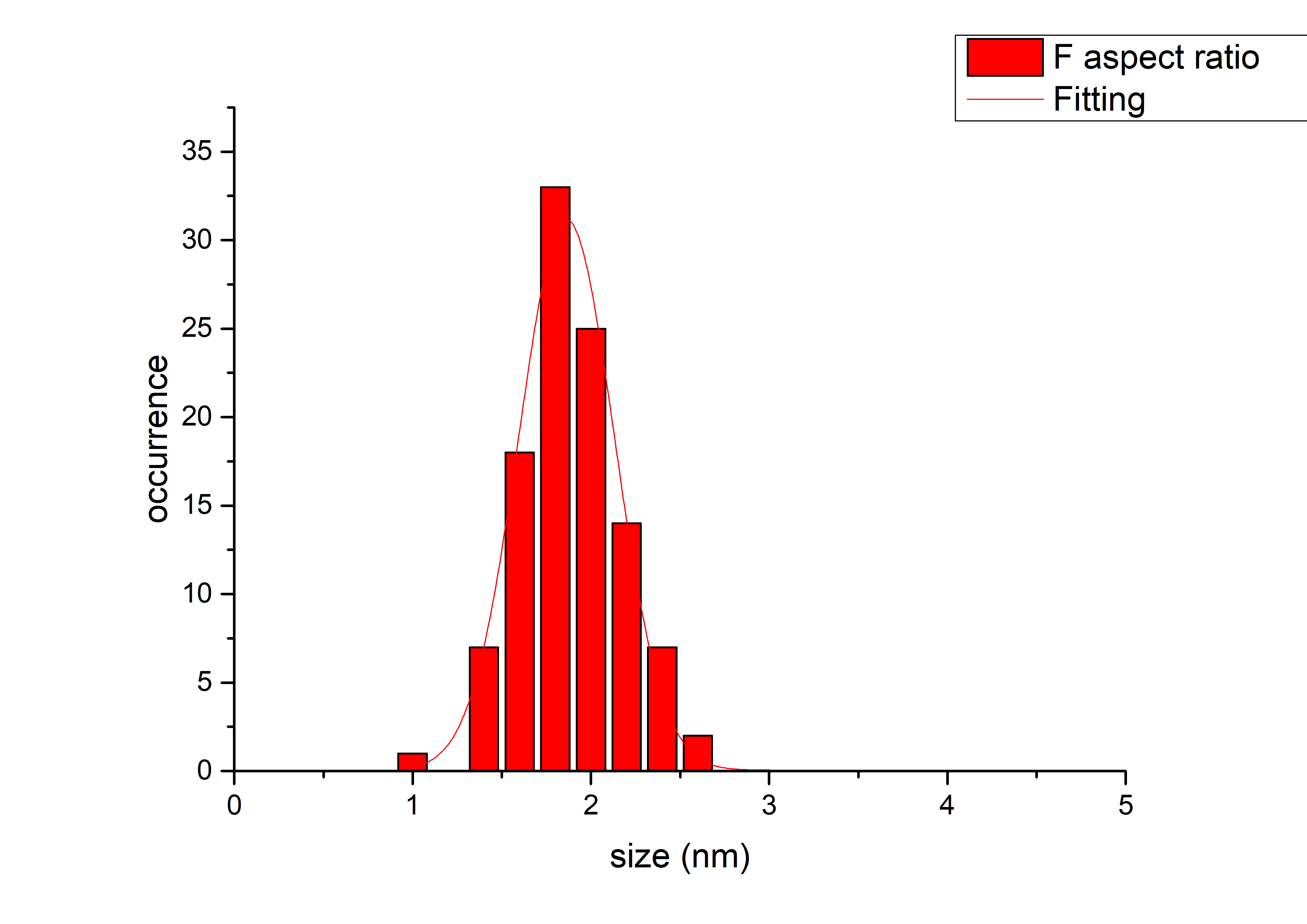


Figure S33. Sample F, aspect ratio = 1.9 ± 0.3 nm


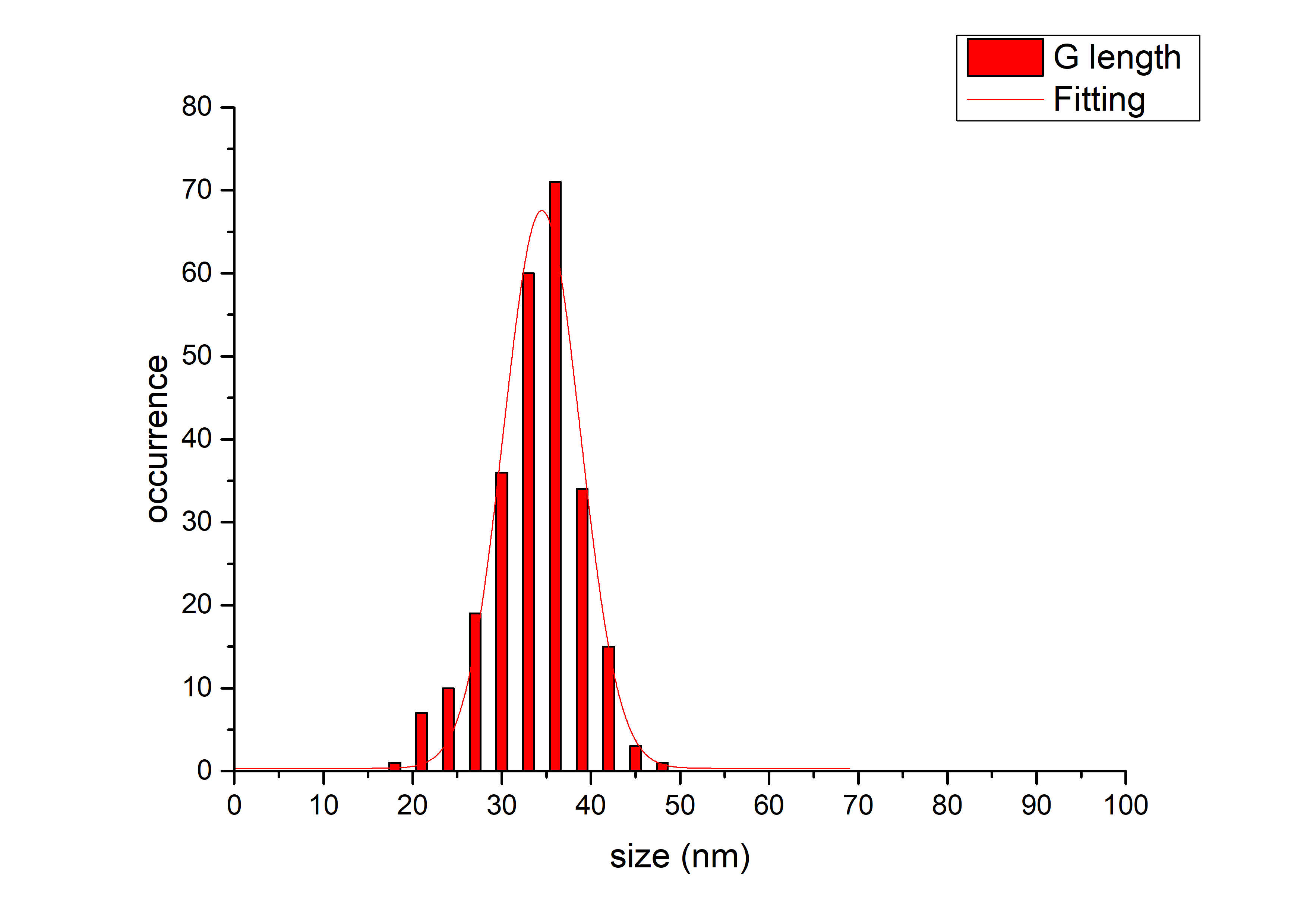


Figure S34. Sample G, length = 34 ± 4 nm


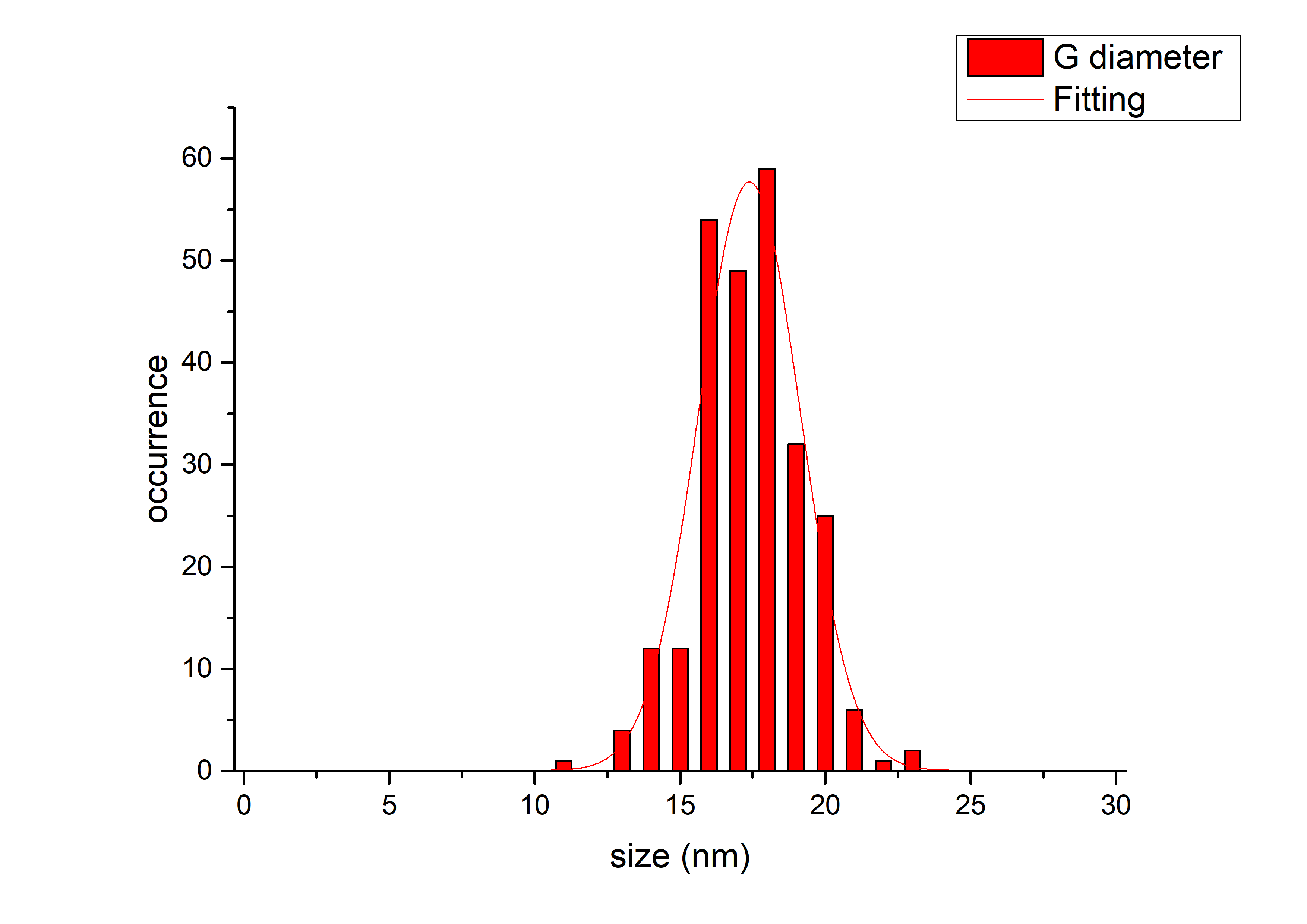


Figure S35. Sample G, diameter = 17 ± 2 nm


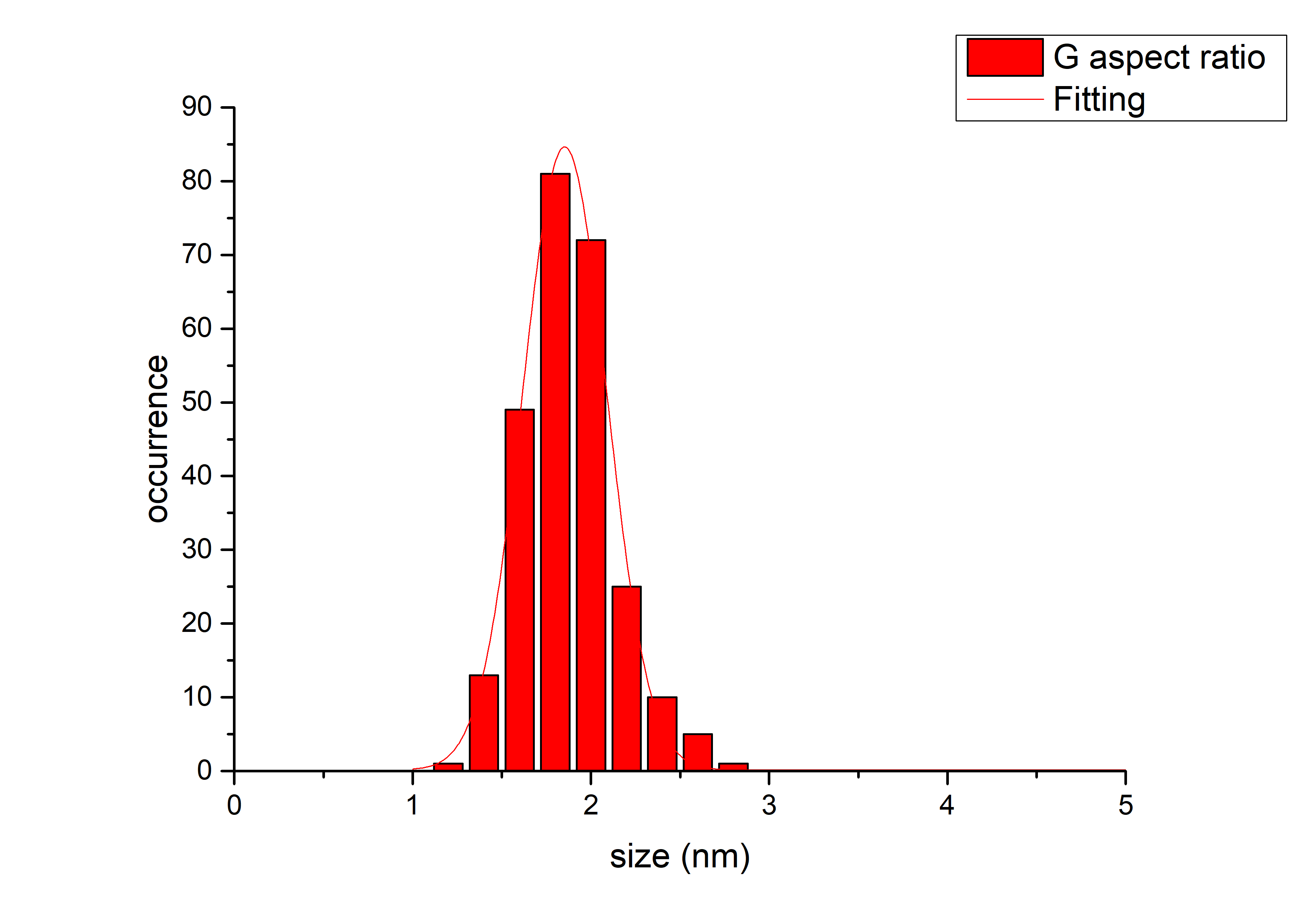


Figure S36. Sample G, aspect ratio = 1.9 ± 0.2 nm


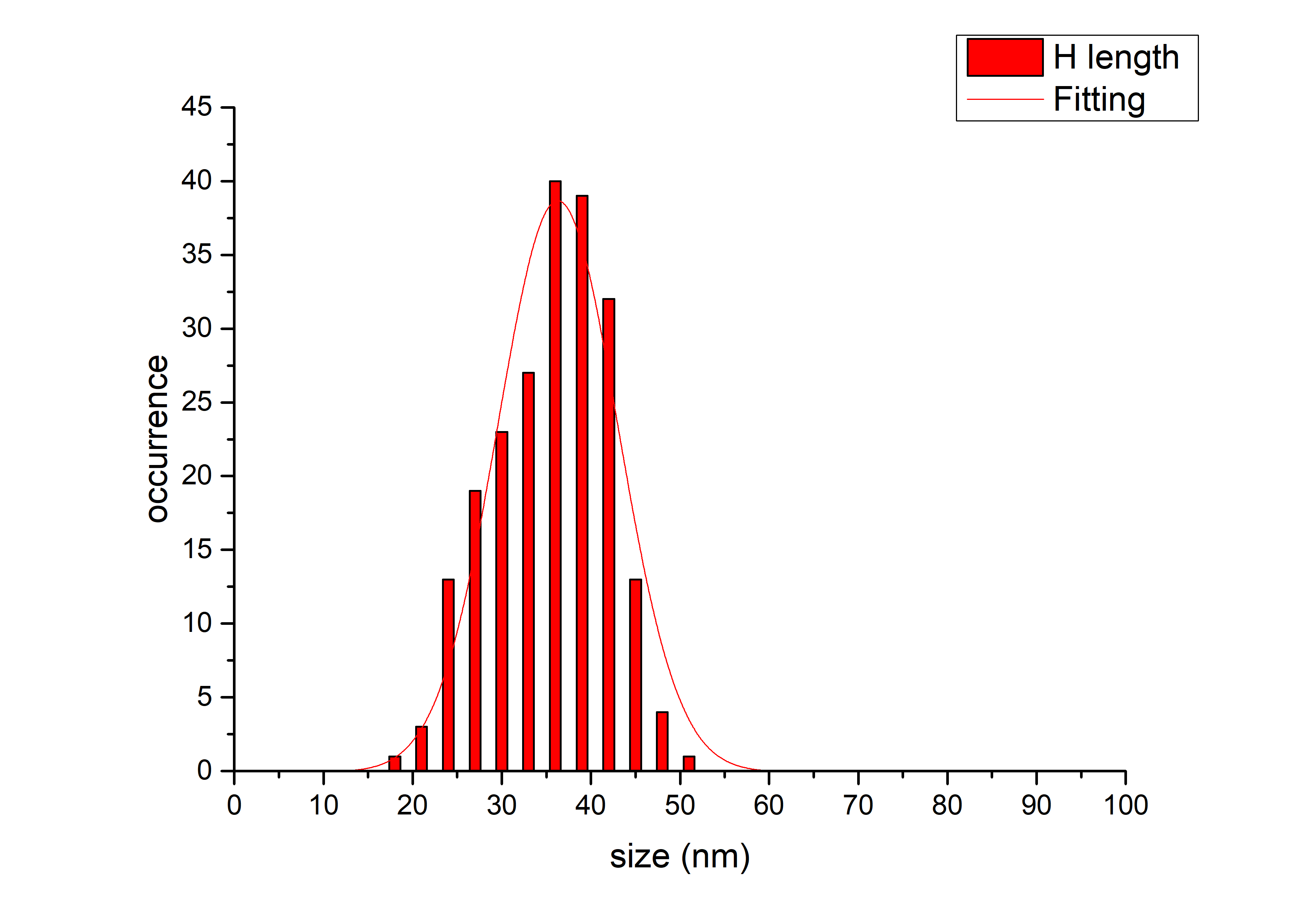


Figure S37. Sample H, length = 36 ± 7 nm


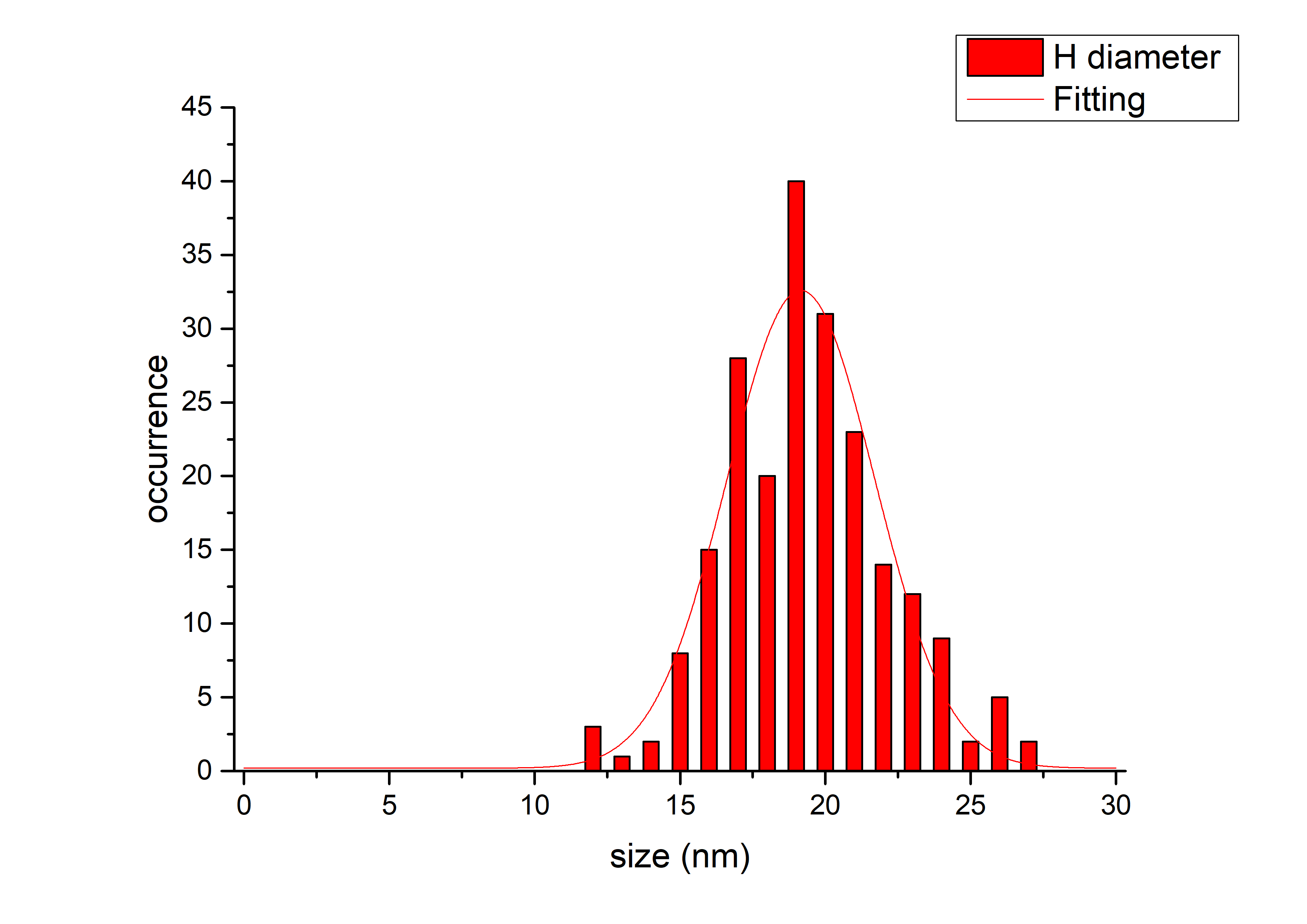


Figure S38. Sample H, diameter = 19 ± 3 nm


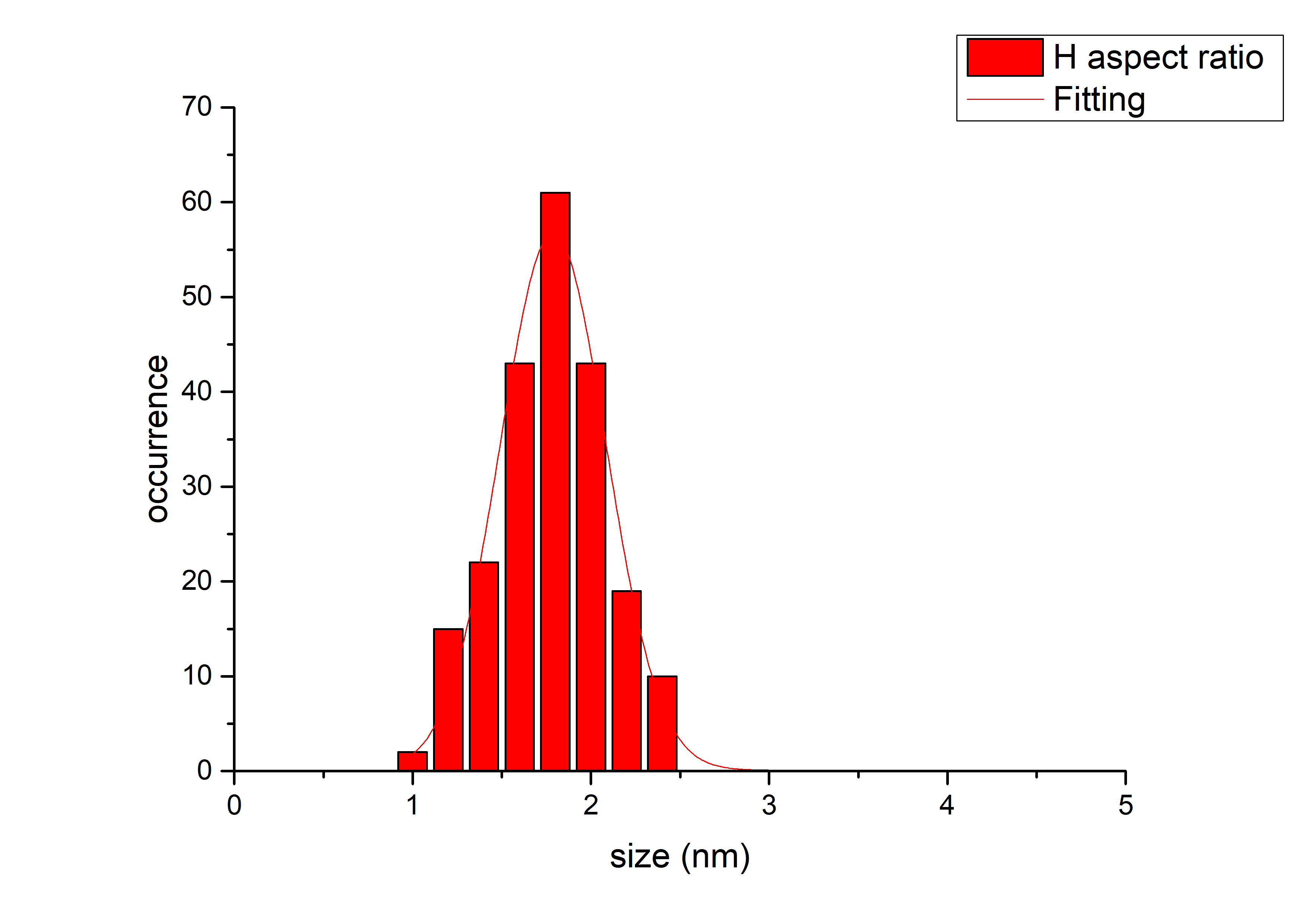


Figure S39. Sample H, aspect ratio = 1.8 ± 0.3 nm

1. **TEM images**


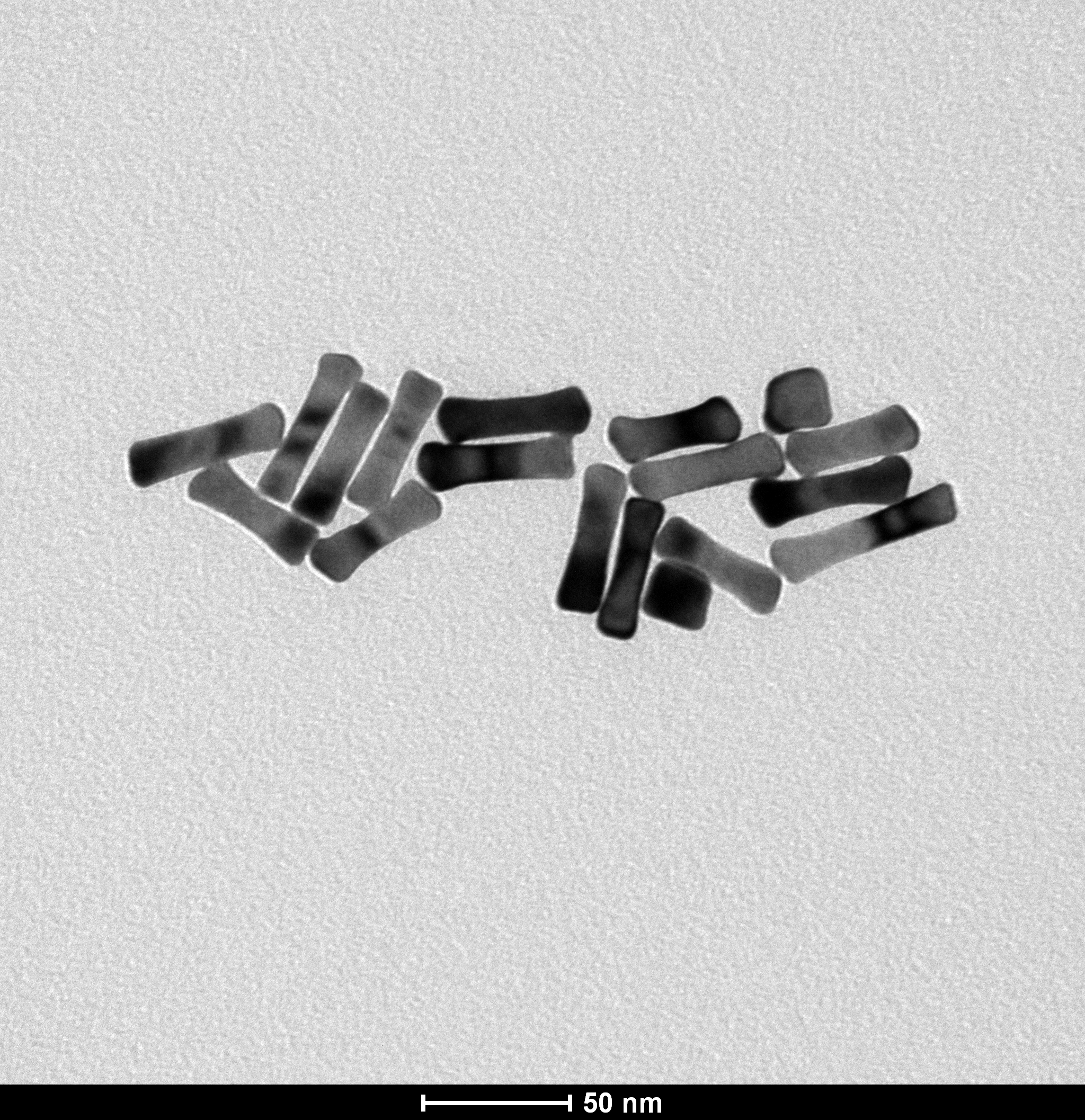


Figure S40: sample A (see Figure 8)


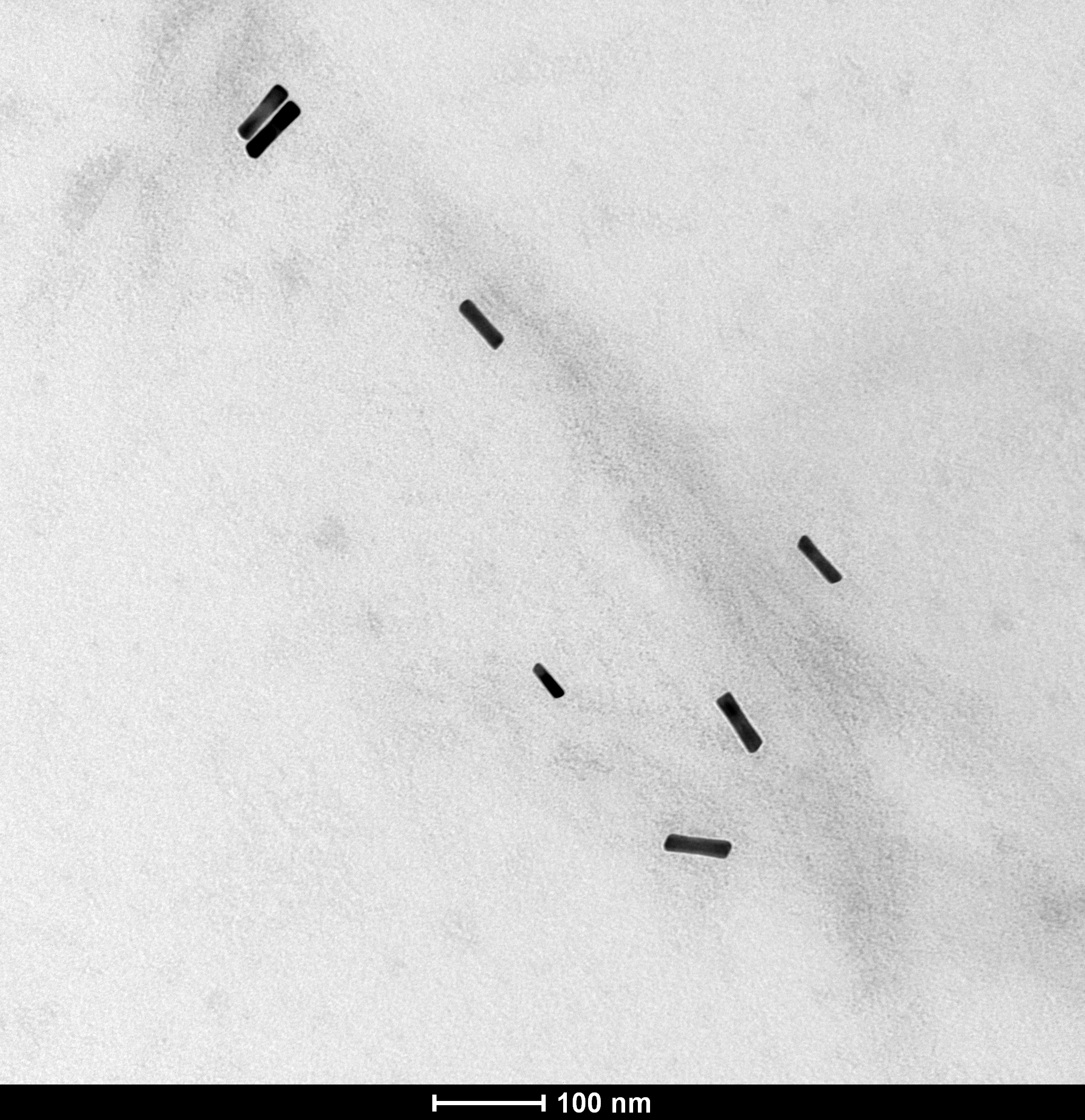


Figure S41: sample B (see Figure 8)


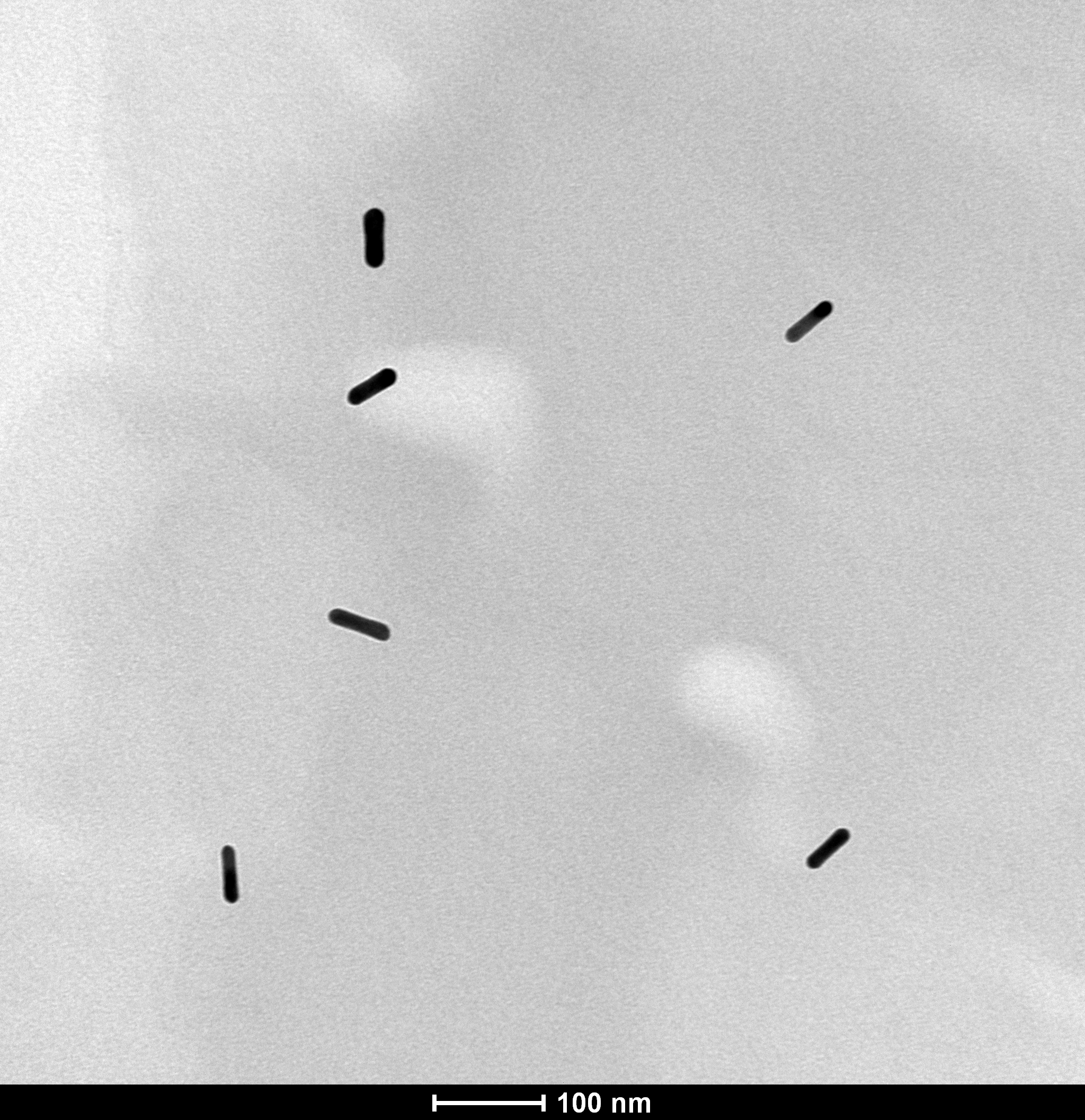


Figure S42: sample C (see Figure 8)


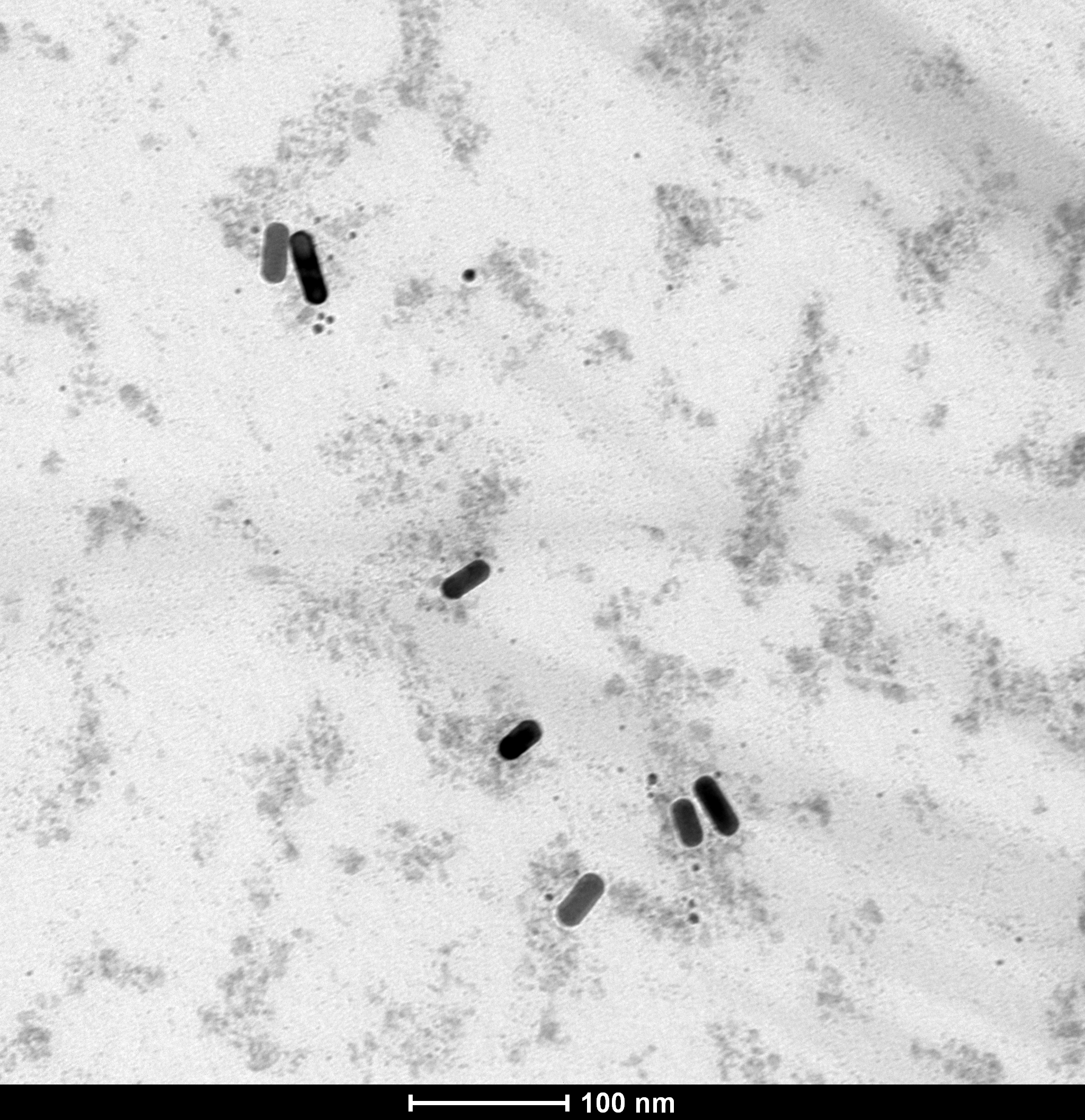


Figure S43: sample D (see Figure 8)


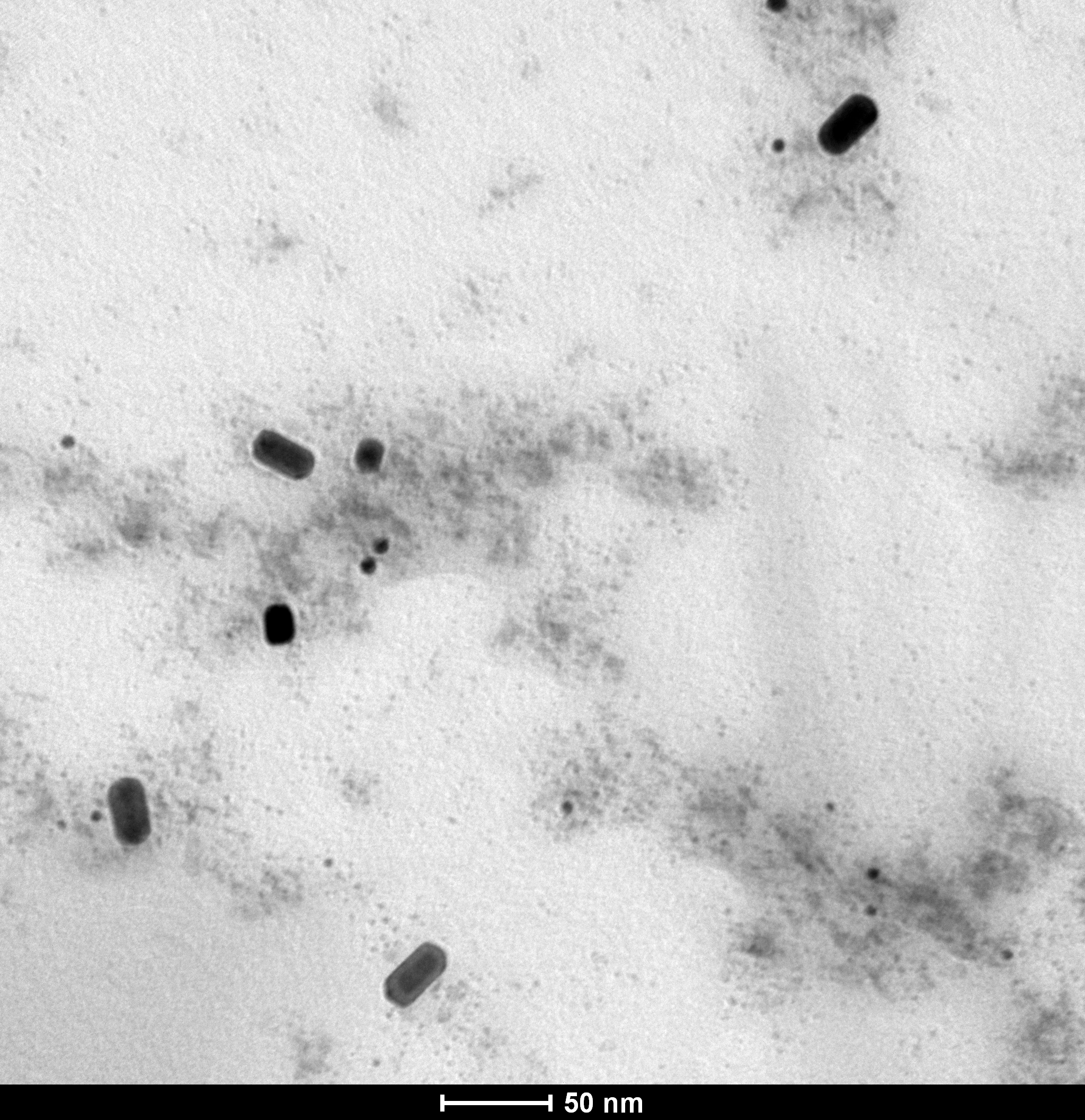


Figure S44: sample E (see Figure 8)


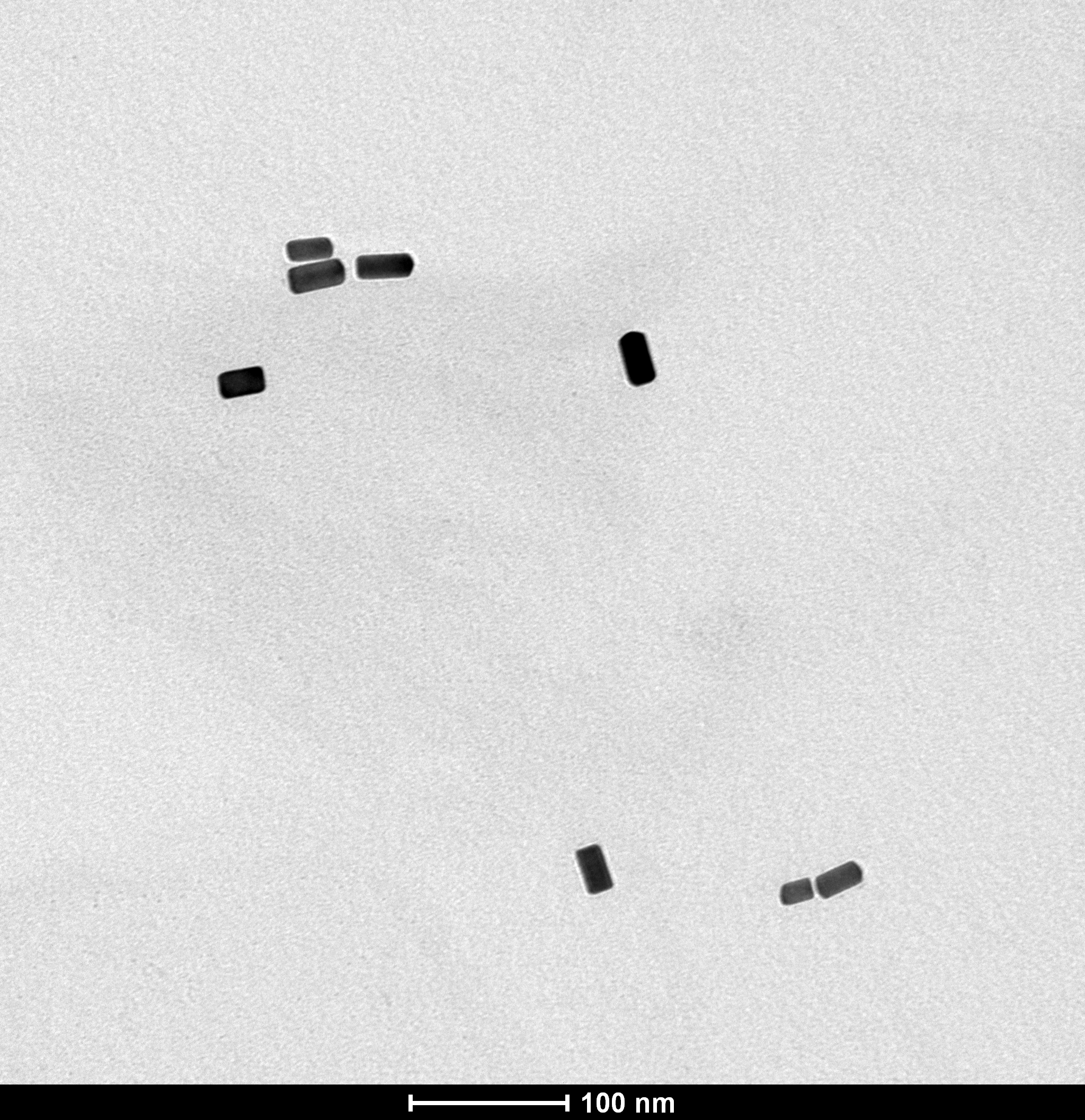


Figure S45: sample F (see Figure 8)


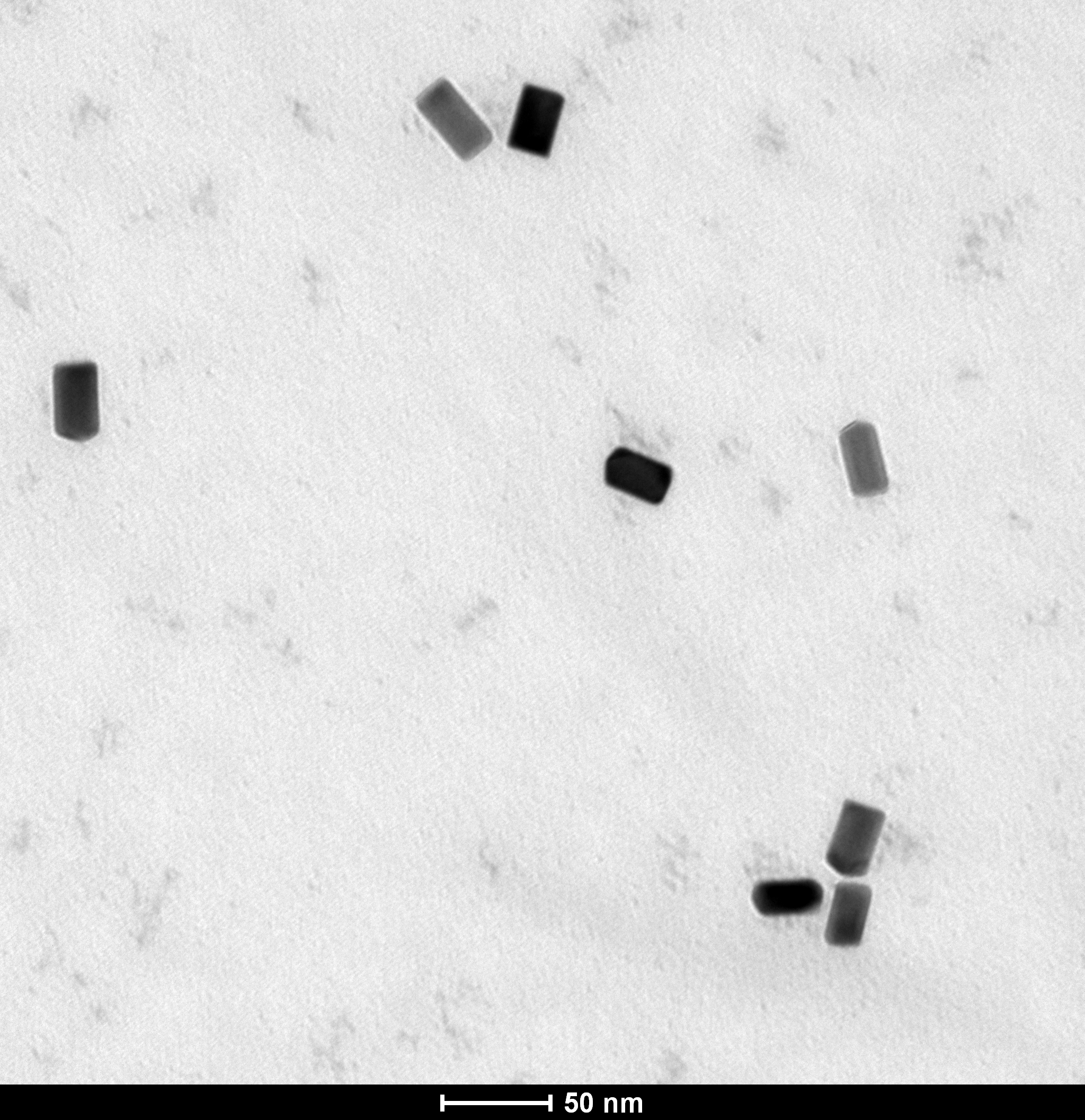


Figure S46: sample G (see Figure 8)


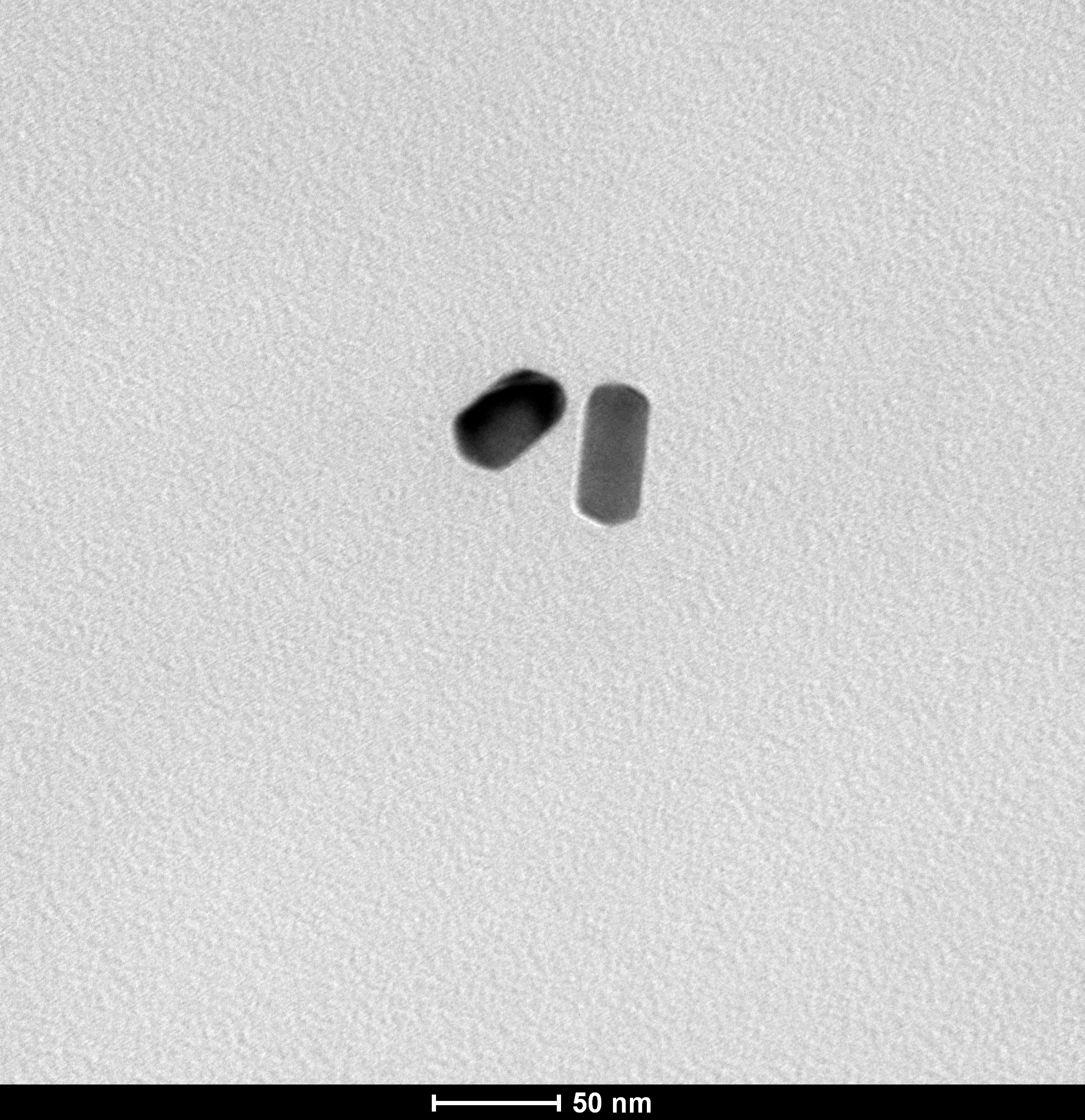


Figure S47: sample H (see Figure 8)


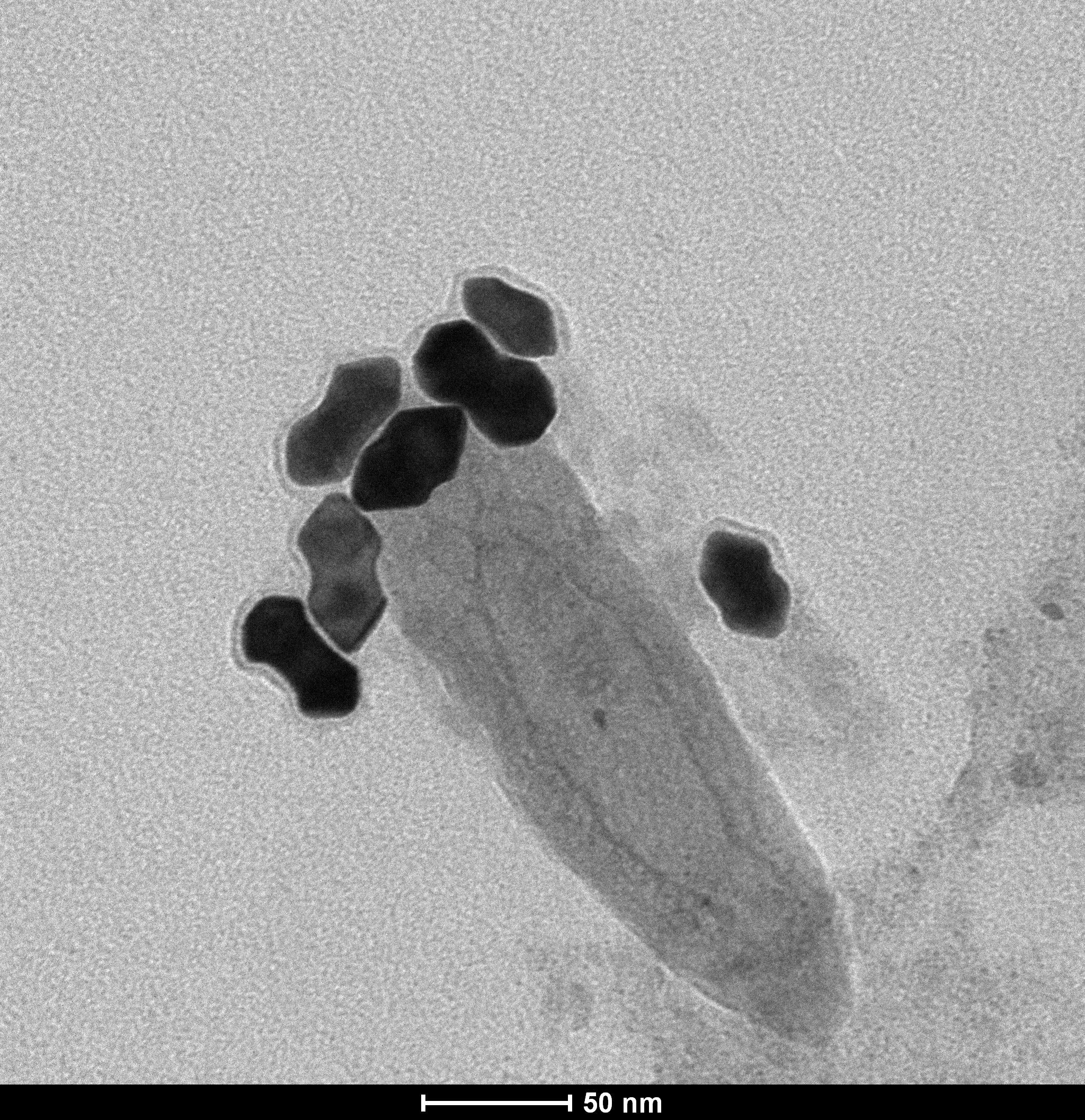


Figure S48: dumbbell-shaped nanoparticles with apparently crystalline terminations formed when attempting to quench Au(III) oxidants with metabisulfite (image used as inset in Figure 3).


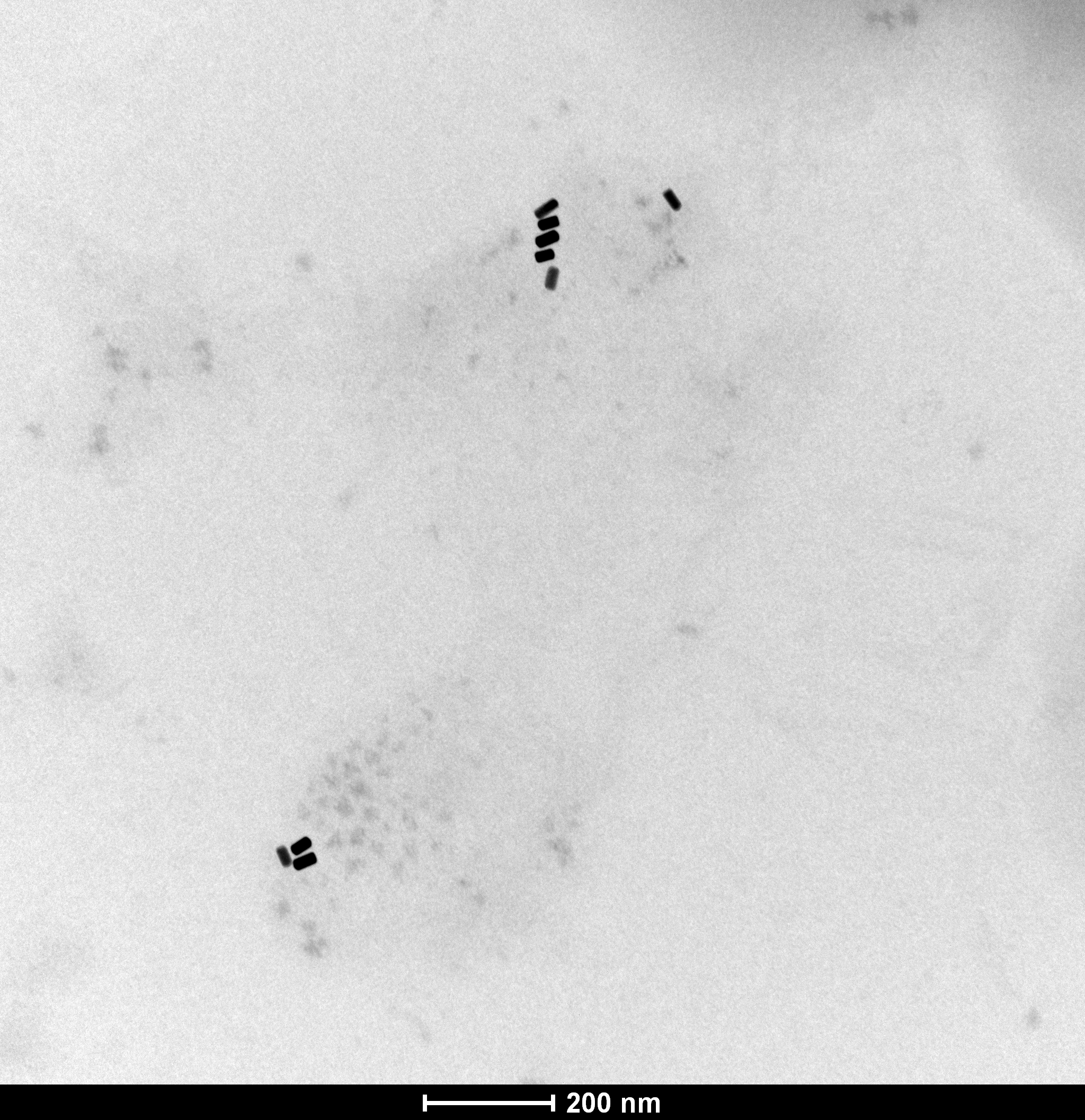


Figure S49: AuNRs after triiodide quenching with metabisulfite (image used as inset in Figure 4).
